# E2F1 suppresses Epstein-Barr virus lytic reactivation through cellular and viral transcriptional networks

**DOI:** 10.1371/journal.ppat.1013410

**Published:** 2025-08-07

**Authors:** Joyanta Biswas, SK. Asif Ali, Samaresh Malik, Subhadeep Nag, Piyali Mukherjee, Abhik Saha

**Affiliations:** Institute of Health Sciences, Presidency University, Kolkata, West Bengal, India; Brigham and Women's Hospital, UNITED STATES OF AMERICA

## Abstract

Epstein-Barr virus (EBV) establishes life-long persistence infection displaying a biphasic viral life cycle - latent phase and lytic replication. While latent EBV infection is linked to several B- and epithelial cell malignancies, periodic lytic-cycle reactivation is crucial for maintaining viral progeny and transmission. Targeting lytic reactivation offers a promising therapeutic avenue for EBV-associated cancers. Our genome-wide transcriptomic analysis reveals that E2F1 is transcriptionally activated during EBV latency but significantly suppressed during lytic reactivation. While ectopic E2F1 expression suppresses lytic replication, E2F1 depletion markedly accelerates this process. Mechanistically, we establish that E2F1 and the lytic transactivator BZLF1 form a negative transcriptional feedback loop, tightly controlling viral lytic replication. Furthermore, E2F1 positively regulates c-Myc expression and together they repress leaky BZLF1 expression during latency. Notably, c-Myc does not influence E2F1 expression, nor does BZLF1 modulate c-Myc transcription, underlining a distinct regulatory hierarchy. In sum, our findings reveal that EBV tightly controls the latent-to-lytic switch through precise regulation of E2F1 expression, positioning E2F1 as a pivotal regulator of both cellular and viral gene expression.

## Author summary

Epstein-Barr virus (EBV) is linked to several B-cell and epithelial cancers, with its ability to switch between dormant (latent) and active (lytic) phases playing a crucial role in infection. Inducing the lytic cycle, followed by antiviral treatment, is a promising approach for treating EBV-associated cancers. Understanding the mechanisms that regulate this switch is essential for developing new targeted therapies. Our study identifies E2F1 as a key regulator of EBV’s life cycle – during latency E2F1 expression is elevated, but suppressed during lytic activation. We found that while E2F1 depletion accelerates viral lytic cycle, enhanced E2F1 expression prevents it. E2F1 and the key viral lytic protein BZLF1 form a feedback loop that tightly controls this process. Additionally, E2F1 enhances c-Myc expression, and together they suppress BZLF1 during latency. However, c-Myc and BZLF1 do not regulate each other directly. These findings position E2F1 as a critical factor in controlling EBV’s transition between latent and lytic phases.

## Introduction

Epstein-Barr virus (EBV), member of the gammaherpesvirus family, persistently infects more than 95% of the global population [1,2]. Like any other herpesviruses, EBV similarly displays a biphasic life cycle comprising latent infection and lytic replication, both of which play crucial roles in its pathogenesis [3,4]. While latent infection is associated with numerous cancers both of epithelial and lymphoid origin, EBV reactivation into lytic cycle replication is indispensable to maintain the production of progeny virus [4,5]. A central feature of EBV latent infection is its ability to transform metabolically dormant B-lymphocytes into hyper-proliferating B-cell blasts, followed by the establishment of distinct latency programs (I, II, and III), each characterized by unique signatures of viral latent oncogene expression [1,6]. EBV employs these latency programs that ultimately direct B-cell blasts to enter germinal centre and differentiate into memory B-cells, which serve as the reservoir for lifelong infection [7]. The restricted expression of EBV latent genes eases immune surveillance and is a hallmark of its association with various B-cell malignancies, such as Burkitt’s lymphoma (BL), Hodgkin lymphoma (HL) and diffuse large B-cell lymphoma (DLBCL) [5]. Studies reveal a significant connection between EBV-associated malignancies and the compromised host immune system, leading to post-transplant lymphoproliferative disorders (PTLDs) in immunosuppressive individuals, including organ transplant recipients and AIDS patients [1,5].

In the germinal B-centre, infected memory B-cells can intermittently terminally differentiate into plasma cells and thereby triggering EBV lytic cycle reactivation, which is critical for sustaining the viral reservoir and promoting horizontal transmission from one host to another [8]. This process involves re-expression of ~80 viral genes required for active replication and the production of viral progeny [9]. EBV lytic gene expression occurs in a temporally regulated cascade, which can be broadly divided into four phases - immediate-early (IE), early (E), leaky late (LL), and late (L). The lytic cycle replication initiates by transcriptional activation of one or both of its IE promoters - Zp and Rp, leading to expression of two IE genes, BZLF1 (encoding Zta) and BRLF1 (encoding Rta), respectively [9,10]. Although both are transcription factors (TFs) and can transactivate each other’s promoters to initiate viral reactivation, in most of the EBV positive cells BZLF1 can solely orchestrate EBV lytic cycle replication by provoking transcriptional activation of nearly 30 E genes [11,12]. Newly replicated viral DNA then serves as templates for further transcriptions of L lytic genes. Some L-phase genes, referred to as LL, may begin to be expressed, albeit at lower levels, even before the initiation of viral genome replication, in contrast to true L genes. Expression of these genes, both LL and L, facilitate encapsidation of viral dsDNA, virion assembly at the cell membrane, and egression of mature infectious particles [9–11]. In contrast to latent genes, EBV lytic antigens are highly immunogenic, eliciting strong immune responses. However, the function of several lytic immune evasion genes directly contributes to minimizing immune detection and ensuring efficient viral replication [13].

As yet, there are no clinically approved therapies available that selectively targets EBV associated cancers. Induction of EBV lytic cycle followed by treatment with antiviral drugs offers an attractive therapeutic approach to treat EBV associated cancers [14]. This targeted ‘lytic induction therapy’ functions by triggering lytic cycle replication, during which two viral protein kinases BXLF1 and BGLF4 phosphorylate nucleoside analogues like acyclovir and ganciclovir, converting the pro-drugs into their active nucleotide forms, resulting in cytotoxicity in both EBV-infected and neighbouring cells [15]. Therefore, there is increasing interest in strategies that drive EBV reactivation from latency into the lytic cycle, thereby sensitizing EBV-positive tumour cells to these nucleoside analogues. However, the existing methods of EBV lytic cycle reactivation by chemical inducers are highly cytotoxic and typically lytic replication occurs in only a small percentage of latently infected cells [4]. To date, among the growing list of EBV lytic cycle inducers, only a few have been tested in clinical trials, but failed due to high cytotoxicity and incongruous pharmacokinetics [14]. A proper understanding of cell mechanisms controlling the balance between latent infection and lytic replication is therefore critical for further development of potential drugs that selectively kill EBV positive tumour cells. EBV latent-to-lytic switch is tightly controlled by several cellular factors, including TFs, chromatin remodelling, and signalling pathways [10]. For example, during B-cell differentiation into plasma cells, BLIMP1/PRDM1 and XBP-1 TFs induce spontaneous EBV lytic replication by transcriptional activation of both IE genes - BZLF1 and BRLF1 [16,17]. Recently, genome-wide CRISPR-Cas9 screening in EBV-positive BL cells identified c-Myc TF as a negative regulator of lytic cycle replication [11]. Our previous global transcriptomic analysis revealed that E2F1 expression is transcriptionally activated during EBV latent infection in naïve B-lymphocytes but suppressed during reactivation into lytic-cycle replication [4,18]. E2F1, the first member of E2F family TFs, plays a central role in cell cycle progression, particularly the transition from the G1 to S phase, as well as DNA-damage response and apoptosis [19]. Accumulating evidence indicates that E2F1 regulates replication of several DNA viruses [20,21]. E2F1 functions are implicated in EBV pathogenesis, particularly in the context of latent infection [22–24]. However, its role during EBV lytic replication remains less well understood. Notably, the available evidence is contradictory, one study reports that BZLF1 can induce E2F1 expression in primary keratinocytes and gastric carcinoma cells [25], whereas another suggests that E2F1, in conjunction with c-Myc, suppresses BZLF1-mediated transactivation via a negative regulatory element located within the N-terminal region of BZLF1 [26]. Given that E2F activity is often deregulated by infection with DNA viruses including EBV, we hypothesize that E2F1 contributes to EBV induced B-cell lymphomagenesis by regulating its latent-to-lytic switch. Overall our data support a model in which E2F1 transcriptionally controls both cellular (c-Myc) and viral (BZLF1) gene expressions to suppress EBV lytic replication.

## Results

### EBV reactivation to lytic cycle replication suppresses E2F1 expression

The E2F family of TFs is crucial for regulating cell cycle progression, DNA replication and apoptosis [19,27,28]. In mammalian cells, there are eight E2F genes, each has distinct functions in cell fate. The E2F TFs can be categorized as transcription activators (E2F1-3), canonical repressors (E2F4-6) and non-canonical repressors (E2F7-8). Among the E2F family members, elevated E2F1 expression is typically implicated with the poor prognosis of several solid cancers. Accumulating evidence also suggests a direct correlation of E2F1 activities on EBV induced B-cell lymphomagenesis [22–24]. Reanalysis of our previous RNA sequencing (RNA-Seq) data (GSE235941) [4] of EBV latently infected naïve B-lymphocytes (0–4 days post-infection, dpi) as well as microarray data of EBV infected BL line BL31 [29] revealed significant transcriptional activation of E2F1 along with three other E2F members E2F2, E2F7 and E2F8 (S1A and S1B Fig). A similar trend of transcriptional activation of E2F1 was also witnessed in reanalysis of RNA-Seq data (GSE125974) and qRT-PCR of EBV induced B-cell transformation (0–28 dpi) (S1C and S1D Fig). RNA-Seq data of ‘Genotype-Tissue Expression (GTEx) portal and qRT-PCR analysis further substantiated E2F1 transcriptional activation in EBV transformed lymphoblastoid cell lines (LCLs) (S1E and S1F Fig).

In contrast to latent infection, there are contradictory reports available on E2F1 regulations during EBV lytic replication [25,26]. Elevated expressions of specific set of E2F genes during EBV latent infection prompted us to further investigate expression pattern of these E2F members during lytic cycle reactivation. Various small molecules have been identified as stimulators of EBV lytic cycle replication [14]. Combination of 12-O-tetradecanoylphorbol-13-acetate and sodium butyrate (TPA-NaBu) are being largely utilized in laboratory settings to induce EBV reactivation into lytic cycle replication from EBV positive B-cells. Recently our lab has also demonstrated that proteasomal inhibition promotes EBV lytic cycle replication [18]. Reanalysis of our previous RNA-Seq data of both TPA-NaBu (GSE237484) [4] and proteasomal inhibition by MG132 [18] induced EBV lytic cycle reactivation in LCLs demonstrated significant transcriptional repression of these specific E2F genes – E2F1, E2F2, E2F7 and E2F8 (S1G and S1H Fig).

**Fig 1:**
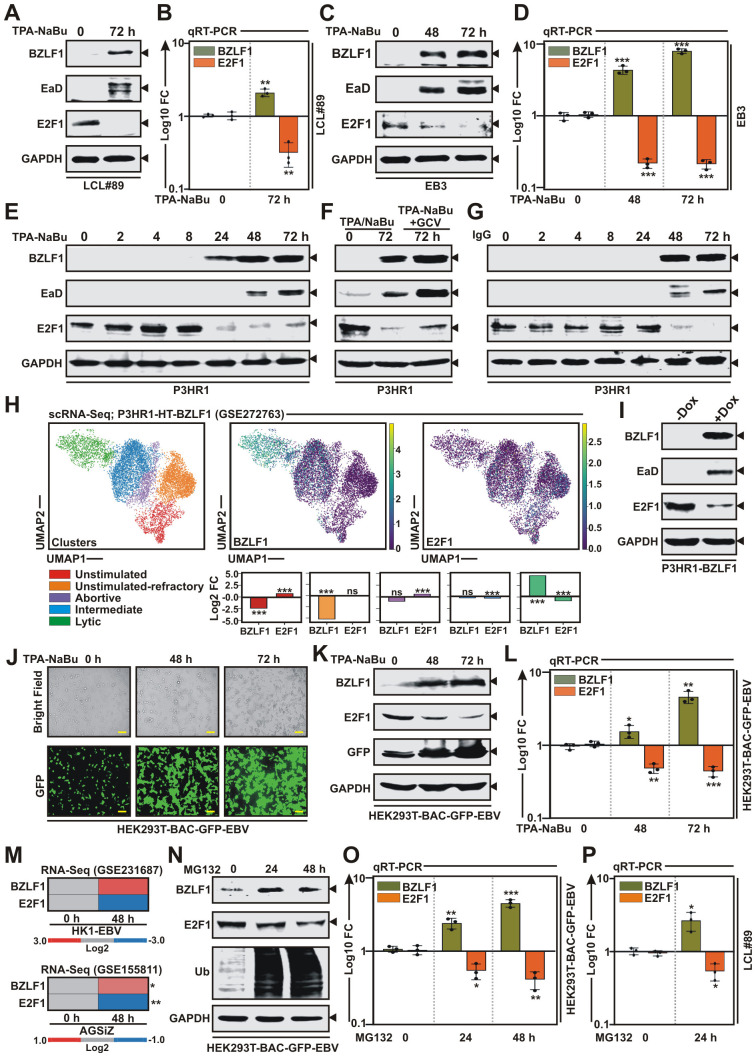
EBV lytic reactivation in both B- and epithelial cells downregulates E2F1 expression. (A) EBV lytic cycle replication was induced by with 20 ng/ml 12-O-tetradecanoylphorbol-13-acetate (TPA) and 3 mM sodium butyrate (NaBu) treatment in EBV transformed B-lymphocytes LCL#89 and subjected to Immunoblot analysis. (B) qRT-PCR analysis was performed on cDNA isolated from TPA-NaBu treated LCL#89 cells. (C) Immunoblot analysis of whole cell lysates (WCLs) from EBV^+^ EB3 cells treated with combination of TPA and NaBu for lytic reactivation induction for the indicated time points (0–72 h). (D) qRT-PCR analysis was performed on cDNA isolated from TPA-NaBu treated EBV^+^ EB3 cells for the indicated time points (0–72 h). (E) EBV lytic cycle replication was induced by TPA-NaBu treatment in EBV^+^ BL line P3HR1 for the indicated time points (0–72 h) and subjected to immunoblot analysis. (F) Immunoblot analysis of WCLs from P3HR1 cells treated with TPA-NaBu for lytic reactivation induction for 72 h in the presence and absence of 25 µg/ml ganciclovir (GCV). (G) EBV lytic cycle replication was induced in P3HR1 with 10 µg/ml anti-human IgG for the indicated time points (0–72 h) and subjected for immunoblot analysis. (H) Reanalysis of scRNA-Seq data (GSE272763) of P3HR1-ZHT cells (n = 8965) undergoing EBV lytic reactivation. Cell clusters were identified using unsupervised clustering and visualised using UMAP expression profiles (top panel) and bar plots (bottom panel) showing differential gene expression of BZLF1 and E2F1 across different clusters. (I) P3HR1 cells stably expressing BZLF1 under doxycycline responsive promoter were subjected to immunoblot analysis without or with doxycycline (-/ + DOX) treatment for 48 h. (J) Representative pictures of bright field and GFP fluorescence of TPA-NaBu treated HEK293T-BAC-GFP-EBV cells. Scale bars, 100 μm. (K) EBV lytic cycle replication was induced by TPA-NaBu treatment in HEK293T-BAC-GFP-EBV cells for the indicated time points (0–72 h) and subjected to immunoblot analysis. (L) qRT-PCR analysis was performed on cDNA from TPA-NaBu treated HEK293T-BAC-GFP-EBV cells. (M) Heat map representation of differential gene expression of BZLF1 and E2F1 from two bulk RNA-Seq datasets (GSE231687 and GSE155811) for EBV lytic reactivation in nasopharyngeal and gastric carcinoma cell lines, HK1-EBV and AGSiZ, respectively. (N) EBV lytic cycle replication was induced by 1 μM MG132 treatment in HEK293T-BAC-GFP-EBV cells for the indicated time points (0–48 h) and subjected to immunoblot analysis. (O) qRT-PCR analysis was performed on cDNA from 1 μM MG132 treated HEK293T-BAC-GFP-EBV cells for the indicated time points (0–48 h). (P) qRT-PCR analysis was performed on cDNA from 1 μM MG132 treated LCL#89. Immunoblots are representative of n = 3 replicates. For qRT-PCR analysis, the relative changes in transcripts of the selected genes were quantified using the 2^−ΔΔCT^ method and normalized with B2M as internal control. The results are presented as the mean ± SD, n = 3 biological replicates. Statistical significance was determined by a two-sided Student’s t-test, *P < 0.05; **P < 0.01; ***P < 0.001; ns, not significant.

Based on its impact on cell cycle and apoptosis as well as its well-known oncogenic properties [27,28], we further focused our study unequivocally on E2F1. In order to rule out inconsistencies due to chemically induced reactivation of EBV lytic cycle replication, several methods of lytic cycle induction was opted in multiple EBV+ cells (Fig 1). Expressions of EBV encoded IE gene BZLF1 and E gene EaD/BMRF1 by immunoblot and qRT-PCR analysis were employed to determine lytic cycle reactivation (Fig 1). As similar to RNA-Seq data, TPA-NaBu treatment mediated EBV reactivation led to significant downregulation of E2F1 expressions in LCLs (Fig 1A and 1B) and patient-derived BL lines - EB3 (Fig 1C and 1D) and P3HR1 (Fig 1E and 1F). Combination of TPA and NaBu induced EBV lytic cycle as early as 24 h and attained maximum levels between 48–72 h, as demonstrated by BZLF1 expression in immunoblot analysis (Fig 1E). Inhibition of lytic replication by ganciclovir (GCV) in TPA-NaBu-treated P3HR1 cells partially restored E2F1 expression, further substantiating that E2F1 is specifically downregulated upon induction of the EBV lytic cycle (Fig 1F). Of the several methods used in the laboratory settings for EBV lytic cycle induction, BCR activation represents the most physiologic [30]. Antigen recognition by the BCR can be recapitulated *in vitro* by cross-linking of surface immunoglobulins - IgG or IgM, depending upon which immunoglobulin is produced by the B-cell line [30]. Anti-IgG treatment of P3HR1 cells induced viral lytic cycle as early as 48 h and reached maximum levels at 72 h post-treatment resulted in distinct E2F1 depletion (Fig 1G). Similar to TPA-NaBu treatment, IgG crosslinking results also evidently demonstrated that initiation of EBV lytic cycle replication negatively regulates E2F1 expression levels in EBV + B-lymphocytes (Fig 1A-1E and 1G).

To further strengthen the impact of EBV lytic reactivation on E2F1 expression, we reanalysed single-cell RNA sequencing (scRNA-Seq) data (GSE272763) [31] from P3HR1-ZHT cells, which constitutively express BZLF1 fused to a modified hormone-binding domain of the 4-hydroxytamoxifen (4HT) receptor, enabling EBV lytic cycle induction upon 4HT treatment (Fig 1H). Consistent with previous findings, UMAP analysis of cells collected across an integrated time course (0–72 h post-4HT treatment) revealed five distinct cellular clusters, namely unstimulated, unstimulated-refractory, abortive, intermediate and lytic reactivated, with specific cellular and viral gene signatures (Fig 1H). Projection of BZLF1 and E2F1 expressions onto the UMAP space revealed that cells within the lytic cluster exhibited significantly reduced E2F1 and elevated BZLF1 expressions (Fig 1H). In contrast, cells within the unstimulated cluster demonstrated significantly higher E2F1 and lower BZLF1 expression levels (Fig 1H). Likewise, we generated a P3HR1 cell line stably expressing BZLF1 under the control of a doxycycline-inducible promoter (Fig 1I). 48 h post-doxycycline treatment, these cells showed robust induction of the viral lytic cycle, as evidenced by increased expression of BZLF1 and EaD (Fig 1I). In contrast, doxycycline treatment had no effect on BZLF1 expression or viral reactivation in the parental P3HR1 cells, confirming the specificity of the inducible system (S2 Fig). Doxycycline-induced BZLF1 expression and subsequent lytic reactivation led to a substantial decrease in E2F1 protein levels (Fig 1I), further supporting a negative regulatory effect of EBV lytic replication on E2F1 expression.

To understand whether EBV lytic replication in epithelial background also equally represses E2F1 expression, we first utilized HEK293T cells stably harbouring GFP-tagged EBV bacmid (Fig 1J-1L). Parallel to EBV positive B-lymphocytes, TPA-NaBu treatment transcriptionally suppressed E2F1 expression during viral lytic cycle reactivation (Fig 1J-1L). Additionally, we reanalysed two independent RNA-Seq datasets (GSE231687, GSE155811) for EBV lytic reactivation in nasopharyngeal and gastric carcinoma cell lines, HK1-EBV and AGSiZ, respectively (Fig 1M). HK1-EBV is a nasopharyngeal carcinoma line infected *in vitro* with a recombinant EBV Akata strain, while AGSiZ cell line was derived from a gastric carcinoma, which was infected with a recombinant EBV strain Akata BX-1. HK1-EBV cells were cultured at the air-liquid interface in order to induce EBV lytic reactivation. Since AGSiZ line was stably transduced with an inducible BZLF1 gene under doxycycline responsive promoter, upon doxycycline treatment EBV underwent robust lytic replication. In both models, bulk RNA-Seq analysis revealed a significant downregulation of E2F1 expression during EBV lytic cycle activation (Fig 1M). In addition, proteasomal inhibition by MG132 treatment also exhibited similar transcriptional repression in both HEK293T cells harbouring GFP-EBV bacmid (Fig 1N-1O) and LCL#89 (Fig 1P). Notably, neither TPA-NaBu nor MG132 treatment affected E2F1 expression in parental HEK293T cells (S3 Fig), further supporting the specificity of chemically induced viral lytic replication and its impact on E2F1 expression. Overall, these results evidently demonstrated significant transcriptional repression of E2F1 in response to EBV lytic cycle reactivation in both epithelial and B-cell background, raising the question of whether EBV adjusts E2F1 expression in order to sensitize latently infected cells for lytic cycle induction.

### EBV immediate early protein BZLF1 transcriptionally represses E2F1 expression

To understand the underlying molecular mechanism governing E2F1 transcriptional activation during EBV driven B-cell transformation or virally transformed B-lymphocytes, we first reanalyzed chromatin immunoprecipitation followed by deep sequencing (ChIP-Seq) data for all six EBV nuclear antigens (EBNAs) - EBNA1 (GSE73887), EBNA2 (GSE29498), EBNALP (GSE49338), and three EBNA3 genes (GSE88729) - EBNA3A, EBNA3B and EBNA3C in LCLs (S4 Fig and S1 Table). Additionally, since EBV encoded major latent membrane protein, LMP1 constitutively activates NF-κB signalling to promote LCLs growth and survival [32], we included ChIP-Seq data (GSE55105) for all NF-κB subunits RelA, RelB, cRel, p50, and p52 in our analysis (S4 Fig and S1 Table). Reanalysis of ChIP-Seq data revealed no viral genes along with NF-κB subunits were enriched in the E2F1 promoter region (S4 Fig).

**Fig 2:**
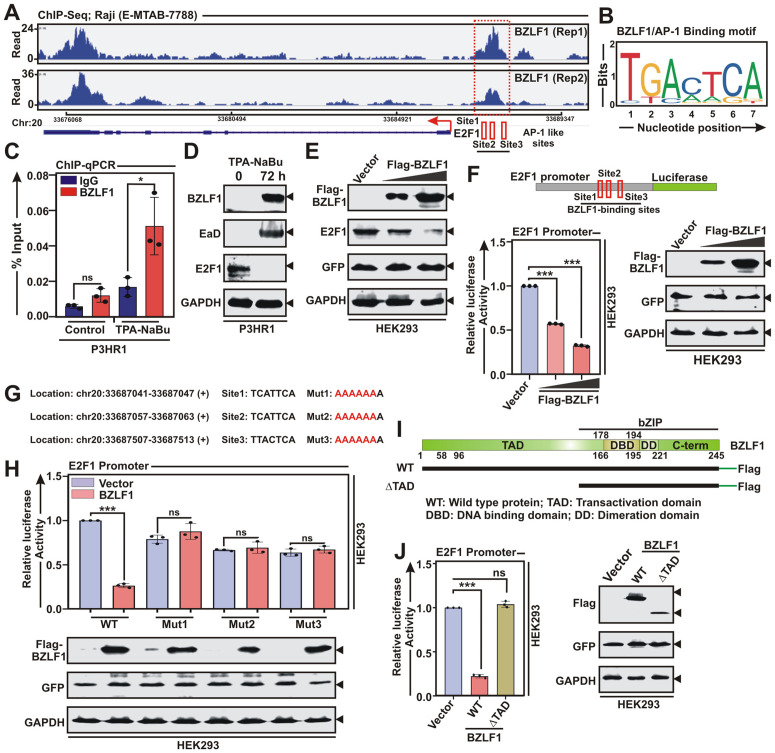
BZLF1 transcriptionally represses E2F1 expression. (A) Reanalysis of ChIP-Seq data (E-MTAB-7788) showing enrichment of BZLF1 on E2F1 promoter. Bottom panel indicates the MACS2 identified peaks (Sites 1–3) for BZLF1/AP-1 binding on E2F1 promoter. (B) BZLF1 homologue AP-1 binding motif identified on the MACS2 peaks of E2F1 promoter region. (C) ChIP-qPCR data showing recruitment of BZLF1 on E2F1 promoter upon EBV lytic cycle reactivation by TPA-NaBu treatment for 72 h in P3HR1 cells. (D) Immunoblot analysis of whole cell lysates (WCLs) from EBV^+^ P3HR1 cells reactivated to lytic cycle replication as similar to (C). (E) HEK293 cells transiently transfected with empty vector or increasing concentrations of flag-tagged BZLF1 expression plasmid for 36 h were harvested and subjected to immunoblot analysis. (F) Luciferase reporter activity and the corresponding immunoblot analysis of the wild-type E2F1 promoter in the presence of increasing concentrations of BZLF1 expression plasmid in transiently transfected HEK293 cells. (G) Schema showing three wild-type BZLF1/AP-1 binding sites (Sites 1–3) and their corresponding mutations (Muts 1–3) on E2F1 promoter for cloning into pGL3 luciferase reporter vector. (H) Luciferase reporter activity of the wild-type and the mutant E2F1 promoters in the presence of either vector control or BZLF1 expression plasmid in HEK293 cells. A fraction of the total protein was evaluated by immunoblot analysis. (I) Schema showing different structural domains of BZLF1 for cloning in flag-tagged expression vector. (J) Luciferase reporter activity and the corresponding immunoblot analysis of the E2F1 promoter in the presence of empty vector, wild-type (WT) or transactivation domain deleted (ΔTAD) BZLF1 expression plasmids in HEK293 cells. The results are presented as the mean ± SD, n = 3 biological replicates. Statistical significance was determined by a two-sided Student’s t-test, *P < 0.05; **P < 0.01; ***P < 0.001; ns, not significant.

Non-occupancy of latent viral oncoproteins coupled with transcriptional repression during lytic cycle reactivation prompted us to further investigate the binding capacity of EBV lytic proteins on E2F1 promoter region. BZLF1 belongs to the family of basic leucine zipper (bZIP) TFs, may solely induce EBV lytic cycle replication [12]_._ In addition to regulating viral lytic gene transcriptions, BZLF1 can also transcriptionally regulate expressions of multiple cell-cycle genes [25]. Moreover, there are two contradictory reports demonstrating while BZLF1 can induce E2F1 expression in primary keratinocytes and gastric carcinoma cells [25], E2F1 along with c-Myc obstruct BZLF1 mediated transactivation through a negative regulatory element located at the N-terminal region [26]. Reanalysis of ChIP-Seq data (E-MTAB-7788) [9] of Raji cells stably expressing BZLF1 under doxycycline responsive promoter revealed several distinct BZLF1 ChIP-Seq signals in the E2F1 promoter region (Fig 2A). Model-based analysis of ChIP-Seq (MACS2) tool for peak-calling with a *p*-value cut off set at p < 0.05 up to ~4 Kb upstream of the transcription start site (TSS) identified significant BZLF1 peak on the E2F1 promoter region (Fig 2A). BZLF1 is related to cellular AP-1 (activating protein 1) family of TFs that binds to two different classes of BZLF1-responsive elements on DNA - the canonical AP-1 binding site (TGACTCA) and methylated CpG containing motifs [33]. The identified ChIP-Seq signal on E2F1 promoter were assessed for BZLF1/AP-1 binding motifs using JASPAR tool and subsequently revealed three AP-1 motifs (Fig 2A and 2B). BZLF1 occupancy on E2F1 promoter was validated by ChIP-qPCR using P3HR1 cells with or without EBV lytic cycle induction (Fig 2C). As compared to the control there was a significant enrichment of BZLF1 binding onto E2F1 promoter region upon lytic cycle reactivation (Fig 2C). Lytic cycle reactivation in P3HR1 cells using a similar experimental set up also resulted in significant downregulation of E2F1 expression at protein level (Fig 2D).

Next, we wanted to check whether BZLF1 induction during lytic cycle replication is directly accountable for E2F1 transcriptional repression. HEK293 cells transiently transfected with flag-tagged BZLF1 expression vector demonstrated that BZLF1 expression was inversely correlated with the endogenous E2F1 expression in a dose dependent manner (Fig 2E). However, in contrast to BZLF1, the other IE protein, BRLF1, failed to repress E2F1 under similar experimental conditions (S5 Fig). Additionally, to rule out BZLF1 mediated post-translational activities, HEK293 cells were further transiently transfected with myc-tagged BZLF1 with or without flag-tagged E2F1 expression vectors and subjected to immunoblot and co-immunoprecipitaion analysis (S6A and S6B Fig). In contrast to endogenous E2F1, BZLF1 had effect no effect on exogenously expressed flag-E2F1 (S6A Fig), nor these two proteins interacted with each other (S6B Fig), suggesting the possibility of BZLF1 mediated transcriptional repression of E2F1 in EBV positive cells during lytic cycle replication. BZLF1 mediated depletion of E2F1 expression was further assessed by luciferase based promoter assays in both epithelial (HEK293) and B-lymphocyte (DG75) models (Figs 2F and S7A, respectively). E2F1 promoter region comprising of three BZLF1/AP-1 binding sites were inserted upstream of the luciferase gene in pGL3-basic vector (Fig 2F). BZLF1 significantly repressed E2F1 promoter activity in a dose dependent manner in both experimental settings (Figs 2F and S7A). Upon mutation of all three BZLF1/AP-1 sites on the E2F1 promoter region, BZLF1 failed to repress E2F1 transcription (Fig 2G and 2H), further validating BZLF1 mediated transcriptional suppression of E2F1. We and others have previously shown that while the N-terminal transactivation domain (TAD) of BZLF1 regulates gene transcription, its C-terminal basic leucine-zipper (bZIP) domain retains DNA-binding activity comparable to that of the wild-type (WT) protein [4,12]. To identify the specific domains of BZLF1 responsible for binding the E2F1 promoter, we generated ΔTAD and ΔbZIP constructs expressing flag-tagged BZLF1 proteins lacking the TAD and bZIP regions, respectively (S8A-S8B Fig). Consistent with previous reports [4,12], ChIP-qPCR assays in HEK293 cells transiently transfected with either vector control or flag-tagged BZLF1 constructs revealed significant enrichment of WT and ΔTAD BZLF1 proteins at the E2F1 promoter (S8C Fig), highlighting the essential role of the bZIP domain in DNA binding. To assess BZLF1-mediated repression of E2F1 transcription, we employed both WT and ΔTAD BZLF1 constructs, each retaining DNA-binding capacity, in E2F1 promoter assays (Fig 2I-2J). The results demonstrated that, unlike WT BZLF1, the ΔTAD mutant failed to repress E2F1 promoter activity (Fig 2I-2J). Together, these findings support a model in which, upon lytic cycle induction, EBV expresses its IE protein BZLF1, which subsequently represses E2F1 transcription via its transactivation domain.

### E2F1 directly binds and differentially regulates EBV latent and lytic promoters

**Fig 3:**
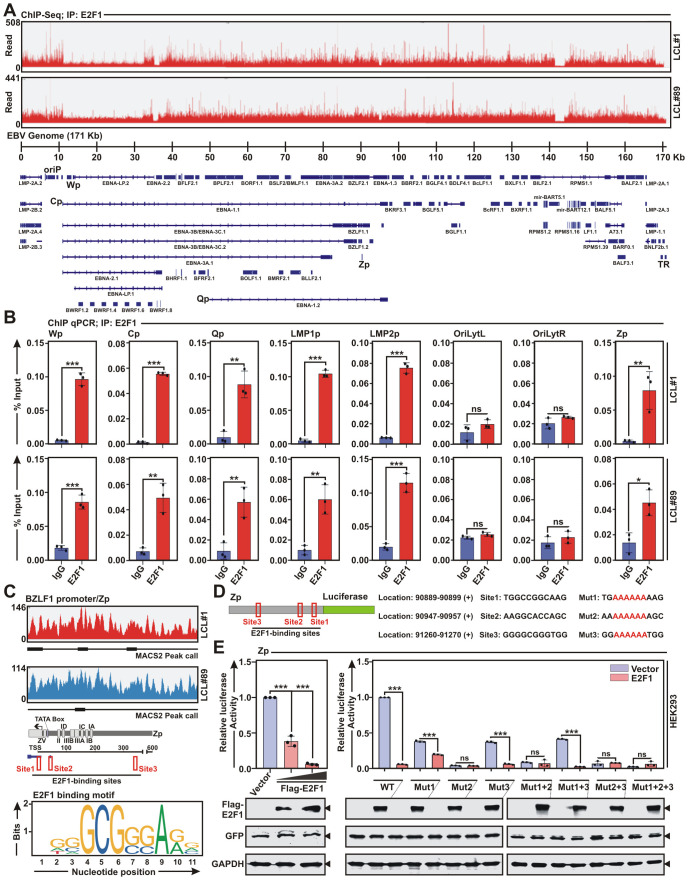
E2F1 directly targets EBV genome to differentially control both latent and lytic transcriptional programs. (A) ChIP-Seq analysis of E2F1 binding in two EBV transformed lymphoblastoid cell lines – LCL#1 and LCL#89. Tracks are aligned with the annotated EBV genome shown at the bottom. (B) ChIP-qPCR analysis of E2F1 occupancy at different EBV promoters and genomic regions. Anti-E2F1 ChIP was performed on chromatin extracted from LCL#1 and LCL#89, followed by qPCR using primers specific for Wp, Cp, Qp, LMP1p, LMP2p, OriLytL, OriLytR and Zp regions. Data are presented as % input. (C) E2F1 ChIP-Seq tracks and the corresponding MACS2 identified peaks on BZLF1 promoter region (Zp) in LCL#1 and LCL#89. Bottom panel indicates different Zp elements and three putative E2F1 binding motifs obtained from JASPAR database in Zp. (D) Schema showing three wild-type E2F1 binding sites (Sites 1–3) and their corresponding mutations (Muts 1–3) on Zp for cloning into pGL3 luciferase reporter vector. (E) Luciferase reporter activity and the corresponding immunoblot analysis of the wild-type (WT) and mutant (Mut) Zp in the absence and presence of flag-tagged E2F1 expression vector in HEK293 cells. The results are presented as the mean ± SD, n = 3 biological replicates. Statistical significance was determined by a two-sided Student’s t-test, *P < 0.05; **P < 0.01; ***P < 0.001; ns, not significant.

Given its role as a robust DNA-binding transcription factor and the observed differential expressions between EBV latent infection and lytic cycle reactivation, we hypothesized that E2F1 directly interacts with the EBV genome to regulate viral gene transcription. We employed ChIP-Seq to assess E2F1 binding across the EBV genome in two LCLs (Fig 3A). E2F1 occupancy was observed near all the EBV latent promoters – Wp, Cp, Qp, LMP1p and LMP2p, as confirmed by ChIP-qPCR analysis in both LCLs (Fig 3B). Within the lytic replication genomic regions significant E2F1 occupancy was only observed at the BZLF1 promoter/Zp (Fig 3B). However, no E2F1 enrichment was observed near both OriLyt regions – OriLytL and OriLytR (Fig 3B). BZLF1 directly binds to the OriLyt region and initiate EBV lytic genome replication [11]. Recently, c-Myc was shown to directly bind to OriLyt region, thereby controlling BZLF1 occupancy as well as EBV latent-to-lytic switch [11]. To further pinpoint the putative binding sites of E2F1 within the proximal genomic region to the ChIP-Seq signals, JASPAR web tool [34] was utilized. We identified multiple putative E2F1 binding motifs near all the viral promoters including Zp (Fig 3C and S2 Table). We independently cloned these EBV genomic regions in pGL3-basic vector and performed promoter assays in the absence and presence of E2F1 (Figs 3D, 3E and S9). Among the latent promoters, varied E2F1 regulations were observed (S9 Fig). While E2F1 transcriptionally repressed Wp, Cp and both LMP1p and LMP2p activities, positive transcriptional regulation was observed for Qp (S9 Fig).

Notably, E2F1 repressed Zp activity in a dose-dependent manner in both epithelial (HEK293) and B-cell (DG75) models (Figs 3E and S7B). The Zp region [EBV genome coordinates NC_007605.1: 90853–91482 (+)] contains three distinct E2F1 binding sites, including site 1 (TGGCCGGCAAG), site 2 (AAGGCACCAGC) and site 3 (GGGGCGGGTGG) (Fig 3C-3D). Several known cis-regulatory elements ZI–ZD, ZII, ZIIIA–B and ZV essential for basal and inducible promoter activity are located within the −221 to +12 region relative to the BZLF1 TSS [35] (Fig 3C). Site 1 overlaps the TSS, site 2 lies near the TATA box between the ZV and ZII elements, and site 3 is positioned distally at approximately −418 relative to the TSS (Fig 3C). To evaluate the functional relevance of these binding sites, the −618 to +12 region of Zp, encompassing all three E2F1 binding sites and the corresponding mutant versions were cloned upstream of a luciferase reporter gene in the pGL3-basic vector and subjected for luciferase based promoter assay in the absence and presence of a flag-tagged E2F1 expression construct (Fig 3D-3E). Combinatorial mutational analysis revealed that sites 1 and 2 are both critical for E2F1-mediated repression of BZLF1 promoter activity. Strikingly, mutation of site 2 alone resulted in a complete loss of luciferase signal (Fig 3E). We hypothesize that this mutation may disrupt the assembly of the transcription pre-initiation complex, thereby impairing recruitment of RNA polymerase II and subsequent transcriptional initiation.

### E2F1 expression controls EBV lytic cycle reactivation

**Fig 4:**
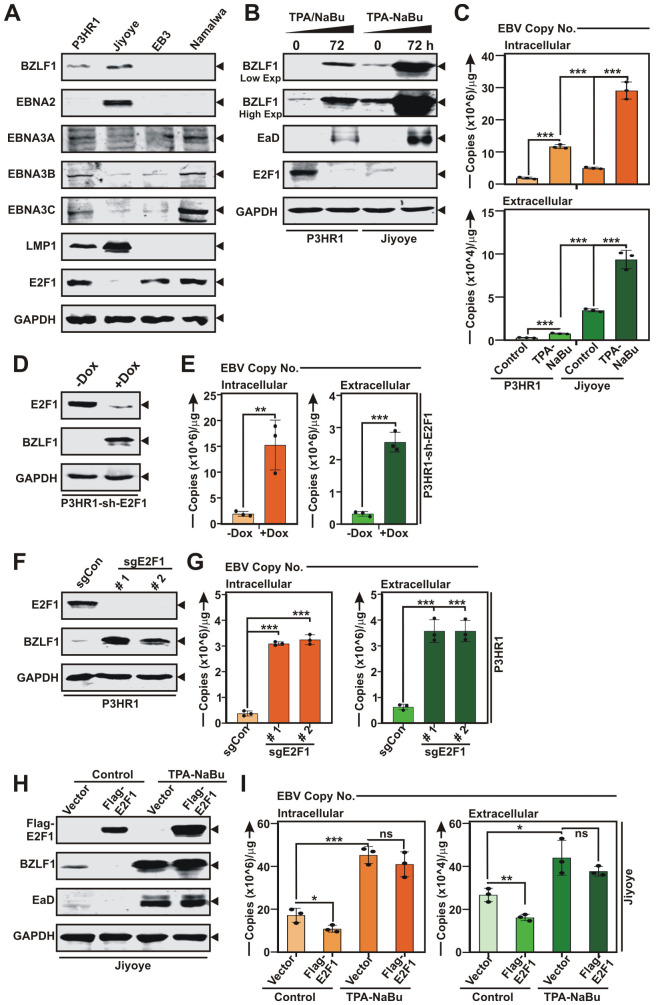
E2F1 suppresses EBV lytic cycle reactivation. (A) Immunoblot analysis of whole cell extracts of four EBV^+^ BL lines – P3HR1, Jiyoye, EB3 and Namalwa with the indicated antibodies against viral and cellular proteins. (B) P3HR1 and Jiyoye cells were reactivated to lytic cycle replication by TPA-NaBu treatment for 72 h were subjected to immunoblot analysis. (C) EBV intracellular or DNase-treated extracellular genome copy number was quantified by qRT-PCR from P3HR1 and Jiyoye cells treated with TPA-NaBu for 72h. (D) P3HR1 cells stably expressing sh-RNA specific for E2F1 under doxycycline responsive promoter were subjected to immunoblot analysis without or with doxycycline (-/ + DOX) treatment. (E) qRT-PCR of EBV intracellular or extracellular genome copy number from P3HR1-sh-E2F1 cells without or with doxycycline (-/ + DOX) treatment. (F) Immunoblot analysis of P3HR1 cells expressing either control (sgCon) or two E2F1 specific sgRNAs. (G) qRT-PCR of EBV intracellular or extracellular genome copy number quantified from P3HR1 cells in the presence of control or two E2F1-specific sgRNAs. (H) Jiyoye cells transiently transfected either control vector or flag-tagged E2F1 expression plasmid, followed by TPA-NaBu treatment for 72 h were subjected to immunoblot analysis. (I) EBV intracellular or DNase-treated extracellular genome copy number analysis was performed in Jiyoye cells transiently transfected either control vector or flag-tagged E2F1 expression plasmid, followed by TPA-NaBu treatment for 72 h. The results are presented as the mean ± SD, n = 3 biological replicates. Statistical significance was determined by a two-sided Student’s t-test, *P < 0.05; **P < 0.01; ***P < 0.001; ns, not significant.

Hitherto, our data demonstrated that EBV lytic cycle transactivator BZLF1 and E2F1 negatively cross-regulate each other to maintain either latent or lytic replication status. We hypothesized that E2F1 expression might control EBV latent-to-lytic switch and lessening E2F1 levels could promote viral lytic replication. We first checked E2F1 expressions in several EBV positive BL lines – P3HR1, Jiyoye, EB3 and Namalwa (Fig 4A). While P3HR1, EB3 and Namalwa had significant E2F1 expressions, Jiyoye exhibited little or negligible E2F1 expression (Fig 4A). To investigate the role of E2F1 in regulating EBV lytic cycle replication, we further utilized P3HR1 and Jiyoye cells with distinct expression pattern of E2F1. Notably, P3HR1 containing type 2 EBV DNA is a clonally derived subline of Jijoye cells in which the EBNA2 region has been deleted, making them suitable for comparison in experimental setups. Upon lytic cycle reactivation, Jiyoye with little or no E2F1 expression significantly enhanced viral lytic cycle replication as compared to P3HR1 with elevated E2F1 expression (Fig 4B-4C). The results were evaluated by immunoblot analysis of viral lytic gene expressions – BZLF1 and BMRF1/EaD (Fig 4B) as well as by quantifying both intracellular and extracellular EBV genome copy number (Fig 4C).

In order to further confirm the phenomenon, P3HR1 cells were either knockdown (KD) for E2F1 using specific sh-RNA under doxycycline responsive promoter or knockout (KO) using two sets of sgRNAs (Figs 4D-4G and S10). The efficiency of KD/KO was validated using immunoblot analysis (Fig 4D and 4F). Intriguingly, either KD or KO of E2F1 markedly enhanced BZLF1 expression (Fig 4D and 4F) and virion production (Fig 4E and 4G) even without lytic cycle induction. To validate this notion further, P3HR1 cells stably expressed sh-RNA for E2F1 were either left untreated or subjected to viral lytic cycle induction by TPA-NaBu treatment in the absence or presence of doxycycline for 72 h (S10A-S10B Fig). While E2F1 KD or TPA-NaBu treatment alone enhanced BZLF1 transactivation and subsequent virion production, combination of both E2F1 KD and chemical lytic cycle inducer further enhanced the process (S10A-S10B Fig). In contrast, ectopic expression of flag-tagged E2F1 expressing construct in Jiyoye cells significantly blocked BZLF1 expression and mature virion production (Fig 4H-4I). Overall, these results suggest that E2F1 specifically acts at the level of BZLF1 to govern EBV lytic cycle replication.

### BZLF1-E2F1 cross-regulations are cell cycle and pRb independent

In contrast to most of the DNA viruses, herpesviruses including EBV induce cell cycle arrest at G1 phase during lytic cycle replication in both epithelial and B-cell backgrounds [4,36]. Although the underlying mechanisms are not well defined, it is suggested that this quasi-G1/S state expedites viral lytic replication. Notably, multiple EBV lytic cycle members including IE proteins BZLF1 and BRLF1, E protein BORF2 and L protein BGLF2 cause cell cycle arrest at the G1 to S phase transition [36–39]. This lytic replication-mediated cell cycle arrest prompted further investigation into its impact on E2F1 expression. Toward this objective, in addition to lytic cycle reactivation by TPA-NaBu, P3HR1 cells were additionally treated with mimosine/leucenol, a tyrosine analogue that specifically arrests dividing cells in the late G1 phase by inhibiting DNA replication initiation (S11A-S11C Fig). In alignment with the previous findings, treatment with both mimosine and TPA-NaBu in P3HR1 cells exhibited characteristic G1/S cell cycle arrest (S11A-S11B Fig). However, in contrast to EBV lytic cycle reactivation by TPA-NaBu treatment, mimosine-induced G1 arrest had no repressive effect on E2F1 expression levels (S11C Fig).

While E2F1 is primarily associated with promoting cell cycle progression by transcriptional activation of genes essential for DNA replication and S-phase entry, it can also induce cell cycle arrest and apoptosis under various stress responses such as DNA damage signals [20–22]. The retinoblastoma protein (pRb) negatively regulates both E2F1-driven cell cycle and apoptosis through interaction with a pRb-binding motif located at the edge of the C-terminal transactivation domain (residues 409–426) [22]. Nevertheless, ectopic expression of flag-tagged both WT (residues 1–437) and the mutant (residues 1–400) E2F1, lacking the pRb binding domain (PBD), in Jiyoye cells resulted in drastic depletion of basal BZLF1 of expressions (S11D-S11E Fig), indicating that E2F1 mediated suppression of BZLF1 expression and subsequent viral lytic cycle reactivation is a pRb-independent phenomenon. Collectively, these results suggest that E2F1-BZLF1 cross-regulation is unaffected by cell-cycle arrest or its mediators.

### E2F1, but not E2F2, suppresses BZLF1 expression at the transcriptional level via its transactivation domain

**Fig 5:**
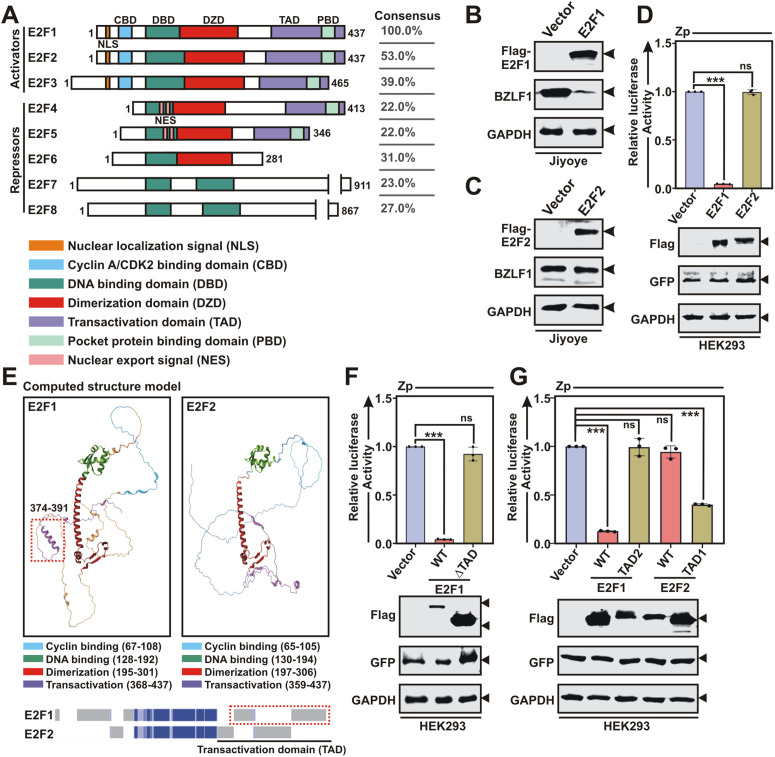
E2F1, but not E2F2, transcriptionally deactivates BZLF1 expression through its transactivation domain. (A) Schema showing known structural domains of all eight E2F genes (E2F1-8). Right panel indicates the % sequence similarities of E2F1 and other E2F family members. (B) Immunoblot analysis of Jiyoye cells transiently transfected with either control vector or flag-tagged E2F1 expression plasmid. (C) Immunoblot analysis of Jiyoye cells transiently transfected with either control vector or flag-tagged E2F2 expression plasmid. (D) Luciferase reporter activity and the corresponding immunoblot analysis of the BZLF1 promoter (Zp) in the presence of control vector, or flag-tagged E2F1 or E2F2 expression plasmids in HEK293 cells. (E) Pairwise 3D structure alignment of E2F1 and E2F2. Bottom panel indicates sequence similarly (Blue) and dissimilarity (Grey) between E2F1 and E2F2 structure alignment. (F) Luciferase reporter activity and the corresponding immunoblot analysis of the Zp in the presence of empty vector, wild-type (WT) or transactivation domain deleted (ΔTAD) E2F1 expression plasmids. (G) Luciferase reporter activity and the corresponding immunoblot analysis of the Zp in the presence of empty vector, WT E2F1, E2F1 fused with E2F2-TAD domain (TAD2), WT E2F2 or E2F2 fused with E2F1-TAD domain (TAD1) expression plasmids in HEK293 cells. The results are presented as the mean ± SD, n = 3 biological replicates. Statistical significance was determined by a two-sided Student’s t-test, *P < 0.05; **P < 0.01; ***P < 0.001; ns, not significant. ND, not detected.

The E2F TFs exerts their functions through specific DNA-binding and protein interacting domains (Fig 5A). Cell cycle activities of E2F genes are tightly regulated by pocket proteins (pRb, p107 and p130) dependent (E2F1-5) and independent (E2F6-8) manner [19]. Apoptotic regulations of the prototype member E2F1 sets it apart from other E2Fs [19]. Given both distinct and overlapping functions of E2F TFs, we next asked whether other E2F members can also suppress BZLF1 transactivation. Among the three activator family members E2F2 shared highest sequence homology (53%) with E2F1 (Fig 5A and S3 Table). Moreover, because in response to EBV lytic cycle induction, E2F1 and E2F2, but not E2F3, were significantly depleted at the transcriptional levels (S1G-S1H Fig), we reasoned that E2F2 might have similar repressive activity on BZLF1 expression. However, in contrast to E2F1, ectopic E2F2 expression failed to inhibit BZLF1 expression as well as its promoter activity (Fig 5B-5D). Computed structure model analysis demonstrated that although E2F1 and E2F2 share both significant primary sequence and three dimensional structure resemblance, exclusively within the DNA binding (DBD) and heterodimerization (DZD) domains, they display distinct features within the C-terminal transactivation domain (TAD) (Fig 5E). We therefore hypothesized that although E2F1 and E2F2 have similar DNA-binding abilities across the genome, owing to its specific TAD, E2F1 may display critical biological functions distinct from other E2Fs. Luciferase based promoter assay indeed revealed that WT (residues 1–437), but not, ΔTAD E2F1 (residues 1–358) lacking the TAD, failed to transcriptionally repress Zp activity (Fig 5F). To gain insights into E2F1 TAD negative regulations on BZLF1 expression, we swapped the respective TADs (residues 359–437) between E2F1 and E2F2 and generated constructs expressing chimeric proteins – E2F1-TAD2 (comprising E2F2 TAD) and E2F2-TAD1 (comprising E2F1 TAD). While contrary to WT E2F1, E2F1-TAD2 and WT E2F2 failed to transcriptionally repress Zp activity, E2F2 containing E2F1 specific TAD (E2F2-TAD1) significantly blocked Zp activity (Fig 5G). These data support a fundamental role of E2F1 TAD in transcriptional deactivation of BZLF1 during viral lytic replication.

### E2F1 expression restricts specific EBV latency program and lytic cycle reactivation

To further illustrate the effects of E2F1 on EBV gene expression, we performed RNA-Seq analysis of P3HR1 transcripts with or without E2F1 KD in the absence and presence of EBV lytic cycle induction following TPA-NaBu treatment (S12A Fig and S4 Table). The transcriptomic data were first aligned with EBV type 2 genome. Most EBV lytic genes were transcriptionally activated by either E2F1 KD or TPA-NaBu treatment, combination of both E2F1 KD and the chemical inducer further amplified the effect (S12A Fig and S4 Table). EBNA2 deleted P3HR1 usually displays atypical latency II consisting of both type-II (expressing LMP1 and LMP2A) and Wp-restricted (expressing EBNALP and EBNA3 genes - 3A/3B/3C) latency programs [40]. Among the latent genes, all three EBNA3 genes EBNA3A, EBNA3B and EBNA3C were transcriptionally activated upon E2F1 KD (S12A Fig and S4 Table), indicating E2F1 depletion enforces a more Wp-restricted latency in P3HR1 cells. Additionally, Cp mediates transcription of EBNA genes in type-III latency cells [41]. Consistent with this finding, luciferase based promoter assays demonstrated that while E2F1 expression significantly inhibited transcription from Wp and Cp, it facilitated transcription from Qp in a dose dependent manner (S9 Fig). During restricted latency (types I and II), the Qp promoter primarily drives the transcription of the EBNA1 gene. In contrast, the Cp and Wp promoters, which are active during type III latency to express all six EBNAs, remain silent during restricted latency [41]. Induction of EBV lytic and latent gene expressions in response to E2F1 depletion in P3HR1 cells was further validated by qRT-PCR analysis (S12B Fig). While additional roles in EBV latency maintenance are possible, collectively these data suggest that E2F1 depletion favours a more Wp-restricted type-II or Cp-restricted type-III EBV latency programs and sensitizes lytic cycle reactivation. In agreement to this, RNA-Seq analysis of Mutu I and Mutu III cells (GSE136597), expressing Qp-restricted type-I latency and Cp-restricted type-III latency, respectively, revealed significant transcriptional repression of E2F1 in Mutu III cells (S12C Fig).

### E2F1 acts prior to c-Myc for its positive regulation and thereby controls EBV latent-to-lytic switch

**Fig 6:**
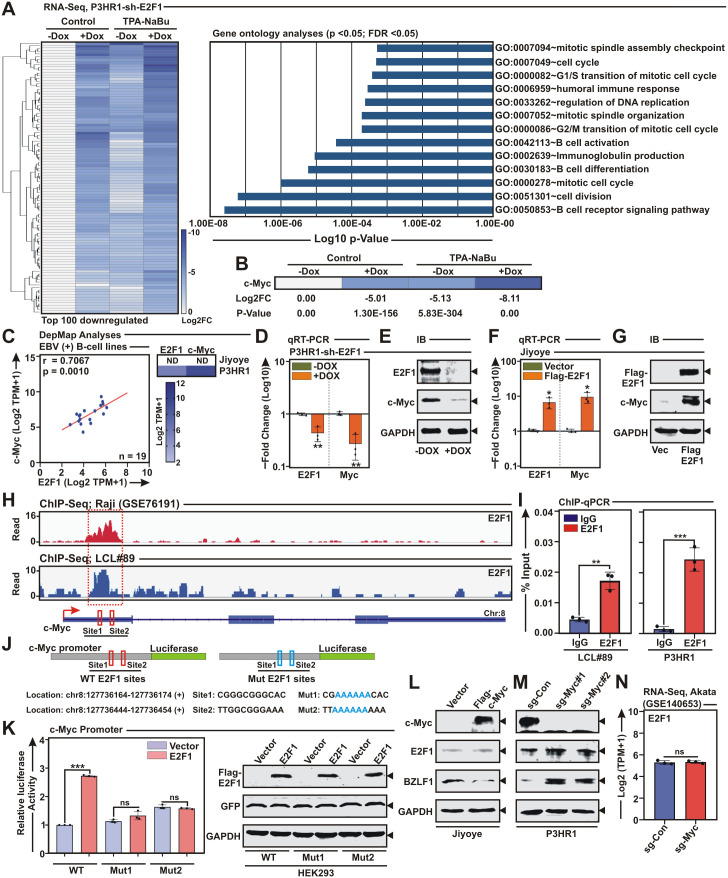
E2F1 positively regulates c-Myc expression. (A) Whole transcriptome analysis of P3HR1 stably expressing E2F1 sh-RNA (P3HR1-sh-E2F1) in the absence and presence of doxycycline (-/ + DOX) were either left untreated or treated with TPA/sodium butyrate (NaBu) for 72 h. Left panel indicates heatmap analysis of top 100 downregulated genes. Right panel bar diagram indicates most significantly affected pathways (*p* < 0.05, FDR < 0.05) based on top 100 downregulated genes in the RNA-Seq data of E2F1 knockdown and EBV lytic cycle reactivation. (B) Heat map visualization of c-Myc transcripts in the P3HR1 RNA-Seq data. (C) Two-tailed unpaired Student’s t-test and two-sided Pearson’s correlation were employed to analyse the association between E2F1 and c-Myc transcripts in 19 EBV^+^ BL lines from DepMap portal. Right panel indicates heatmap representation of E2F1 and c-Myc expressions in P3HR1 and Jiyoye cells. (D) qRT-PCR analysis of cDNA isolated from P3HR1-sh-E2F1 cells without or with doxycycline (-/ + DOX) treatment. (E) Immunoblot analysis of P3HR1-sh-E2F1 cells without or with doxycycline (-/ + DOX) treatment. (F) qRT-PCR analysis of cDNA isolated from Jiyoye cells transiently transfected with either control vector or flag-tagged E2F1 expression plasmid. (G) Immunoblot analysis of Jiyoye cells transiently transfected with either control vector or flag-tagged E2F1 expression plasmid. (H) EBV^+^ LCL#89 and Raji (GSE76191) ChIP-Seq tracks of E2F1 occupancy at c-Myc gene locus. (I) ChIP-qPCR analysis of E2F1 occupancy at c-Myc promoter region in LCL#89 and P3HR1 cells. (J) Schema showing two wild-type E2F1 binding sites (Red, Sites 1–2) and their corresponding mutations (Blue, Muts 1–2) on c-Myc promoter region for cloning into pGL3 luciferase reporter vector. (K) Luciferase reporter activity and the corresponding immunoblot analysis of the wild-type (WT) and mutant (Mut) c-Myc promoter in the absence and presence of flag-tagged E2F1 expression vector in HEK293 cells. (L) Immunoblot analysis of Jiyoye cells transiently transfected with either control vector or flag-tagged c-Myc expression plasmid. (M) Immunoblot analysis of P3HR1 cells expressing either control (sgCon) or two c-Myc specific sgRNAs. (N) Reanalysis of RNA-Seq data (GSE140653) of E2F1 transcripts in Akata cells expressing either control (sgCon) or c-Myc specific sgRNA. The results are presented as the mean ± SD, n = 3 biological replicates. Statistical significance was determined by a two-sided Student’s t-test, *P < 0.05; **P < 0.01; ***P < 0.001; ns, not significant.

To gain insights into E2F1 transcriptional network involved in EBV lytic cycle reactivation, we further analysed the P3HR1 cell transcripts in response to E2F1 KD with or without EBV lytic cycle induction (Fig 6A and S5 Table). As expected, global transcriptomics analysis of both E2F1 KD and EBV lytic cycle reactivation demonstrated significant downregulation of genes involved in cell pathways featuring both G1/S and G2/M transitions of the cell cycle, DNA replication, B-cell activation, B-cell differentiation and B-cell receptor signalling (Fig 6A and S5 Table). Among the most downregulated genes by E2F1 depletion and lytic cycle induction, c-Myc appeared within top 10 genes (Fig 6B and S5 Table). This result raised the question of whether c-Myc TF, which is strongly expressed during EBV infection of naïve B-lymphocytes and thereby aids to latency establishment [11], could be a direct target of E2F1. Moreover, a recent genome-wide CRISPR/Cas9 screening identified c-Myc linked transcriptional network necessary for suppression of EBV lytic cycle replication [11]. While accumulating research indicated seemingly paradoxical transcriptional regulations of these two important molecules in various cell types, particularly in solid cancers [42–45], there are no reports available in the context of EBV associated B-cell lymphomas. DepMap analysis of 19 EBV positive B-cell lines demonstrated that E2F1 and c-Myc transcripts were significantly correlated (r = 0.7067; p = 0.001) (Fig 6C). In contrast, no significant correlation was found in EBV negative B-cell lymphoma lines as well as multiple epithelial cancer lines including lung, breast, prostate and skin carcinomas in DepMap cell lines portal (S13A Fig). Analysis of TCGA patients’ samples data of EBV negative diffuse large B-cell lymphoma (DLBCL) and corresponding solid cancers - lung adenocarcinoma (LUAD), breast invasive carcinoma (BRCA), prostate adenocarcinoma (PRAD) and skin cutaneous melanoma (SKCM) further validated this notion (S13B Fig).

P3HR1 with elevated E2F1 expression displayed high level of c-Myc expression, while Jiyoye had insignificant expressions of both E2F1 and c-Myc (Fig 6C). We next asked whether E2F1 is directly responsible for c-Myc expression. Both qRT-PCR and immunoblot analysis of E2F1 depletion in P3HR1 and ectopic E2F1 expression in Jiyoye further confirmed the interdependence of E2F1 and c-Myc expressions in EBV positive B-cell lymphoma setting (Fig 6D-6G). To gain insights into the underlying mechanism governing E2F1-directed c-Myc transcription, we analysed/re-analyzed E2F1 ChIP-Seq data-sets in both LCLs and BL background (GSE76191) (Fig 6H). ChIP-Seq profiles indicated strong E2F1 enrichment in the first exon of the c-Myc gene locus (Fig 6H). E2F1 ChIP-Seq signals on c-Myc promoter/enhancer were further validated by ChIP-qPCR analysis in both LCLs and P3HR1 cells (Fig 6I). To further assess the E2F1-mediated transcriptional regulation of c-Myc, we performed luciferase reporter assay using the c-Myc exonic region spanning from 127736027 to 127736461 in chromosome 8 containing two putative E2F1 binding motifs (Fig 6J-6K). A concentration dependent increase in the c-Myc promoter driven luciferase activity was observed in HEK293 cells transfected with E2F1 expression vector (S14A Fig). While WT E2F1 significantly increased luciferase activity, mutant E2F1 with deleted TAD (E2F1 ΔTAD) failed to transactivate the c-Myc promoter/enhancer region (S14B Fig). Luciferase reporter assay with mutations in both E2F1-recognition sites within c-Myc locus further established E2F1 mediated positive transcriptional regulation of c-Myc (Fig 6J-6K). Together, these findings indicated that the E2F1 acts as a direct transcriptional activator of c-Myc.

Since c-Myc and E2F1 have been shown to activate each other’s transcription [46–48], we next investigated the impact of c-Myc on E2F1 transcription in EBV positive cells. In contrast to the effect of ectopic E2F1 expression on increased c-Myc expression in both Jiyoye and HEK293T cells harbouring EBV bacmid (Figs 6G and S14C), c-Myc expression failed to elevate endogenous E2F1 expression in these cells (Figs 6L and S14D). To further validate this data, two sgRNAs were designed to target c-Myc and subsequently checked their effect on E2F1 expression in P3HR1 cells (Fig 6M). In agreement with the ectopic expression settings, c-Myc depletion also had no effect on E2F1 expression (Fig 6M). RNA-Seq analysis (GSE140653) [11] of EBV positive Akata transcripts following expression of control or sgRNA targeting c-Myc further validated this notion (Fig 6N). Additionally, analysis of publicly available two ChIP-Seq datasets (GSE30399 and GSE36354) for c-Myc in LCLs revealed no distinct peaks in E2F1 gene locus (S14E Fig). In concordance with these data, no change in the luciferase activity was noted in HEK293 cells transfected with WT E2F1 promoter in the absence and presence of increasing doses of c-Myc expressing construct (S14F Fig), suggesting that E2F1 acts prior to c-Myc for its transcriptional activation.

**Fig 7:**
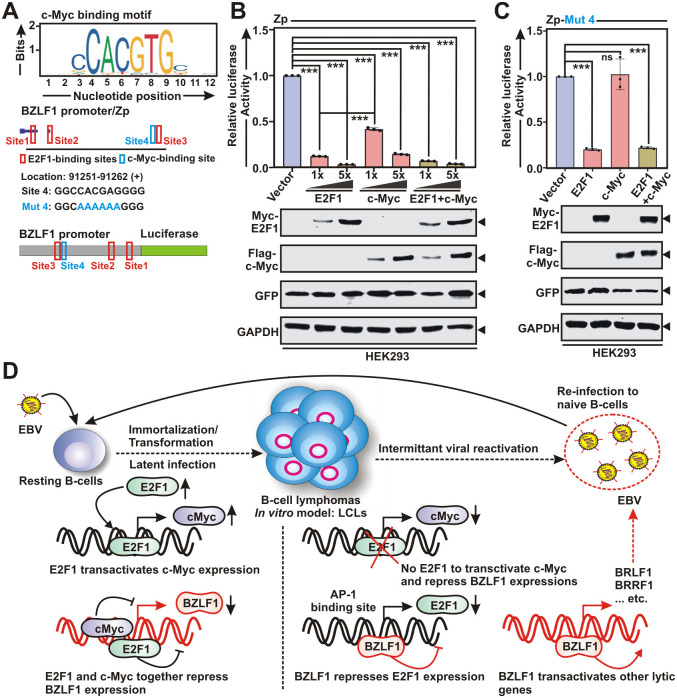
Together with c-Myc, E2F1 transcriptionally repress BZLF1 expression and EBV lytic cycle reactivation. (A) E2F1 (red, sites 1–3) and c-Myc (blue, site 4) binding motifs obtained from JASPAR database in BZLF1 promoter (Zp) region. Schema showing single wild-type c-Myc binding site (Site 4) and the corresponding mutation (Mut 4) on Zp for cloning into pGL3 luciferase reporter vector. (B) Luciferase reporter activity and the corresponding immunoblot analysis of the wild-type Zp in the absence and presence of E2F1 and c-Myc expression plasmids either in increasing concentrations or in combination in HEK293 cells. (C) Luciferase reporter activity and the respective immunoblot analysis of the mutant Zp in the absence and presence of E2F1 and c-Myc expression plasmids either in increasing concentrations or in combination in HEK293 cells. (D) Schematic model depicting E2F1’s role in repressing EBV lytic cycle reactivation. During EBV latency, E2F1 promotes c-Myc expression, together which bind to the BZLF1 promoter and repress its leaky expression that impedes subsequent expression of other lytic genes. Upon EBV lytic reactivation, BZLF1, on the contrary, transcriptionally repress E2F1 and thereby c-Myc expression, highlighting a unidirectional regulatory hierarchy. BZLF1 mediated E2F1 depletion deploys an effective lytic cycle environment that facilitates a cascade of EBV lytic gene expression. The results are presented as the mean ± SD, n = 3 biological replicates. Statistical significance was determined by a two-sided Student’s t-test, *P < 0.05; **P < 0.01; ***P < 0.001; ns, not significant.

Consistent with prior reports of c-Myc depletion mediated EBV lytic cycle reactivation in Akata cells [11], c-Myc KO by both sgRNAs significantly elevated BZLF1 expression in P3HR1 cells (Fig 6M). Notably, although c-Myc was proposed to act at the level of BZLF1 through chromosome looping to OriLyt region to control EBV lytic replication [11], no direct binding of c-Myc was established in the BZLF1 promoter/Zp region. After inspection of EBV Zp using JASPAR database, in contrast to three E2F1 binding sites, one putative c-Myc binding site was identified (Fig 7A). While significant decrease in luciferase activities were observed in HEK293 cells transfected with Zp in the presence of either E2F1 or c-Myc expression construct in a dose dependent manner, as opposed to c-Myc, E2F1 demonstrated considerably higher suppressive activity of Zp (Fig 7B). The c-Myc-mediated transcriptional repression of Zp activity was further confirmed by mutational analysis of the single c-Myc binding site (Fig 7C). Additionally, no synergistic effect between c-Myc and E2F1 mediated transcriptional suppression of Zp activity was observed (Fig 7B-7C). Depletion of c-Myc expression during lytic cycle reactivation (Figs 6B and S15A and S5 Table) combined with BZLF1 mediated E2F1 transcriptional repression (Fig 2A-2J) prompted us to further investigate BZLF1’s effect on c-Myc transcription (S15B-S15C Fig). However, ChIP-Seq profile (E-MTAB-7788) and luciferase reporter analysis demonstrated neither BZLF1 occupies in the c-Myc promoter region nor it influences c-Myc promoter activity (S15B-S15C Fig). In sum, these results are consistent with a model in which E2F1 transcriptionally activates c-Myc and they independently repress BZLF1 expressions during EBV latent infection of naïve B-lymphocytes, whereas in response to periodic lytic cycle reactivation E2F1 and BZLF1 are present in a loop and negatively modulate each other’s expression (Fig 7D). BZLF1-driven restricted E2F1 expression enables in lowering the c-Myc levels, thereby supporting the maintenance of EBV lytic replication (Fig 7D).

## Discussion

In human, EBV establishes a life-long infection rendering a biphasic viral life cycle – latent period and lytic replication. While latent phase allows EBV to evade the host’s immune surveillance and develop oncogenic phenotypes, periodic lytic reactivation is essential for viral progeny production and horizontal transmission [5,9]. Moreover, accumulating evidence suggests that EBV lytic proteins contribute to cancer progression [49,50]. Despite its critical role in EBV pathogenesis, the molecular mechanisms governing the latent-to-lytic switch remain poorly understood. Studies suggest that terminal differentiation of memory B-lymphocytes into plasma cells initiates spontaneous EBV replicative cycle [8]. Moreover, recently, it has been demonstrated that c-Myc expression levels control EBV latent-to-lytic switch by altering viral three-dimensional genome architecture [11]. Our study provides critical insights into the regulatory dynamics between E2F1 and c-Myc transcription factors, elucidating their roles in EBV lytic cycle reactivation. By dissecting the mechanisms through which E2F1 suppresses EBV lytic replication and interacts with cellular and viral gene networks, our findings provide deeper insights into EBV pathogenesis and inform potential therapeutic interventions.

E2F1 regulates both cell cycle and apoptosis, and its activity is often deregulated in human tumours [27,28]. Oncogenic viruses employ a common strategy for E2F1 transactivation, which enables continuous cell proliferation. For example, simian SV40 virus encoded LT, adenovirus encoded E1A and human papilloma virus (HPV) encoded E7 disrupt the interaction between E2F1 and pRb to facilitate G1 to S phase transition of the cell cycle [51–53]. However, in case of EBV, the scenario is much complex and inadequately represented. Alike various viral oncoproteins, EBV encoded EBNA3C interacts with pRb and expedites its ubiquitin mediated degradation [54], raising the possibility of manipulating E2F1 mediated oncogenic activities. Instead, EBNA3C inhibits E2F1 mediated apoptosis under DNA-damage signals and recruits E2F6 for its transcriptional repression [22,24]. Moreover, EBNA1, the only viral oncoprotein expressed in LCLs, all varieties of EBV-associated BL and other solid tumours such as nasopharyngeal carcinoma (NPC) and gastric carcinoma (EBVaGC), activates E2F1 expression at post-transcriptional level via PI3Kδ-dependent mRNA translation stress [55]. Reanalysis of our previous lab and publicly available genome-wide transcriptome data revealed E2F1 activation during EBV infection of primary B-lymphocytes and BL cells. DNA damage responses elicit cell cycle arrest and facilitate DNA repair, ensuring overall genomic integrity. However, owing to incessant cell proliferation as well as faulty DNA repair pathways, cancer cells often exhibit ‘replication stress’, which leads to genomic instability [56]. E2F1 localizes at these DNA break sites and subsequently repairs them by recruiting homologous recombination factors [19]. It has been suggested that the ‘replication stress’ upon EBV infection represents a major barrier for naïve B-cell transformation [57]. EBNA3C can impede this EBV induced DNA damage response and thereby enabling B-cell growth transformation [57]. Although the underlying mechanism is still incomplete, it is conceivable that during latent infection EBV critically modulates E2F1 levels to mitigate DNA damage response and promote efficient B-cell transformation.

In contrast to latent infection in B-lymphocytes, our data demonstrated that EBV lytic cycle replication transcriptionally repressed E2F1 expression in both B-lymphocyte and epithelial cell background. The lytic phase is initiated by leaky expression of the IE gene, BZLF1, a key driver of lytic cycle replication, which subsequently transcriptionally activates a cascade of 30 lytic genes [11,12]. Interestingly, BZLF1 demonstrates a propensity to bind and transactivate CpG methylated viral promoters to mitigate epigenetic silencing during latent-to-lytic switch [58,59]. As opposed to BZLF1, the other IE protein BRLF1 supports lytic replication by promoting lytic gene expression from hypomethylated viral promoters [60]. Our findings establish E2F1 as a fundamental regulator of EBV latency through intervening lytic replication by transcriptionally repressing BZLF1 expression. In agreement to this, overexpression of E2F1 along with c-Myc inhibit BZLF1 mediated transactivation [26]. The transcriptional repression mediates through a negative regulatory element located at the N-terminal transactivation domain of BZLF1 [26]. Since treatments with DNA-methylation inhibitor and HDAC inhibitor promote BZLF1 expression and subsequent lytic replication [61,62], it is therefore conceivable that epigenetic regulation plays critical roles in lytic cycle reactivation. Recently, genome-wide CRISPR/Cas9 screening identified UHRF1, an E3 ubiquitin ligase, and its DNA methyltransferase partner DNMT1 as critical determinants for EBV latency programs [63]. Towards stably maintaining DNA methylation, DNMT1 requires UHRF1 [64]. UHRF1 depletion transforms latency I to latency III by de-repressing EBNA and LMP gene expressions [63]. UHRF1 silencing also robustly increases BZLF1 expression in EBV positive cells [63]. Importantly, both UHRF1 and DNMT1 are the transcriptional targets of E2F1 [65–67], where activated MEK/ERK pathway functions as a driving force [67]. We speculate that in addition to direct transcriptional regulation, E2F1 may also be involved in epigenetic regulation of BZLF1 repression through adjusting UHRF1 expression level. In agreement to these, reanalysis of RNA-Seq data (GSE237484, GSE136597) demonstrated that several E2F1 targeted genes including UHRF1 expressions were significantly downregulated in latency III EBV positive B-cells as well as in response to EBV lytic cycle reactivation. This also raises the possibility of exploiting UHRF1 inhibitors [68] as a potential therapeutic strategy for EBV-associated B-cell malignancies by inhibiting E2F1 downstream activities and thereby inducing EBV lytic replication.

Our study supports a model in which E2F1 and BZLF1 form a negative transcriptional feedback loop, ensuring a tightly regulated transition between EBV latency and lytic replication. While during latent phase E2F1 transcriptional network is maintained above a threshold level to suppress BZLF1, we speculate that leaky BZLF1 expression owing to spontaneous B-cell differentiation into plasmocytes [17] is enough to counteract E2F1 activities, thus orchestrating efficient viral lytic replication. Interestingly, BLIMP1, a master regulator of plasma cell development, positively regulates BZLF1 expression, also transcriptionally represses mature B-cell gene expression program including E2F1 [69]. Likewise, another essential transcription factor for plasma cell differentiation XBP-1 stimulates BZLF1 expression [16], may also repress E2F1 activity by an incompletely characterized mechanism [70]. These findings are consistent with our model implicating the inverse correlation of E2F1 and BZLF1 as a key determinant of contradictory EBV-infected B-cell states. BZLF1 is known to both activate and repress the transcription of viral and host genes [71–73]. The mechanisms underlying BZLF1-mediated transcriptional activation have been well characterized for both viral and cellular promoters. Specifically, BZLF1 activates gene transcription by binding to a specific seven-nucleotide DNA sequence, known as the Zta response element (ZRE), via its DNA-binding domain within bZIP. In contrast, BZLF1 represses gene transcription through multiple mechanisms. In many instances, transcriptional repression results from BZLF1’s specific interactions with host transcription factors via its N-terminal TAD or C-terminal bZIP regions [72]. For example, BZLF1 represses the expression of the tumor necrosis factor receptor 1 (TNFR1) by interacting with C/EBPα and C/EBPβ through its bZIP domain, thereby preventing their recruitment to the TNFR1 promoter [74]. Similarly, although the precise interacting partners remain unidentified, BZLF1 represses expression of the MHC class II transactivator CIITA by recruiting unknown transcription factors via its TAD [71]. In both examples, DNA-binding activity is dispensable for BZLF1-mediated transcriptional repression. Conversely, our group recently demonstrated that BZLF1 suppresses transcription of carbonic anhydrase 9 (CA9) during EBV lytic reactivation by directly binding to the CA9 promoter and utilizing its TAD [4]. In the present study, we further show that, unlike wild-type BZLF1, a mutant lacking the TAD fails to repress E2F1 promoter activity. Together, these findings support a model in which BZLF1, upon EBV lytic cycle induction, represses host gene expression through a TAD-dependent mechanism.

The regulatory axis involving E2F1 and c-Myc adds another layer of complexity to EBV latency maintenance. Our results demonstrate that during latency in addition to suppress BZLF1 expression, E2F1 positively regulates c-Myc level. However, c-Myc does not reciprocally regulate E2F1, underscoring a unidirectional regulatory hierarchy. This interplay highlights the role of c-Myc as a co-regulator of EBV latency, acting in concert with E2F1 to maintain suppression of the lytic cycle. Accumulating evidence strongly implicates c-Myc as one of the key factors in transforming naïve B-lymphocytes as well as sustaining viral latency by suppressing BZLF1 transcription [11,26,75]. In conjunction with previous data, our results also demonstrate that c-Myc depletion enhances BZLF1 expression and thereby accelerates EBV lytic replication. While several reports indicated cross-regulation between E2F1 and c-Myc [42,44,45], to our knowledge, has not previously been implicated in EBV associated B-cell malignancies. Our study provides compelling rationale for repressing EBV lytic cycle replication, where E2F1 forms a feedforward loop for transcriptional activation of c-Myc, together which limit BZLF1 expression and thereby lytic reactivation. Our data supports a lytic reactivation model, where BZLF1’s ability to repress E2F1 and thereby c-Myc transcription ensures a sustained lytic replication phase, while it fails to directly modulate c-Myc expression.

While our study provides a robust framework for understanding E2F1’s role in EBV reactivation, several questions remain unanswered. The mechanisms underlying cell type-specific differences in E2F1 activity and its interplay with other transcription factors during EBV infection warrant further investigation. Our study was exclusively focussed on E2F1 downstream modulator c-Myc, as evident from the top 100 hits of E2F1 knockdown P3HR1 cells. However, there are strong possibilities that additional hits may also function as potential repressors of EBV lytic reactivation. For example, cellular kinase and cyclin B partner CDK1 was identified as an interacting partner of EBV lytic cycle kinase BGLF4 [65]. It has been suggested that while BGLF4 mediated kinase activity is essential for an effective lytic replication, it significantly interferes EBNA1-directed latent replication [76]. Another EBV lytic protein BDLF4 phosphorylation by S-phase cyclin complexes – cyclin A/CDK2 and cyclin E/CDK2 but not by cyclin B/CDK1 is important to initiate viral late gene expression [77]. We speculate that cyclin B/CDK1 complex is important for EBV latency maintenance and it will therefore be of interest to examine CDK1 depletion in viral lytic cycle reactivation. Additionally, understanding how chromatin remodelling influences E2F1-mediated transcriptional regulation could provide deeper insights into its role in EBV latency maintenance and reactivation into lytic cycle replication. An objective of future studies will be to delineate how both positive and negative upstream regulators of E2F1 control EBV latent-to-lytic switch program.

Our findings accentuate the therapeutic potential of targeting E2F1 and its regulatory circuits to modulate EBV lytic replication and treat associated B-cell malignancies. In sum, our study elucidates the intricate transcription network of E2F1/c-Myc axis, which governs the latent-to-lytic switch in EBV positive cells. As similar to c-Myc [11], our results also demonstrated that E2F1 depletion significantly enhances virion production, even in the absence of chemical inducers. This finding aligns with the therapeutic concept of ‘lytic induction therapy’, which aims to transition latent EBV-infected cells into the lytic phase, rendering them susceptible to antiviral treatments [14,78]. Thus, the ability to modulate E2F1 transcription network with minimal cytotoxicity holds promise for improving the clinical management of EBV-associated neoplasms.

## Materials and methods

### Cell lines

HEK293, HEK293T, Lenti-X 293T cells were cultured in Dulbecco’s modified Eagle’s medium (DMEM) (Gibco) supplemented with 10% FBS (Gibco) and 1% Penicillin-Streptomycin Solution (Gibco). HEK293T-BAC-GFP-EBV cells [79] were maintained in complete DMEM containing 1 µg/ml puromycin (Merck). EBV positive BL lines - P3HR1, Jiyoye, Namalwa and EB3 were obtained from NCCS cell repository, Pune, India (https://www.nccs.res.in/cellrepository). The cell lines were authenticated by short tandem repeat (STR) profiling. 16 STR loci (amelogenin, D5S818, D21S11, D7S820, CSF1PO, D2S1338, D3S1358, vWA, D8S1179, D16S539, TPOX, TH01, D19S433, D18S51, FGA and D13S317) were amplified using AmpFLSTR Identifiler Plus PCR Amplification Kit (Applied Biosystems). The PCR amplicons were subsequently analyzed on a 3500 Genetic Analyzer (Applied Biosystems) according to the manufacturer’s protocol. The data were analyzed using Gene Mapper ID-X Software v1.5 (Applied Biosystems) to categorize peaks by size with respect to an internal standard allelic ladder. The STR data were 100% matched with ATCC STR profile database within the DSMZ online STR database (http://www.dsmz.de/fp/cgi-bin/str.html). This database includes STR data sets of more than 3600 human cell lines from ATCC, DSMZ, JCRB and RIKEN. EBV transformed lymphoblastoid cells - LCL#1 and LCL#89 [4] and BL lines – P3HR1, Jiyoye, EB3, Namalwa and DG75 were maintained in RPMI 1640 (Gibco) supplemented with 10% FBS and 1% Penicillin-Streptomycin Solution. All cells were cultured at 37^0^C in a humidified environment with 5% CO_2._ All cell lines were routinely tested for *Mycoplasma* by LookOut Mycoplasma qPCR Detection Kit (Merck). The details of cell lines are provided in Table 1.

**Table 1:** Key resources table.

Reagent or Resource	Source	Identifier
Experimental Models: Cell lines		
HEK293	Gift from Rupak Datta (Indian Institute of Science Education and Research – Kolkata)	N/A
HEK293T	Dharmacon Inc.	Cat No. HCL4517
Lenti-X 293T	Gift from Debanjan Mukhopadhyay (Presidency University)	Takara Bio USA, Inc., Cat No. 632180
HEK293T-BAC-GFP-EBV	Gift from Erle S Robertson (University of Pennsylvania), PMID: 19784370	N/A
LCL#1	Gift from Erle S Robertson (University of Pennsylvania), PMID: 21347341	N/A
LCL#89	Generated from lab, PMID: 32092124	N/A
EBV^+^ Burkitt’s lymphoma cell line - P3HR1	Obtained from NCCS cell repository, India	ATCC Cat No. HTB-62
EBV^+^ Burkitt’s lymphoma cell line - Jiyoye	Obtained from NCCS cell repository, India	ATCC Cat No. CCL-87
EBV^+^ Burkitt’s lymphoma cell line - EB3	Obtained from NCCS cell repository, India	ATCC Cat No. CCL-85
EBV^+^ Burkitt’s lymphoma cell line – Namalwa	Obtained from NCCS cell repository, India	ATCC Cat No. CRL-1432
EBV- Burkitt’s lymphoma cell line – DG75	Gift from Erle S Robertson (University of Pennsylvania), PMID: 18256156	N/A
P3HR1 stably expressing E2F1 Sh-RNA	This study	N/A
P3HR1 stably expressing E2F1 sgRNA#1	This study	N/A
P3HR1 stably expressing E2F1 sgRNA#2	This study	N/A
P3HR1 stably expressing c-Myc sgRNA#1	This study	N/A
P3HR1 stably expressing c-Myc sgRNA#2	This study	N/A
P3HR1 stably expressing BZLF1	This study	N/A
Peripheral Blood Mononuclear Cells (PBMC)	HiMedia	Cat No. CL003
Recombinant DNA		
pA3F (Empty vector)	Gift from Erle S Robertson (University of Pennsylvania), PMID: 22438805	N/A
pA3M (Empty vector)	Gift from Erle S Robertson (University of Pennsylvania), PMID: 22438805	N/A
pA3F-E2F1	Gift from Erle S Robertson (University of Pennsylvania), PMID: 22438805	N/A
pA3M-E2F1	This study	N/A
pA3F-E2F2	Gift from Erle S Robertson (University of Pennsylvania)	N/A
pA3M-BZLF1	This study	N/A
pA3F-BZLF1	PMID: 38530845	N/A
pA3F-ΔTAD-BZLF1	PMID: 38530845	N/A
pEGFP-C1 (Empty vector)	Clontech laboratories, Inc	Cat No. 632470
pA3F-ΔTAD-E2F1	This study	N/A
pA3F-E2F1-TAD2	This study	N/A
pA3F-E2F2-TAD1	This study	N/A
pA3F-c-Myc	Gift from Erle S Robertson (University of Pennsylvania) PMID: 18256156	N/A
pGL3 Basic Vector	Gift from Debrya Groskreutz	RRID: Addgene_212936
pGL3-BZLF1p-WT	This study	N/A
pGL3-BZLF1p-Mut1	This study	N/A
pGL3-BZLF1p-Mut2	This study	N/A
pGL3-BZLF1p-Mut3	This study	N/A
pGL3-BZLF1p-Mut1 + 2	This study	N/A
pGL3-BZLF1p-Mut1 + 3	This study	N/A
pGL3-BZLF1p-Mut2 + 3	This study	N/A
pGL3-BZLF1p-Mut1 + 2 + 3	This study	N/A
pGL3-BZLF1p-Mut4	This study	N/A
pGL3-E2F1p-WT	This study	N/A
pGL3-E2F1p-Mut1	This study	N/A
pGL3-E2F1p-Mut2	This study	N/A
pGL3-E2F1p-Mut3	This study	N/A
pGL3-Wp	This study	N/A
pGL3-Cp	This study	N/A
pGL3-Qp	This study	N/A
pGL3-LMP1p	This study	N/A
pGL3-LMP2p	This study	N/A
pGL3-OriLytLp	This study	N/A
pGL3-OriLytRp	This study	N/A
pGL3-Mycp-WT	This study	N/A
pGL3-Mycp-Mut1	This study	N/A
pGL3-Mycp-Mut2	This study	N/A
psPAX2	Gift from Didier Trono	RRID: Addgene_12260
pMDG	Gift from Simon Davis	RRID: Addgene_187440
pTRIPZ- shE2F1	This study	N/A
lentiCRISPR v2	Gift from Feng Zhang	RRID: Addgene_52961
lentiCRISPR v2-sgE2F1-1	This study	N/A
lentiCRISPR v2-sgE2F1-2	This study	N/A
lentiCRISPR v2-sgMyc-1	This study	N/A
lentiCRISPR v2-sgMyc-2	This study	N/A
pSG5-BRLF1	Gift from S. Diane Hayward	RRID: Addgene_72635
pA3F-BRLF1	This study	N/A
pLVX-TetOne-Puro-BZLF1	This study	N/A
Antibodies		
Anti-BZLF1/ ZEBRA (BZ1; Mouse monoclonal)	Santa Cruz Biotechnology Inc.	Cat No. sc-53904
Anti- EBV EaD (0261; Mouse monoclonal)	Santa Cruz Biotechnology Inc.	Cat No. sc-58121
Anti-E2F1 (KH95; Mouse monoclonal) for immunoblot assays	Santa Cruz Biotechnology Inc.	Cat No. sc-251
Anti-E2F1 (KH95; Mouse monoclonal) for ChIp-Seq and ChIP-qPCR analysis	Invitrogen/Thermo Fisher Scientific	Cat No. 32–1400
Anti-GAPDH (6C5; Mouse monoclonal)	Santa Cruz Biotechnology Inc.	Cat No. sc32233
Anti-GFP (Rabbit polyclonal)	Abcam Inc.	Cat No. ab290
Anti-Ubiquitin (P4D1; Mouse monoclonal)	Santa Cruz Biotechnology Inc.	Cat No. sc-8017
Anti-Flag (M2; Mouse monoclonal)	Sigma-Aldrich/ Merck	Cat No. F3165
Anti-EBNA2 (PE2; Mouse monoclonal)	Abcam Inc.	Cat No. ab90543
Anti-EBNA3A (Sheep polyclonal)	Abcam Inc.	Cat No. ab16126
Anti-EBNA3B (Sheep polyclonal)	Abcam Inc.	Cat No. ab16127
Anti-EBNA3C (Sheep polyclonal)	Abcam Inc.	Cat No. ab16128
Anti-LMP1 (Rabbit monoclonal)	Abcam Inc.	Cat No. ab136633
Anti-c-Myc antibody (clone 9E10)	Sigma-Aldrich/ Merck	Cat No. MABE282
Anti-c-Myc Epitope (Myc Tag Polyclonal Antibody)	Cloud-Clone Corp.	Cat No. TAX162Ge01
Cyclin A2 (BF683; Mouse monoclonal)	Cell Signaling Technology	Cat No. 4656S
Phospho-pRb (Ser807/811; Rabbit polyclonal)	Cell Signaling Technology	Cat No. 9308T
Mouse IgG Isotype control	Invitrogen/Thermo Fisher Scientific	Cat No. 10400cc
Goat anti-Mouse IgG (H + L) Secondary Antibody, DyLight 680	Invitrogen/Thermo Fisher Scientific	Cat No. 35518
Goat anti-Mouse IgG (H + L) Secondary Antibody, DyLight 800 4X PEG	Invitrogen/Thermo Fisher Scientific	Cat No. SA5–35521
Goat anti-Rabbit IgG (H + L) Secondary Antibody, DyLight 800 4X PEG	Invitrogen/Thermo Fisher Scientific	Cat No. SA5–35571
Goat anti-Rabbit IgG (H + L) Secondary Antibody, DyLight 680	Invitrogen/Thermo Fisher Scientific	Cat No. 35568
Rabbit anti-Sheep IgG (H + L) Cross-Adsorbed Secondary Antibody, DyLight 680	Invitrogen/Thermo Fisher Scientific	Cat No. SA5–10058
Rabbit anti-Sheep IgG (H + L) Cross-Adsorbed Secondary Antibody, DyLight 800	Invitrogen/Thermo Fisher Scientific	Cat No. SA5–10060
Goat Anti-Human IgG HL	Abcam Inc.	Cat No. ab6714
Chemicals, Enzymes and other reagents		
12-O-Tetradecanoylphorbol- 13-acetate (TPA)	Merck/Sigma-Aldrich	Cat No. P1585
Sodium butyrate (NaBu)	Merck/Sigma-Aldrich	Cat No. B5887
Polybrene	Merck/Sigma-Aldrich	Cat No. TR-1003-G
Puromycin dihydrochloride	Merck/Sigma-Aldrich	Cat No. P8833
MG132	Abcam Inc.	Cat No. ab141003
Mimosine	MedChemExpress	Cat No. HY-N0928
Doxycycline hyclate	Merck/Sigma-Aldrich	Cat No. D9891
Propidium iodide	Merck/Sigma-Aldrich	Cat No. P4170
Triton X-100	Merck/Sigma-Aldrich	Cat No. T8787
RNase A	New England Biolabs	Cat No. T3018L
*Eco* RI	New England Biolabs	Cat No. R0101S
*Not* I	New England Biolabs	Cat No. R0189S
*Bgl* II	New England Biolabs	Cat No. R0144S
*Mlu* I-HF	New England Biolabs	Cat No. R3198S
*Bsm*BI-v2	New England Biolabs	Cat No. R0739S
T4 DNA Ligase	New England Biolabs	Cat No. M0202S
DNase I (RNase-free)	New England Biolabs	Cat No. M0303S
PureZOL RNA Isolation Reagent	BIO-RAD	Cat No. 7326880
iScript cDNA Synthesis Kit	BIO-RAD	Cat No. 1708890
iTaq Universal SYBR Green Supermix	BIO-RAD	Cat No. 1725125
Q5 High-Fidelity 2X Master Mix	New England Biolabs	Cat No. M0492S
T4 Polynucleotide Kinase Reaction Buffer	New England Biolabs	Cat No. B0201S
Proteinase K	New England Biolabs	Cat No. P8107S
Q5 Site-Directed Mutagenesis Kit	New England Biolabs	Cat No. E0554S
Wizard Genomic DNA Purification Kit	Promega	Cat No. A1120
QIAquick PCR purification kit	Qiagen	Cat No. 28104
QIAprep Spin Miniprep Kit	Qiagen	Cat No. 27104
EndoFree Plasmid Mega Kit	Qiagen	Cat No. 12381
Dual-Luciferase Reporter Assay System	Promega	Cat No. E1910
SureBeads Protein G Magnetic Beads	BIO-RAD	Cat No. 161–4023
ChIP-IT Express Chromatin Immunoprecipitation Kit	Active Motif	Cat No. 53008
RIPA Lysis and Extraction Buffer	Thermo Fisher Scientific	Cat No. 89901
Protease Inhibitor Cocktail	Abcam Inc.	Cat No. ab65621
Quick Start Bradford 1x Dye Reagent	BIO-RAD	Cat No. 5000205
2x Laemmli Sample Buffer	BIO-RAD	Cat No. 1610737
Nitrocellulose Membrane	BIO-RAD	Cat No. 1620115
Software		
DAVID v6.8	DAVID Bioinformatics	https://david.ncifcrf.gov/
Microsoft Excel 2013	Microsoft	www.microsoft.com/
GraphPad Prism	GraphPad Software	www.graphpad.com
ZOE Fluorescent Cell Imager	BIO-RAD	https://www.bio-rad.com/en-in/product/zoe-fluorescent-cell-imager?ID=N74CIZE8Z
Image Studio v2.0	LI-COR Biosciences	www.licor.com/bio/image-studio/
CFX- Maestro v2.3	BIO-RAD	www.bio-rad.com/
GTEx	GTEx Portal	https://gtexportal.org/home/
ImageLab v5.1	BIO-RAD	www.bio-rad.com/
Integrative Genomics Viewer (IGV)	Broad Institute	https://igv.org/app/
ChIP-Atlas	Kyoto University	https://chip-atlas.org/
JASPAR	JASPAR 2024	https://jaspar.genereg.net/
DepMap	Broad Institute	https://depmap.org/portal/
FCS Express v6.06.0042	De Novo Software	https://denovosoftware.com/
NEBaseChanger v2.5.2 tool	New England Biolabs	https://nebasechanger.neb.com/
Synthego	https://www.synthego.com/contact	https://www.synthego.com/
AlphaFold Protein Structure Database	EMBL-EBI	https://alphafold.ebi.ac.uk/
UniProt Align tool	UniProt	https://www.uniprot.org/align
Cell culture and transfection		
DMEM, high glucose	Gibco/Thermo Fisher Scientific	Cat No. 11965118
RPMI 1640	Gibco/Thermo Fisher Scientific	Cat No. 11875119
Opti-MEM	Gibco/Thermo Fisher Scientific	Cat No. 31985062
Fetal Bovine Serum (FBS)	Gibco/Thermo Fisher Scientific	Cat No. 10438026
Trypsin-EDTA (0.05%), phenol red	Gibco/Thermo Fisher Scientific	Cat No. 25300054
Penicillin-Streptomycin (10,000 U/mL)	Gibco/Thermo Fisher Scientific	Cat No. 15140122
PBS (10X), pH 7.4	Gibco/Thermo Fisher Scientific	Cat No. 70011069
DMSO, Anhydrous	Invitrogen/Thermo Fisher Scientific	Cat No. D12345
LookOut Mycoplasma qPCR Detection Kit	Merck	Cat No. MP0040A-1KT
Gene Pulser/MicroPulser Electroporation Cuvettes, 0.4 cm gap	BIO-RAD	Cat No. 1652088
Calcium Phosphate Transfection Kit	Invitrogen/Thermo Fisher Scientific	Cat No. K278001
jetPRIME	Polyplus Transfection Inc.	Cat No. 101000015

### Bioinformatic analysis of sequencing data

All raw sequencing reads from high throughput sequencing pipelines were first checked using FastQC (http://www.bioinformatics.babraham.ac.uk) and confirmed with no significant quality issues.

Bulk RNA-Seq data analysis: The raw Fastq files of transcriptome data of EBV infected PBMCs 0–4 DPI (GSE235941), EBV infected naïve B-lymphocytes 0–28 DPI (GSE125974), EBV lytic cycle reactivation from LCLs (GSE237484), Mutu I and Mutu III (GSE136597) and Akata c-Myc KO (GSE140653) were downloaded and extracted with unique SRR accession in Galaxy webserver (https://usegalaxy.org/) and complete analysis was performed using appropriate tools offered by the webserver. Data were visualized with either GraphPad Prism v8 or Microsoft Excel.

Single-cell RNA-Seq data analysis: Publicly available scRNA-seq data (GSE272763) from P3HR1-ZHT cells undergoing lytic reactivation were reanalyzed using the Galaxy webserver. Raw matrix, feature, and barcode files were imported and converted to the h5ad format. Quality control was performed to exclude cells with low gene counts, high proportions of mitochondrial transcripts, or extreme total read counts. Following normalization and log transformation, highly variable genes were selected, and dimensionality reduction was conducted. Uniform Manifold Approximation and Projection (UMAP) was applied for two-dimensional visualization of cell clusters. Clustering was performed using the Leiden algorithm based on a nearest-neighbor graph. Differential gene expression analysis was conducted using the Wilcoxon rank-sum test to identify marker genes. UMAP plots were generated to visualize the expression of key genes and time point-specific transcriptional differences.

ChIP-Seq data analysis: The raw Fastq files of different ChIP-Seq data (BZLF1: E-MTAB-7788; E2F1: GSE76191; EBNA1: GSE73887; EBNA2: GSE29498; EBNALP: GSE49338; EBAN3A: GSE88729; EBNA3B: GSE88729; EBNA3C: GSE88729; RelA/p65: GSE55105; c-Rel: GSE55105; RelB: GSE55105; p50: GSE55105; p52: GSE55105; c-Myc: GSE30399 and GSE36354) were extracted from SRA files and aligned against human reference genome (Homo sapiens.GRCh37) using default parameters. BAM files were further analysed for peak calling using MACS2. Analysed files were visualized on the Integrative Genomics Viewer (https://igv.org/).

DepMap data analysis: To perform correlation study between E2F1 and c-Myc expressions across different EBV positive and negative B-cell lymphoma cell lines along with multiple solid cancers – lung, breast, prostate and skin carcinomas, gene expression data were extracted from DepMap Expression Public 23Q4 dataset (https://depmap.org/portal/). Correlation data was visualized using GraphPad Prism v8.

TCGA data analysis: To check the correlation between E2F1 and c-Myc expressions in diffuse large B-cell lymphoma (DLBCL), lung adenocarcinoma (LUAD), breast invasive carcinoma (BRCA), prostate adenocarcinoma (PRAD) and skin cutaneous melanoma (SKCM) patients’ samples UCSC Xena browser (https://xenabrowser.net/) was utilized. Extracted gene expression data were further analysed using GraphPad Prism v8 data.

GTEx data analysis: The Genotype-Tissue Expression (GTEx) Portal (https://www.gtexportal.org/home/) was utilized to check the expression pattern of all eight E2F genes between EBV transformed B-cells (LCLs) and whole blood samples (PBLs).

### EBV infection of PBMCs

HEK293T-BAC-GFP-EBV cells were induced for EBV lytic cycle reactivation with 20 ng/ml 12-O-Tetradecanoylphorbol-13-acetate (TPA; Merck) and 3 mM Sodium butyrate (NaBu; Merck) for 5 days. EBV particle in the culture supernatant was concentrated by ultracentrifugation at 27,000 rpm for 2 h at 4^0^C and subsequently re-suspended in 500 µl RPMI 1640 without any supplementation. Virus stock was stored at -80^0^C for further use. ~ 1.0 x 10^7^ PBMCs (HiMedia) from two individual donors in complete RPMI 1640 were incubated with EBV (MOI: ~ 10). 24 h post-infection, cells were centrifuged, re-suspended in fresh RPMI 1640 and continued growing at 37^0^C for 28 days. Cells were harvested at the indicated time points (0–28 DPI; days post-infection) for qRT-PCR analysis. EBV lytic cycle reactivation in HEK293T-BAC-GFP-EBV cells and latent infection in PBMCs were monitored by checking green fluorescence using a ZOE Fluorescent Cell Imager (BIO-RAD).

### Quantitative real-time (qRT-PCR) analysis

For qRT-PCR analysis, ~ 1.0 x 10^7^ cells from each experimental settings were harvested for RNA isolation using PureZOL RNA Isolation reagent (BIO-RAD) following the manufacturer protocol. ~ 1 µg RNA was subjected to reverse transcription using iScript cDNA synthesis kit (BIO-RAD) according to the manufacturer’s protocol. Both quality and quantity of nucleic acids were checked in a Synergy H1 Multimode Microplate Reader (BioTek). qRT-PCR analysis was conducted using iTaq Supermix (BIO-RAD) on a CFX Connect real-time PCR detection system (BIO-RAD). Unless specified otherwise, each reaction was replicated thrice and relative transcript levels were quantified using the 2^−ΔΔCT^ method and normalized with GAPDH or B2M internal control. All samples were run in technical triplicates and at least two independent experiments were performed. The sequences of primers used for qRT-PCR are given in S6 Table.

### Immunoblot analysis

For immunoblot analysis, ~ 1.0 x 10^7^ cells were lysed in 500 µl RIPA buffer (Thermo Fisher Scientific) combined with 1x protease inhibitor cocktail (Abcam) by occasional vortexing for 15 s with 5 min interval. After estimation of total protein concentrations by Bradford reagent (BIO-RAD), protein samples were boiled with 2x laemmli buffer (BIO-RAD) at 95^0^C for 10 min. Equal amount of samples were resolved by SDS-PAGE, transferred to a nitrocellulose membrane (BIO-RAD) and blocked with 5% milk in 1x TBS. After washing with 1x TBST, the membranes were incubated with specific primary antibodies overnight at 4^0^C. Following day, the membranes were washed with 1x TBST and incubated with appropriate infrared-tagged/DyLight secondary antibodies (Thermo Fisher Scientific) for 1 h at room temperature. After washing with 1x TBST, image analysis and quantification of protein bands were achieved using the Odyssey Infrared Imaging System (LiCor Inc.). The list of primary and secondary antibodies used in immunoblot analysis are given in Table 1.

### EBV lytic cycle reactivation

For induction of EBV lytic cycle replication, ~ 1.0 x 10^7^ HEK293T cells stably transfected GFP-tagged EBV-BACmid (HEK293T-BAC-GFP-EBV), EBV positive BL lines (P3HR1, Jiyoye, EB-3) and *in vitro* EBV transformed LCLs (LCL#89) were maintained in complete media containing either combination of 20 ng/ml TPA (Merck) and 3 mM NaBu (Merck) or treated with 1 μM MG132 (Abcam) or 10 µg/ml Goat Anti-Human IgG (Abcam) as indicated. 24–72 h post-treatment the lytic reactivation was validated by either immunoblotting or qRT-PCR analysis of EBV lytic cycle transactivator BZLF1 as well as quantification of EBV genome copy number.

### ChIP-qPCR analysis

ChIP-qPCR was performed as previously described [4]. Briefly, after crosslinking and subsequent de-crosslinking ~2.0 x 10^7^ cells were harvested, washed with ice-cold 1x PBS and suspended in lysis buffer (50 mM Tris-HCl pH 8.1, 10 mM EDTA, 1% SDS and 1 × protease inhibitor cocktail). Chromatin was sonicated with a Diagenode Bioruptor Plus sonicator (Diagenode Inc.) to attain DNA fragments of ~200–400 bp as confirmed by agarose electrophoresis. 10% of the sonicated chromatin was collected and used as input material, while the remaining sheared chromatin was further diluted to immunoprecipitation (IP) dilution buffer (16.7 mM Tris-HCl pH 8.1, 1.2 mM EDTA, 167 mM NaCl, 1.1% Triton X-100, 0.01% SDS along with 1 × protease inhibitor cocktail), followed by immunoprecipitation with 5 μg appropriate antibodies (anti-E2F1 or anti-BZLF1) or corresponding mouse IgG control using magnetic protein A/G beads (BIO-RAD). After sequential washing steps with ‘low-salt wash buffer’ (20 mM Tris-HCl, pH 8.1, 2 mM EDTA, 150 mM NaCl, 1% Triton X-100, 1% SDS), ‘high-salt wash buffer’ (20 mM Tris-HCl, pH 8.1, 2 mM EDTA, 500 mM NaCl, 1% Triton X-100, 1% SDS)), ‘LiCl wash buffer’ (10 mM Tris-HCl, pH 8.1, 1 mM EDTA, 0.25 M LiCl, 1% NP-40, 1% deoxycholate acid) and ‘TE buffer’ (10 mM Tris-HCl, pH 8.1, 1 mM EDTA) the protein-DNA complexes were eluted using ‘elution buffer’ (100 mM NaHCO3, 1% SDS). Following reverse cross-linking using proteinase K treatment, the ChIP-ed DNA was purified using the QIAquick PCR purification kit (QIAGEN) and subjected for qPCR analysis. Data were analyzed by the ΔΔCT method relative to input DNA and normalized to the IgG control. The sequences of primers used for ChIP-qPCR are given in S6 Table.

### Transfection

For transient transfection assays, ~ 2.0 x 10^7^ Jiyoye, or ~1.0 x 10^7^ HEK293 or HEK293T-BAC-GFP-EBV cells were harvested and re-suspended in 450 μL Opti-MEM (Gibco), mixed with appropriate plasmids in Gene Pulser/MicroPulser Electroporation Cuvettes (BIO-RAD) followed by electroporation using Gene Pulser II electroporator (BIO-RAD). For Jiyoye, electroporation pulses were set at 240 V and 960 μF, while for HEK293 and HEK293T-BAC-GFP-EBV cells pulses were set at 210 V 975 μF. Unless and otherwise stated, 36–48 h post-transfection cells were harvested and subjected for expression analysis. For promoter assays, HEK293 cells were transfected with appropriate plasmids using JetPrime (Polyplus Transfection Inc.) according to manufacturer’s protocol. For lentivirus production, Lenti-X 293T cells were transfected with appropriate plasmids using calcium phosphate transfection kit (Thermo Fisher Scientific).

### Co-immunoprecipitation (Co-IP)

Co-IP was performed as described previously [18]. Briefly, ~ 15 x 10^6^ HEK293 cells transiently transfected with myc-tagged BZLF1 with or without flag-tagged E2F1 expression plasmids were harvested, washed with 1 x PBS and subsequently lysed with 500 µl RIPA buffer supplemented with protease inhibitor cocktail. After saving 5% of the lysate as input, remaining lysate was subjected to preclear with Protein-A/G magnetic beads (BIO-RAD) for 1h at 4^0^C. Protein of interest was captured by rotating precleared lysate with 1 μg of anti-flag mouse monoclonal antibody (Merck) overnight at 4^0^C. Following day, Immuno-complexes were captured by Protein-A/G magnetic beads, washed with RIPA buffeter for three times and boiled with 2 x laemmli buffer for 5 min. Input lysates and IP complexes were then fractionated by SDS-PAGE and subjected to immunoblot analysis as mentioned above. The data was analysed and viewed on an Odyssey CLx Imaging System.

### Construction of plasmids and Site-Directed Mutagenesis

Cellular E2F1 and c-Myc wild type promoters along with all the EBV promoters - Zp, Cp, Qp, Wp, LMP1p and LMP2p were constructed in pGL3 Basic Vector (Addgene #212936) by conventional PCR, restriction digestion with appropriate enzymes followed by ligation. Substitution based mutated primers were designed using NEBaseChanger v2.5.2 tool (https://nebasechanger.neb.com/). Primers were obtained from Integrated DNA Technologies and mutated plasmids were constructed using Q5 Site-Directed Mutagenesis Kit (New England Biolabs) according to manufacturer’s protocol. Transactivation domains (TAD) of E2F1 and E2F2 were swapped using assembly based PCR with appropriate primers, followed by restriction digestion and ligation in pCDNA3.1 based vector (pA3F with 3x Flag). All the constructs were further verified by Sanger dideoxy based DNA

sequencing (Eurofins Genomics India Pvt. Ltd., India). The sequences of primers used for cloning are given in S6 Table.

### Luciferase-based promoter assay

Dual-Glo Luciferase Assay Systems kit (Promega) was used for promoter assays according to manufacturer’s protocol. Briefly, ~ 2–3 x 10^5^ HEK293 cells were seeded prior to transfection in 12-well plates (Corning Inc.). Cells were transiently transfected with specific pGL3 promoter plasmids (wild type or mutant) in the presence of appropriate vector control or expressing plasmids. 36 h post-transfection cells were harvested, washed with 1 x PBS and suspended with 100 μl of 1 x passive lysis reagent (PLB). 20 μl of the cell extract supernatant was mixed with 100 μl of Luciferase Assay Reagent (LAR) and subjected for luminescence measurement in Synergy H1 microplate reader (BioTek) after a 5 sec delay over a 10 sec window.

### Chromatin immunoprecipitation sequencing (ChIP-Seq)

A total of 2.0 × 10^7^ cells were cross-linked with 1% formaldehyde for 10 min followed by quenching the reaction with 125 mM glycine for 5 min at room temperature. ChIp-Seq was performed using 5 µg E2F1 specific mouse monoclonal antibody (Invitrogen) in two *in vitro* EBV transformed lymphoblastoid cell lines (LCL#1 and LCL#89) using ChIP-IT Express Chromatin Immunoprecipitation Kit (Active Motif, Inc.) according to the manufacturer’s instruction. ChIP libraries were generated using the NEBNext Ultra II DNA Library preparation kit (New England Biolabs). ChIP Libraries were validated using Qubit 4 fluorometer (Thermo Fisher Scientific) followed by next-generation sequencing analysis on an Illumina HiSeq2500 platform. Reads quality were checked using FastQC followed by adapter trimming with Trimmomatic v0.35. The paired end data aligned to the reference Human (Homo sapiens.GRCh37) using Bowtie2 v 2.5.3 with default parameters. Paired end reads were also aligned with Human gammaherpesvirus 4 genome sequence (RefSeq: NC_007605.1). MACS2 call-peak v2.2.9.1 was used for peak calling analysis against the matching input samples. ChIP-seq signal tracks were visualized using the Integrative Genomics Viewer (https://igv.org/).

### Quantification of EBV genome copy number

Quantification of intracellular and extracellular EBV genome copy number was performed by qRT-PCR analysis. Intracellular viral DNA was extracted from ~3 × 10^6^ cells by Wizard Genomic DNA Purification Kit (Promega). For extracellular viral DNA isolation, 600 μl cell supernatant was collected and centrifuged at 3000 rcf for 10 min. The supernatant was then treated with 15 μl DNase I (New England Biolabs) at 37^0^C for 30 min followed by heat inactivation at 70^0^C for 10 min. The supernatant was further treated with 15 μl Proteinase K (800 U/ml, New England Biolabs), 100 μl of 10% (wt/vol) SDS and incubated for 60 min at 65^0^C. DNA was purified using phenol-chloroform method followed by precipitation with sodium acetate and ethanol. Precipitated DNA was dissolved in 50 μl nuclease-free water. The extracted DNA was then diluted to 10 ng/μl and qPCR was performed targeting the EBNA1 viral gene. A standard curve was made by performing qPCR on serial dilutions of Namalwa EBV genome targeting the EBNA1 as previously described [80]. EBV viral copy number was calculated by putting the sample Cq values into the regression equation provided by the standard curve.

### Lentivirus mediated knockdown (KD) of E2F1 in EBV positive B-cells

Lenti-X 293T cells at ~70% confluency in 10-cm cell culture dishes (Corning Inc.) were co-transfected with 10 µg pTRIPZ-sh-E2F1 clone along with the two lentivirus packaging plasmids - 4 µg pMDG (Addgene #187440) and 12 µg psPAX2 (Addgene #12260) using CaPO_4_ method as previous described [4]. E2F1 Sh-RNA sequence was adopted from previously published results [81]. 12 h post transfection media was replaced with fresh DMEM with 3 mM NaBu (Merck) to induce lentivirus replication cycle. 48 h post-treatment lentivirus containing media was collected, filtered through 0.45 μM membrane (Corning Inc.) and subjected to spinoculation with ~5.0 x 10^5^ P3HR1 cells in complete RPMI supplemented with 8 μg/ml polybrene (Merck) at 800 g for 2 h. 48 h post-transduced cells were selected using 1 μg/ml puromycin (Merck) for 7 days. Expression of Sh-RNA in transduced P3HR1 cells was initiated by 1 μg/ml doxycycline (Merck), while transduced cells without doxycycline treatment were served as control. Selected cells with or without doxycycline were harvested and subjected to immunoblot, qRT-PCR, cell proliferation and EBV genome copy number quantification analysis. Oligo sequences for E2F1 knockdown are available in S6 Table.

### sgRNA CRISPR knockout (KO) analysis

Two individual sets of E2F1 and c-Myc sgRNAs were either designed using the online tool Synthego (https://www.synthego.com/products/bioinformatics/crispr-design-tool) or adopted from previously published manuscripts [11,28]. Oligos were obtained from Integrated DNA Technologies, annealed and subsequently cloned into the *Bsm*BI restriction site of the lentiCRISPR v2 vector (Addgene #52961). Lentivirus production followed by spinoculation in P3HR1 cells were performed as described above for stable cell line generation. KO efficiency was validated by immunoblot analysis. Oligo sequences for E2F1 and c-Myc knockout are available in S6 Table.

### Cell cycle analysis

Cell cycle analysis was performed as previously described [4]. Briefly, P3HR1 cells were either left untreated or treated with the combination of 20 ng/ml TPA and 3 mM sodium butyrate or specific cell cycle inhibitor 50 μM Mimosine (MedChemExpress) for G0/G1 arrest. 24 h post-treatment cells were washed with 1 x PBS and fixed with ice-cold 70% ethanol for 30 min at 4^0^C followed by two additional washing steps with 1 x PBS. Fixed cells were treated with staining buffer (5 µg/ml propidium iodide, 40 µg/ml RNase A, 0.1% Triton X-100 in PBS) for 30 min at room temperature. Each sample was subjected for cell cycle analysis on an S3e Cell Sorter (BIO-RAD). Cell cycle data were analyzed using FCS Express v6.06.0042 (https://denovosoftware.com/).

### Structure prediction and pairwise structure alignment

The predicted structure of E2F1 and E2F2 were acquired using the AlphaFold Protein Structure Database (https://alphafold.ebi.ac.uk/) [82]. The primary sequence of E2F1 and E2F2 was obtained from UniProt accession IDs - Q01094 and Q14209, respectively. The default parameters of AlphaFold framework were used for the prediction. The confidence of the predicted model was assessed using the per-residue predicted local distance difference test (pLDDT) scores provided by AlphaFold. AlphaFold structure viewer tool was used to perform further structural visualization. Alignment of 3D structures of E2F1 and E2F2 was performed using the RCSB PDB Pairwise Structure Alignment tool.

### Sequence similarity consensus analysis

UniProt Align tool (https://www.uniprot.org/align) was used to check the sequence similarity of all E2F transcription factors. Protein sequences of all the E2Fs were extracted from UniProt database (accession IDs: E2F1 - Q01094, E2F2 - Q14209, E2F3 - O00716, E2F4 - Q16254, E2F5 - Q15329, E2F6 - O75461, E2F7 - Q96AV8, E2F8 - A0AVK6) and aligned using default parameters of the UniProt Align tool. Percentage similarity was determined based on the presence of identical residues in the aligned regions. Pairwise alignment and the resulting similarity score was downloaded from the UniProt Align tool for further analysis.

### RNA sequencing and data analysis

P3HR1 cells with or without doxycycline (DOX) containing RPMI for 7 days were either left untreated or treated with EBV lytic cycle inducer - 3 mM NaBU plus 20 ng/ml TPA. 72 h post-induction cells in all four categories were harvested and subjected to RNA isolation. ~ 1 µg total RNA was used for library generation using NEBNext Ultra II Directional RNA library Prep Kit (New England Biolabs) followed by RNA sequencing analysis on an Illumina NovaSeq 6000 platform according to the manufacturer’s instructions. For read quality reports FastQC was applied and qualified reads were processed with Trimmomatic v0.35 for trimming the adapter sequences. The sequences were aligned to the Human genome (Homo sapiens.GRCh37) using Bowtie2 v 2.5.3 with default parameters. Gene expression was measured using featureCounts v 2.0.6.

DESeq2 v 2.11.40.8 package from R was utilized to analyse differential expression pattern between experimental groups. Up-regulated and down-regulated genes were selected on the basis of log_2_Fold Change as> = 1.5 and <= -1.5 respectively with p value < = 0.05. Differentially expressed gene sets were further analysed through DAVID v6.8 webserver. Functional analysis was performed by clustering features found across different databases. Gene Ontology (GO) was selected from the hits table for DAVID clustering. The abundance of viral transcripts were quantified utilizing Kallisto quant tool (v. 0.48.0) with Human herpesvirus 4 type 2 reference transcriptome (RefSeq: NC_009334.1) into transcripts per million (TPM), which was further converted to log_2_ (TPM + 1) for data representation.

### Statistical analysis

Unless otherwise indicated, all bar and line graphs denote the arithmetic mean of at least three biologically independent experiments (n = 3), with error bars representing standard deviations (SD). Data were analysed using One-Way Anova (Tukey’s multiple comparison test) followed by two tailed student’s t-test or post-Dunnett test to calculate the Statistical significance of differences in the mean values using either GraphPad Prism v8 or Microsoft Excel 2013 software. P-value < 0.05 was considered as significant (*P < 0.05; **P < 0.01; ***P < 0.001; ns, not significant).

## Supporting information

S1 Fig Differential gene expressions of E2F members during EBV latent infection and lytic cycle reactivation. (A) Heat map analysis (log2 Fold Change) of all eight E2F genes (E2F1-8) of RNA-Seq data (GSE235941) of peripheral blood mononuclear cells (PBMCs) infected with GFP-EBV for 0–4 days post-infection (dpi). (B) Heat map representation of reanalysis of microarray data [29] of E2F transcripts (E2F1-8) in uninfected and EBV infected BL31 cells. (C) Heat map representation of differential gene expression of the E2F genes (E2F1-8) of RNA-Seq data (GSE125974) of B-cells infected with EBV for 0–28 dpi. (D) qRT-PCR analysis of cDNA generated from PBMCs from two individual donors infected with GFP-EBV for 0–28 dpi. (E) Heat map and dot plot analysis of the transcripts profile of the indicated E2F genes in whole blood cells (PBLs) and EBV transformed lymphoblastoid cell lines (LCLs) using ‘Genotype-Tissue Expression (GTEx)’ project. (F) qRT-PCR analysis of cDNA generated from PBMCs from two individual donors and two LCLs – LCL#1 and LCL#89. (G) Heat map representation of differential gene expression of the E2F genes (E2F1-8) of RNA-Seq data (GSE237484) of two LCLs (LCL#1 and LCL#89) reactivated to lytic replication by TPA-NaBu treatment for 0–3 days post treatment (dpt). (H) Heat map representation of differential gene expression of the E2F genes (E2F1-8) of RNA-Seq data [18] of two LCLs (LCL#1 and LCL#89) either left untreated or treated with 1 µM MG132 for 24 h. qRT-PCR results are presented as the mean ± SD, n = 3 biological replicates. Statistical significance was determined by a two-sided Student’s t-test, *P < 0.05; **P < 0.01; ***P < 0.001; ns, not significant.
(TIF)

S2 Fig Effect of doxycycline treatment on BZLF1 and E2F1 expressions in P3HR1 cells. P3HR1 cells were subjected to immunoblot analysis without or with doxycycline (-/ + DOX) treatment for 72 h.
(TIF)

S3 Fig Effect of TPA-sodium butyrate (NaBu) and MG132 treatments on E2F1 expression. (A) HEK293 cells were treated with 20 ng/ml TPA and 3 mM sodium NaBu treatment for the indicated time points (0–72 h) and subjected to immunoblot analysis. (B) HEK293 cells were treated with 1 μM MG132 for the indicated time points (0–48 h) and subjected to immunoblot analysis.
(TIF)

S4 Fig ChIP-Seq reanalysis reveal latent EBV oncoproteins do not occupy E2F1 promoter. ChIP-Seq tracks for EBV oncoproteins - EBNA1 (GSE73887), EBNA2 (GSE29498), EBNALP (GSE49338), EBNA3A (GSE88729), EBNA3B (GSE88729), EBNA3C (GSE88729), and NF-ĸB subunits (GSE55105) - RelA/p65, c-Rel, RelB, p50, p52 at E2F1 promoter region.
(TIF)

S5 Fig Unlike BZLF1, the other EBV immediate early protein, BRLF1, does not suppress E2F1 expression. (A) Immunoblot analysis of HEK293 cells transiently transfected with flag-tagged BZLF1 expression plasmid. (B) Immunoblot analysis of HEK293 cells transiently transfected with flag-tagged BRLF1 expression plasmid.
(TIF)

S6 Fig BZLF1 does not affect E2F1 expression at post-translational level. (A) Immunoblot analysis of HEK293 cells transiently transfected with flag-tagged E2F1 expression plasmid in the presence of either control vector or myc-tagged BZLF1 expression plasmid. (B) HEK293 cells transiently transfected myc-tagged BZLF1 expression plasmid with or without flag-tagged E2F1 expression plasmid were subjected to co-immunoprecipitation analysis using anti-flag antibody.
(TIF)

S7 Fig E2F1 and BZLF1 mutually repress each other’s promoter activity in B-cells. (A) Luciferase reporter activity and the corresponding immunoblot analysis of the wild-type E2F1 promoter in the presence of increasing concentrations of BZLF1 expression plasmid in transiently transfected EBV^-^ DG75 cells. (B) Luciferase reporter activity and the corresponding immunoblot analysis of the wild-type BZLF1 promoter (Zp) in the presence of increasing concentrations of E2F1 expression plasmid in transiently transfected EBV^-^ DG75 cells.
(TIF)

S8 Fig bZIP domain of BZLF1 is essential for DNA binding activity at E2F1 promoter region. (A) Schematic showing different structural domains of BZLF1 for cloning in a flag-tagged expression vector. (B) Immunoblot analysis of HEK293 cells transiently transfected with control vector or flag-tagged expression plasmids for wild-type (WT), ΔTAD and ΔbZIP BZLF1 proteins. (C) ChIP-qPCR data showing recruitment of flag-tagged WT and ΔTAD BZLF1 proteins at E2F1 promoter region in transiently transfected HEK293 cells. The results are presented as the mean + SD, n = 3 biological replicates. Statistical significance was determined by a two-sided Student’s t-test, *P < 0.05; **P < 0.01; ***P < 0.001; ns, not significant.
(TIF)

S9 Fig Effect of E2F1 on EBV latent promoters. Luciferase reporter activity and the corresponding immunoblot analysis of different EBV latent promoters – Wp, Cp, Qp, LMP1p and LMP2p in the presence of increasing concentrations of E2F1. All the experiments were performed in HEK293 cells. The results are presented as the mean ± SD, n = 3 biological replicates. Statistical significance was determined by a two-sided Student’s t-test, *P < 0.05; **P < 0.01; ***P < 0.001; ns, not significant.
(TIF)

S10 Fig E2F1 expression controls EBV lytic replication. (A) Immunoblot analysis of P3HR1 cells stably expressing E2F1 sh-RNA (P3HR1-sh-E2F1) in the absence and presence of doxycycline (-/ + DOX) either left untreated or or treated with TPA-NaBu for 72 h. (B) EBV intracellular or extracellular genome copy number analysis was performed on cDNA isolated from P3HR1-sh-E2F1 cells in the absence and presence of doxycycline (-/ + DOX) either left untreated or treated with TPA-NaBu for 72h. The results are presented as the mean ± SD, n = 3 biological replicates. Statistical significance was determined by a two-sided Student’s t-test, *P < 0.05; **P < 0.01; ***P < 0.001; ns, not significant.
(TIF)

S11 Fig Cell cycle arrest does not affect E2F1 expression nor EBV lytic cycle activation. (A) Cell cycle analysis of P3HR1 cells either left untreated or treated with Mimosine or TPA-NaBu for 24 h. (B) Cell cycle phase quantification data of P3HR1 cells either left untreated or treated with Mimosine or TPA-NaBu for 24 h. (C) Immunoblot analysis of P3HR1 cells either left untreated or treated with Mimosine or TPA-NaBu for 24 h. (D) Schema showing deletion of pocket protein binding domain (PBD) of E2F1 for cloning into a flag-tagged expression vector. (E) Immunoblot analysis of Jiyoye cells transiently transfected with control vector, flag-tagged wild-type (residues 1–437) E2F1 or pocket protein binding domain deleted E2F1 (residues 1–400) expression plasmids. Cell cycle distribution graphs and blots are representative of n = 3 biological replicates.
(TIF)

S12 Fig E2F1 knockdown transactivates EBV lytic genes. (A) Heatmap analysis of RNA-Seq data of EBV transcripts from P3HR1 cells stably expressing E2F1 sh-RNA (P3HR1-sh-E2F1) in the absence and presence of doxycycline (-/ + DOX) and with or without TPA-NaBu treatment for 72 h. Log2 (TPM + 1) in EBV mRNA abundance are shown. (B) qRT-PCR analysis of EBV latent and lytic gene mRNAs from P3HR1-sh-E2F1 cells in the absence and presence of doxycycline (-/ + DOX). qRT-PCR results are presented as the mean + SD, n = 3 biological replicates. Statistical significance was determined by a two-sided Student’s t-test, *P < 0.05; **P < 0.01; ***P < 0.001; ns, not significant. (C) Reanalysis of RNA-Seq data (GSE136597) of all eight E2F genes (E2F1-8) in EBV^+^ BL lines Mutu I and Mutu III.
(TIF)

S13 Fig E2F1 and c-Myc transcripts do not positively correlate in EBV negative B-cell lymphomas and solid cancers. (A) Two-sided unpaired Student’s t-test and Pearson’s correlation were employed to analyse the association between E2F1 and c-Myc transcripts in EBV^-^ B-cell, lung cancer, breast cancer, prostate cancer, and melanoma lines from DepMap portal (https://depmap.org/portal/). (B) Two-sided unpaired Student’s t-test and Pearson’s correlation were employed to analyse the association between E2F1 and c-Myc transcripts in DLBCL (diffuse large B-cell lymphoma), lung adenocarcinoma (LUAD), breast invasive carcinoma (BRCA), prostate adenocarcinoma (PRAD) and skin cutaneous melanoma (SKCM) patients’ tissue samples from TCGA datasets (https://ualcan.path.uab.edu/).
(TIF)

S14 Fig c-Myc does not regulate E2F1 transcription. (A) Luciferase reporter activity and the corresponding immunoblot analysis of the wild-type c-Myc promoter in the presence of increasing concentrations of E2F1 expression plasmid in transiently transfected HEK293 cells. (B) Luciferase reporter activity and the corresponding immunoblot analysis of the c-Myc promoter in the presence of empty vector, wild-type (WT) or transactivation domain deleted (ΔTAD) E2F1 expression plasmids in HEK293 cells. (C) Immunoblot analysis of HEK293T-BAC-GFP-EBV cells transiently transfected control vector or flag-tagged E2F1 expression plasmid. (D) Immunoblot analysis of HEK293T-BAC-GFP-EBV cells transiently transfected control vector or flag-tagged c-Myc expression plasmid. (E) Reanalysis of LCLs ChIP-Seq tracks (GSE30399 and GSE36354) of c-Myc at E2F1 promoter region. (F) Luciferase reporter activity and the corresponding immunoblot analysis of the wild-type E2F1 promoter in the presence of increasing concentrations of c-Myc expression plasmid in HEK293 cells. The results are presented as the mean ± SD, n = 3 biological replicates. Statistical significance was determined by a two-sided Student’s t-test, *P < 0.05; **P < 0.01; ***P < 0.001; ns, not significant.
(TIF)

S15 Fig BZLF1 does not regulate c-Myc transcription. (A) Heat map analysis (log2 Fold Change) of c-Myc transcript of RNA-Seq data (GSE237484) of LCL#1 and P3HR1 cells reactivated to lytic replication by TPA-NaBu treatment for 0–3 days post treatment (dpt). (B) Reanalysis of Raji ChIP-Seq tracks (E-MTAB-7788) of BZLF1 at c-Myc gene locus. (C) Luciferase reporter activity and the corresponding immunoblot analysis of the wild-type c-Myc promoter in the presence of increasing concentrations of BZLF1 expression plasmid in transiently transfected HEK293 cells. The results are presented as the mean + SD, n = 3 biological replicates. Statistical significance was determined by a two-sided Student’s t-test, *P < 0.05; **P < 0.01; ***P < 0.001; ns, not significant.
(TIF)

S1 Table Previously published datasets used in this study. (DOCX)

S2 Table E2F1 binding motifs located on different EBV Latent promoters. (DOCX)

S3 Table All E2F’s Structural and Sequence Similarity. (DOCX)

S4 Table RNA-Seq data of EBV transcripts from P3HR1 cells stably expressing E2F1 sh-RNA in the presence and absence of doxycycline and with or without TPA/sodium butyrate treatment. (XLSX)

S5 Table Differentially expressed cellular genes from P3HR1 cells stably expressing E2F1 sh-RNA in the presence and absence of doxycycline and either left untreated or treated with TPA/sodium butyrate. (XLSX)

S6 Table Oligo sequences used for qRT-PCR, cloning of Sh-RNAs, sgRNAs, cDNAs and promoter regions and ChIP-qPCR. (DOCX)

## References

[1] Saha A, Robertson ES. Mechanisms of B-Cell Oncogenesis Induced by Epstein-Barr Virus. J Virol. 2019;93(13):e00238-19. doi: 10.1128/JVI.00238-19 30971472 PMC6580952

[2] Young LS, Yap LF, Murray PG. Epstein-Barr virus: more than 50 years old and still providing surprises. Nat Rev Cancer. 2016;16(12):789–802. doi: 10.1038/nrc.2016.92 27687982

[3] Styles CT, Bazot Q, Parker GA, White RE, Paschos K, Allday MJ. EBV epigenetically suppresses the B cell-to-plasma cell differentiation pathway while establishing long-term latency. PLoS Biol. 2017;15(8):e2001992. doi: 10.1371/journal.pbio.2001992 28771465 PMC5542390

[4] Malik S, Biswas J, Sarkar P, Nag S, Gain C, Ghosh Roy S, et al. Differential carbonic anhydrase activities control EBV-induced B-cell transformation and lytic cycle reactivation. PLoS Pathog. 2024;20(3):e1011998. doi: 10.1371/journal.ppat.1011998 38530845 PMC10997083

[5] Shannon-Lowe C, Rickinson AB, Bell AI. Epstein-Barr virus-associated lymphomas. Philos Trans R Soc Lond B Biol Sci. 2017;372(1732):20160271. doi: 10.1098/rstb.2016.0271 28893938 PMC5597738

[6] Burton EM, Gewurz BE. Epstein-Barr virus oncoprotein-driven B cell metabolism remodeling. PLoS Pathog. 2022;18(2):e1010254. doi: 10.1371/journal.ppat.1010254 36264852 PMC9584359

[7] SoRelle ED, Reinoso-Vizcaino NM, Horn GQ, Luftig MA. Epstein-Barr virus perpetuates B cell germinal center dynamics and generation of autoimmune-associated phenotypes in vitro. Front Immunol. 2022;13:1001145. doi: 10.3389/fimmu.2022.1001145 36248899 PMC9554744

[8] Laichalk LL, Thorley-Lawson DA. Terminal differentiation into plasma cells initiates the replicative cycle of Epstein-Barr virus in vivo. J Virol. 2005;79(2):1296–307. doi: 10.1128/JVI.79.2.1296-1307.2005 15613356 PMC538585

[9] Yap LF, Wong AKC, Paterson IC, Young LS. Functional Implications of Epstein-Barr Virus Lytic Genes in Carcinogenesis. Cancers (Basel). 2022;14(23):5780. doi: 10.3390/cancers14235780 36497262 PMC9740547

[10] Wang Y, Yu J, Pei Y. Identifying the key regulators orchestrating Epstein-Barr virus reactivation. Front Microbiol. 2024;15:1505191. doi: 10.3389/fmicb.2024.1505191 39703703 PMC11655498

[11] Guo R, Jiang C, Zhang Y, Govande A, Trudeau SJ, Chen F, et al. MYC Controls the Epstein-Barr Virus Lytic Switch. Mol Cell. 2020;78(4):653-669.e8. doi: 10.1016/j.molcel.2020.03.025 32315601 PMC7245572

[12] Bernaudat F, Gustems M, Günther J, Oliva MF, Buschle A, Göbel C, et al. Structural basis of DNA methylation-dependent site selectivity of the Epstein-Barr virus lytic switch protein ZEBRA/Zta/BZLF1. Nucleic Acids Res. 2022;50(1):490–511. doi: 10.1093/nar/gkab1183 34893887 PMC8754650

[13] Albanese M, Tagawa T, Hammerschmidt W. Strategies of Epstein-Barr virus to evade innate antiviral immunity of its human host. Front Microbiol. 2022;13:955603. doi: 10.3389/fmicb.2022.955603 35935191 PMC9355577

[14] Yiu SPT, Dorothea M, Hui KF, Chiang AKS. Lytic Induction Therapy against Epstein-Barr Virus-Associated Malignancies: Past, Present, and Future. Cancers (Basel). 2020;12(8):2142. doi: 10.3390/cancers12082142 32748879 PMC7465660

[15] Meng Q, Hagemeier SR, Fingeroth JD, Gershburg E, Pagano JS, Kenney SC. The Epstein-Barr virus (EBV)-encoded protein kinase, EBV-PK, but not the thymidine kinase (EBV-TK), is required for ganciclovir and acyclovir inhibition of lytic viral production. J Virol. 2010;84(9):4534–42. doi: 10.1128/JVI.02487-09 20181711 PMC2863746

[16] Bhende PM, Dickerson SJ, Sun X, Feng W-H, Kenney SC. X-box-binding protein 1 activates lytic Epstein-Barr virus gene expression in combination with protein kinase D. J Virol. 2007;81(14):7363–70. doi: 10.1128/JVI.00154-07 17494074 PMC1933364

[17] Reusch JA, Nawandar DM, Wright KL, Kenney SC, Mertz JE. Cellular differentiation regulator BLIMP1 induces Epstein-Barr virus lytic reactivation in epithelial and B cells by activating transcription from both the R and Z promoters. J Virol. 2015;89(3):1731–43. doi: 10.1128/JVI.02781-14 25410866 PMC4300755

[18] Gain C, Malik S, Bhattacharjee S, Ghosh A, Robertson ES, Das BB, et al. Proteasomal inhibition triggers viral oncoprotein degradation via autophagy-lysosomal pathway. PLoS Pathog. 2020;16(2):e1008105. doi: 10.1371/journal.ppat.1008105 32092124 PMC7058366

[19] Choi E-H, Kim KP. E2F1 facilitates DNA break repair by localizing to break sites and enhancing the expression of homologous recombination factors. Exp Mol Med. 2019;51(9):1–12. doi: 10.1038/s12276-019-0307-2 31534120 PMC6802646

[20] E X, Pickering MT, Debatis M, Castillo J, Lagadinos A, Wang S, et al. An E2F1-mediated DNA damage response contributes to the replication of human cytomegalovirus. PLoS Pathog. 2011;7(5):e1001342. doi: 10.1371/journal.ppat.1001342 21589897 PMC3093362

[21] Johnson L, Shen A, Boyle L, Kunich J, Pandey K, Lemmon M, et al. Selectively replicating adenoviruses targeting deregulated E2F activity are potent, systemic antitumor agents. Cancer Cell. 2002;1(4):325–37. doi: 10.1016/s1535-6108(02)00060-0 12086848

[22] Saha A, Lu J, Morizur L, Upadhyay SK, Aj MP, Robertson ES. E2F1 mediated apoptosis induced by the DNA damage response is blocked by EBV nuclear antigen 3C in lymphoblastoid cells. PLoS Pathog. 2012;8(3):e1002573. doi: 10.1371/journal.ppat.1002573 22438805 PMC3305458

[23] Boreström C, Forsman A, Rüetschi U, Rymo L. E2F1, ARID3A/Bright and Oct-2 factors bind to the Epstein-Barr virus C promoter, EBNA1 and oriP, participating in long-distance promoter-enhancer interactions. J Gen Virol. 2012;93(Pt 5):1065–75. doi: 10.1099/vir.0.038752-0 22302879

[24] Pei Y, Banerjee S, Sun Z, Jha HC, Saha A, Robertson ES. EBV Nuclear Antigen 3C Mediates Regulation of E2F6 to Inhibit E2F1 Transcription and Promote Cell Proliferation. PLoS Pathog. 2016;12(8):e1005844. doi: 10.1371/journal.ppat.1005844 27548379 PMC4993364

[25] Mauser A, Holley-Guthrie E, Zanation A, Yarborough W, Kaufmann W, Klingelhutz A, et al. The Epstein-Barr virus immediate-early protein BZLF1 induces expression of E2F-1 and other proteins involved in cell cycle progression in primary keratinocytes and gastric carcinoma cells. J Virol. 2002;76(24):12543–52. doi: 10.1128/jvi.76.24.12543-12552.2002 12438580 PMC136734

[26] Lin Z, Yin Q, Flemington E. Identification of a negative regulatory element in the Epstein-Barr virus Zta transactivation domain that is regulated by the cell cycle control factors c-Myc and E2F1. J Virol. 2004;78(21):11962–71. doi: 10.1128/JVI.78.21.11962-11971.2004 15479836 PMC523277

[27] Kumari A, Iwasaki T, Pyndiah S, Cassimere EK, Palani CD, Sakamuro D. Regulation of E2F1-induced apoptosis by poly(ADP-ribosyl)ation. Cell Death Differ. 2015;22(2):311–22. doi: 10.1038/cdd.2014.146 25257171 PMC4291492

[28] Shats I, Deng M, Davidovich A, Zhang C, Kwon JS, Manandhar D, et al. Expression level is a key determinant of E2F1-mediated cell fate. Cell Death Differ. 2017;24(4):626–37. doi: 10.1038/cdd.2017.12 28211871 PMC5384025

[29] White RE, Groves IJ, Turro E, Yee J, Kremmer E, Allday MJ. Extensive co-operation between the Epstein-Barr virus EBNA3 proteins in the manipulation of host gene expression and epigenetic chromatin modification. PLoS One. 2010;5(11):e13979. doi: 10.1371/journal.pone.0013979 21085583 PMC2981562

[30] Zhang K, Lv D-W, Li R. B Cell Receptor Activation and Chemical Induction Trigger Caspase-Mediated Cleavage of PIAS1 to Facilitate Epstein-Barr Virus Reactivation. Cell Rep. 2017;21(12):3445–57. doi: 10.1016/j.celrep.2017.11.071 29262325 PMC5741098

[31] SoRelle ED, Haynes LE, Willard KA, Chang B, Ch’ng J, Christofk H, et al. Epstein-Barr virus reactivation induces divergent abortive, reprogrammed, and host shutoff states by lytic progression. PLoS Pathog. 2024;20(10):e1012341. doi: 10.1371/journal.ppat.1012341 39446925 PMC11563402

[32] Lavorgna A, Harhaj EW. EBV LMP1: New and shared pathways to NF-κB activation. Proc Natl Acad Sci U S A. 2012;109(7):2188–9. doi: 10.1073/pnas.1121357109 22308477 PMC3289357

[33] Buschle A, Mrozek-Gorska P, Cernilogar FM, Ettinger A, Pich D, Krebs S, et al. Epstein-Barr virus inactivates the transcriptome and disrupts the chromatin architecture of its host cell in the first phase of lytic reactivation. Nucleic Acids Res. 2021;49(6):3217–41. doi: 10.1093/nar/gkab099 33675667 PMC8034645

[34] Rauluseviciute I, Riudavets-Puig R, Blanc-Mathieu R, Castro-Mondragon JA, Ferenc K, Kumar V, et al. JASPAR 2024: 20th anniversary of the open-access database of transcription factor binding profiles. Nucleic Acids Res. 2024;52(D1):D174–82. doi: 10.1093/nar/gkad1059 37962376 PMC10767809

[35] Yu X, McCarthy PJ, Lim H-J, Iempridee T, Kraus RJ, Gorlen DA, et al. The ZIIR element of the Epstein-Barr virus BZLF1 promoter plays a central role in establishment and maintenance of viral latency. J Virol. 2011;85(10):5081–90. doi: 10.1128/JVI.02615-10 21389123 PMC3126197

[36] Yockteng-Melgar J, Shire K, Cheng AZ, Malik-Soni N, Harris RS, Frappier L. G1/S Cell Cycle Induction by Epstein-Barr Virus BORF2 Is Mediated by P53 and APOBEC3B. J Virol. 2022;96(18):e0066022. doi: 10.1128/jvi.00660-22 36069545 PMC9517719

[37] Rodriguez A, Armstrong M, Dwyer D, Flemington E. Genetic dissection of cell growth arrest functions mediated by the Epstein-Barr virus lytic gene product, Zta. J Virol. 1999;73(11):9029–38. doi: 10.1128/JVI.73.11.9029-9038.1999 10516009 PMC112935

[38] Huang S-Y, Hsieh M-J, Chen C-Y, Chen Y-J, Chen J-Y, Chen M-R, et al. Epstein-Barr virus Rta-mediated transactivation of p21 and 14-3-3σ arrests cells at the G1/S transition by reducing cyclin E/CDK2 activity. J Gen Virol. 2012;93(Pt 1):139–49. doi: 10.1099/vir.0.034405-0 21918011

[39] Paladino P, Marcon E, Greenblatt J, Frappier L. Identification of herpesvirus proteins that contribute to G1/S arrest. J Virol. 2014;88(8):4480–92. doi: 10.1128/JVI.00059-14 24501404 PMC3993752

[40] Li C, Romero-Masters JC, Huebner S, Ohashi M, Hayes M, Bristol JA, et al. EBNA2-deleted Epstein-Barr virus (EBV) isolate, P3HR1, causes Hodgkin-like lymphomas and diffuse large B cell lymphomas with type II and Wp-restricted latency types in humanized mice. PLoS Pathog. 2020;16(6):e1008590. doi: 10.1371/journal.ppat.1008590 32542010 PMC7316346

[41] Murata T, Sugimoto A, Inagaki T, Yanagi Y, Watanabe T, Sato Y. Molecular basis of Epstein-Barr virus latency establishment and lytic reactivation. Viruses. 2021;13:2344. 34960613 10.3390/v13122344 PMC8706188

[42] Coller HA, Forman JJ, Legesse-Miller A. “Myc’ed messages”: myc induces transcription of E2F1 while inhibiting its translation via a microRNA polycistron. PLoS Genet. 2007;3(8):e146. doi: 10.1371/journal.pgen.0030146 17784791 PMC1959363

[43] Fernandez PC, Frank SR, Wang L, Schroeder M, Liu S, Greene J, et al. Genomic targets of the human c-Myc protein. Genes Dev. 2003;17(9):1115–29. doi: 10.1101/gad.1067003 12695333 PMC196049

[44] Ladu S, Calvisi DF, Conner EA, Farina M, Factor VM, Thorgeirsson SS. E2F1 inhibits c-Myc-driven apoptosis via PIK3CA/Akt/mTOR and COX-2 in a mouse model of human liver cancer. Gastroenterology. 2008;135(4):1322–32. doi: 10.1053/j.gastro.2008.07.012 18722373 PMC2614075

[45] Zhang Y, Zhang A, Shen C, Zhang B, Rao Z, Wang R, et al. E2F1 acts as a negative feedback regulator of c-Myc‑induced hTERT transcription during tumorigenesis. Oncol Rep. 2014;32(3):1273–80. doi: 10.3892/or.2014.3287 24969314

[46] Leung JY, Ehmann GL, Giangrande PH, Nevins JR. A role for Myc in facilitating transcription activation by E2F1. Oncogene. 2008;27(30):4172–9. doi: 10.1038/onc.2008.55 18345030

[47] Matsumura I, Tanaka H, Kanakura Y. E2F1 and c-Myc in cell growth and death. Cell Cycle. 2003;2(4):333–8. 12851485

[48] Elliott MJ, Dong YB, Yang H, McMasters KM. E2F-1 up-regulates c-Myc and p14(ARF) and induces apoptosis in colon cancer cells. Clin Cancer Res. 2001;7(11):3590–7. 11705881

[49] Dorothea M, Xie J, Yiu SPT, Chiang AKS. Contribution of Epstein-Barr Virus Lytic Proteins to Cancer Hallmarks and Implications from Other Oncoviruses. Cancers (Basel). 2023;15(7):2120. doi: 10.3390/cancers15072120 37046781 PMC10093119

[50] Hong GK, Gulley ML, Feng W-H, Delecluse H-J, Holley-Guthrie E, Kenney SC. Epstein-Barr virus lytic infection contributes to lymphoproliferative disease in a SCID mouse model. J Virol. 2005;79(22):13993–4003. doi: 10.1128/JVI.79.22.13993-14003.2005 16254335 PMC1280209

[51] DeCaprio JA, Ludlow JW, Figge J, Shew JY, Huang CM, Lee WH, et al. SV40 large tumor antigen forms a specific complex with the product of the retinoblastoma susceptibility gene. Cell. 1988;54(2):275–83. doi: 10.1016/0092-8674(88)90559-4 2839300

[52] Mal A, Piotrkowski A, Harter ML. Cyclin-dependent kinases phosphorylate the adenovirus E1A protein, enhancing its ability to bind pRb and disrupt pRb-E2F complexes. J Virol. 1996;70(5):2911–21. doi: 10.1128/JVI.70.5.2911-2921.1996 8627766 PMC190149

[53] Hwang SG, Lee D, Kim J, Seo T, Choe J. Human papillomavirus type 16 E7 binds to E2F1 and activates E2F1-driven transcription in a retinoblastoma protein-independent manner. J Biol Chem. 2002;277(4):2923–30. doi: 10.1074/jbc.M109113200 11713253

[54] Knight JS, Sharma N, Robertson ES. Epstein-Barr virus latent antigen 3C can mediate the degradation of the retinoblastoma protein through an SCF cellular ubiquitin ligase. Proc Natl Acad Sci U S A. 2005;102(51):18562–6. doi: 10.1073/pnas.0503886102 16352731 PMC1317900

[55] Gnanasundram SV, Pyndiah S, Daskalogianni C, Armfield K, Nylander K, Wilson JB, et al. PI3Kδ activates E2F1 synthesis in response to mRNA translation stress. Nat Commun. 2017;8(1):2103. doi: 10.1038/s41467-017-02282-w 29235459 PMC5727396

[56] Gaillard H, García-Muse T, Aguilera A. Replication stress and cancer. Nat Rev Cancer. 2015;15(5):276–89. doi: 10.1038/nrc3916 25907220

[57] Nikitin PA, Yan CM, Forte E, Bocedi A, Tourigny JP, White RE, et al. An ATM/Chk2-mediated DNA damage-responsive signaling pathway suppresses Epstein-Barr virus transformation of primary human B cells. Cell Host Microbe. 2010;8(6):510–22. doi: 10.1016/j.chom.2010.11.004 21147465 PMC3049316

[58] Bhende PM, Seaman WT, Delecluse H-J, Kenney SC. The EBV lytic switch protein, Z, preferentially binds to and activates the methylated viral genome. Nat Genet. 2004;36(10):1099–104. doi: 10.1038/ng1424 15361873

[59] Bergbauer M, Kalla M, Schmeinck A, Göbel C, Rothbauer U, Eck S, et al. CpG-methylation regulates a class of Epstein-Barr virus promoters. PLoS Pathog. 2010;6(9):e1001114. doi: 10.1371/journal.ppat.1001114 20886097 PMC2944802

[60] Wille CK, Nawandar DM, Panfil AR, Ko MM, Hagemeier SR, Kenney SC. Viral genome methylation differentially affects the ability of BZLF1 versus BRLF1 to activate Epstein-Barr virus lytic gene expression and viral replication. J Virol. 2013;87(2):935–50. doi: 10.1128/JVI.01790-12 23135711 PMC3554042

[61] Li L, Su X, Choi GCG, Cao Y, Ambinder RF, Tao Q. Methylation profiling of Epstein-Barr virus immediate-early gene promoters, BZLF1 and BRLF1 in tumors of epithelial, NK- and B-cell origins. BMC Cancer. 2012;12:125. doi: 10.1186/1471-2407-12-125 22458933 PMC3362778

[62] Murata T, Kondo Y, Sugimoto A, Kawashima D, Saito S, Isomura H, et al. Epigenetic histone modification of Epstein-Barr virus BZLF1 promoter during latency and reactivation in Raji cells. J Virol. 2012;86(9):4752–61. doi: 10.1128/JVI.06768-11 22357272 PMC3347330

[63] Guo R, Zhang Y, Teng M, Jiang C, Schineller M, Zhao B, et al. DNA methylation enzymes and PRC1 restrict B-cell Epstein-Barr virus oncoprotein expression. Nat Microbiol. 2020;5(8):1051–63. doi: 10.1038/s41564-020-0724-y 32424339 PMC7462085

[64] Nishiyama A, Mulholland CB, Bultmann S, Kori S, Endo A, Saeki Y, et al. Two distinct modes of DNMT1 recruitment ensure stable maintenance DNA methylation. Nat Commun. 2020;11(1):1222. doi: 10.1038/s41467-020-15006-4 32144273 PMC7060239

[65] Benavente CA, Finkelstein D, Johnson DA, Marine J-C, Ashery-Padan R, Dyer MA. Chromatin remodelers HELLS and UHRF1 mediate the epigenetic deregulation of genes that drive retinoblastoma tumor progression. Oncotarget. 2014;5(20):9594–608. doi: 10.18632/oncotarget.2468 25338120 PMC4259422

[66] McCabe MT, Davis JN, Day ML. Regulation of DNA methyltransferase 1 by the pRb/E2F1 pathway. Cancer Res. 2005;65(9):3624–32. doi: 10.1158/0008-5472.CAN-04-2158 15867357

[67] Li J, Wang R, Hu X, Gao Y, Wang Z, Li J, et al. Activated MEK/ERK Pathway Drives Widespread and Coordinated Overexpression of UHRF1 and DNMT1 in Cancer cells. Sci Rep. 2019;9(1):907. doi: 10.1038/s41598-018-37258-3 30696879 PMC6351616

[68] Chang L, Campbell J, Raji IO, Guduru SKR, Kandel P, Nguyen M, et al. Discovery of small molecules targeting the tandem tudor domain of the epigenetic factor UHRF1 using fragment-based ligand discovery. Sci Rep. 2021;11(1):1121. doi: 10.1038/s41598-020-80588-4 33441849 PMC7806715

[69] Shaffer AL, Lin KI, Kuo TC, Yu X, Hurt EM, Rosenwald A, et al. Blimp-1 orchestrates plasma cell differentiation by extinguishing the mature B cell gene expression program. Immunity. 2002;17(1):51–62. doi: 10.1016/s1074-7613(02)00335-7 12150891

[70] Obiedat A, Seidel E, Mahameed M, Berhani O, Tsukerman P, Voutetakis K, et al. Transcription of the NKG2D ligand MICA is suppressed by the IRE1/XBP1 pathway of the unfolded protein response through the regulation of E2F1. FASEB J. 2019;33(3):3481–95. doi: 10.1096/fj.201801350RR 30452881

[71] Balan N, Osborn K, Sinclair AJ. Repression of CIITA by the Epstein-Barr virus transcription factor Zta is independent of its dimerization and DNA binding. J Gen Virol. 2016;97(3):725–32. doi: 10.1099/jgv.0.000369 26653871 PMC5381392

[72] Ramasubramanyan S, Osborn K, Al-Mohammad R, Naranjo Perez-Fernandez IB, Zuo J, Balan N, et al. Epstein-Barr virus transcription factor Zta acts through distal regulatory elements to directly control cellular gene expression. Nucleic Acids Res. 2015;43(7):3563–77. doi: 10.1093/nar/gkv212 25779048 PMC4402532

[73] Germini D, Sall FB, Shmakova A, Wiels J, Dokudovskaya S, Drouet E, et al. Oncogenic Properties of the EBV ZEBRA Protein. Cancers (Basel). 2020;12(6):1479. doi: 10.3390/cancers12061479 32517128 PMC7352903

[74] Bristol JA, Robinson AR, Barlow EA, Kenney SC. The Epstein-Barr virus BZLF1 protein inhibits tumor necrosis factor receptor 1 expression through effects on cellular C/EBP proteins. J Virol. 2010;84(23):12362–74. doi: 10.1128/JVI.00712-10 20861254 PMC2976414

[75] Wang LW, Shen H, Nobre L, Ersing I, Paulo JA, Trudeau S. Epstein-Barr-virus-induced one-carbon metabolism drives B cell transformation. Cell Metab. 2019;30:539-555.e11. 31257153 10.1016/j.cmet.2019.06.003 PMC6720460

[76] Zhu J, Liao G, Shan L, Zhang J, Chen M-R, Hayward GS, et al. Protein array identification of substrates of the Epstein-Barr virus protein kinase BGLF4. J Virol. 2009;83(10):5219–31. doi: 10.1128/JVI.02378-08 19244323 PMC2682057

[77] Sato Y, Watanabe T, Suzuki C, Abe Y, Masud HMAA, Inagaki T, et al. S-Like-Phase Cyclin-Dependent Kinases Stabilize the Epstein-Barr Virus BDLF4 Protein To Temporally Control Late Gene Transcription. J Virol. 2019;93(8):e01707-18. doi: 10.1128/JVI.01707-18 30700607 PMC6450117

[78] Tan H, Gong Y, Liu Y, Long J, Luo Q, Faleti OD, et al. Advancing therapeutic strategies for Epstein-Barr virus-associated malignancies through lytic reactivation. Biomed Pharmacother. 2023;164:114916. doi: 10.1016/j.biopha.2023.114916 37229802

[79] Halder S, Murakami M, Verma SC, Kumar P, Yi F, Robertson ES. Early events associated with infection of Epstein-Barr virus infection of primary B-cells. PLoS One. 2009;4(9):e7214. doi: 10.1371/journal.pone.0007214 19784370 PMC2746279

[80] Ryan JL, Fan H, Glaser SL, Schichman SA, Raab-Traub N, Gulley ML. Epstein-Barr virus quantitation by real-time PCR targeting multiple gene segments: a novel approach to screen for the virus in paraffin-embedded tissue and plasma. J Mol Diagn. 2004;6(4):378–85. doi: 10.1016/S1525-1578(10)60535-1 15507678 PMC1867486

[81] Wang B, Ma A, Zhang L, Jin W-L, Qian Y, Xu G, et al. POH1 deubiquitylates and stabilizes E2F1 to promote tumour formation. Nat Commun. 2015;6:8704. doi: 10.1038/ncomms9704 26510456 PMC4846323

[82] Varadi M, Bertoni D, Magana P, Paramval U, Pidruchna I, Radhakrishnan M, et al. AlphaFold Protein Structure Database in 2024: providing structure coverage for over 214 million protein sequences. Nucleic Acids Res. 2024;52(D1):D368–75. doi: 10.1093/nar/gkad1011 37933859 PMC10767828

