# Supplementary figures and images for: E2F1 suppresses Epstein-Barr virus lytic reactivation through cellular and viral transcriptional networks
Source: PLoS Pathog. 2025 Aug 7;21(8):e1013410. doi: 10.1371/journal.ppat.1013410 (PMC12349880; doi:10.1371/journal.ppat.1013410)

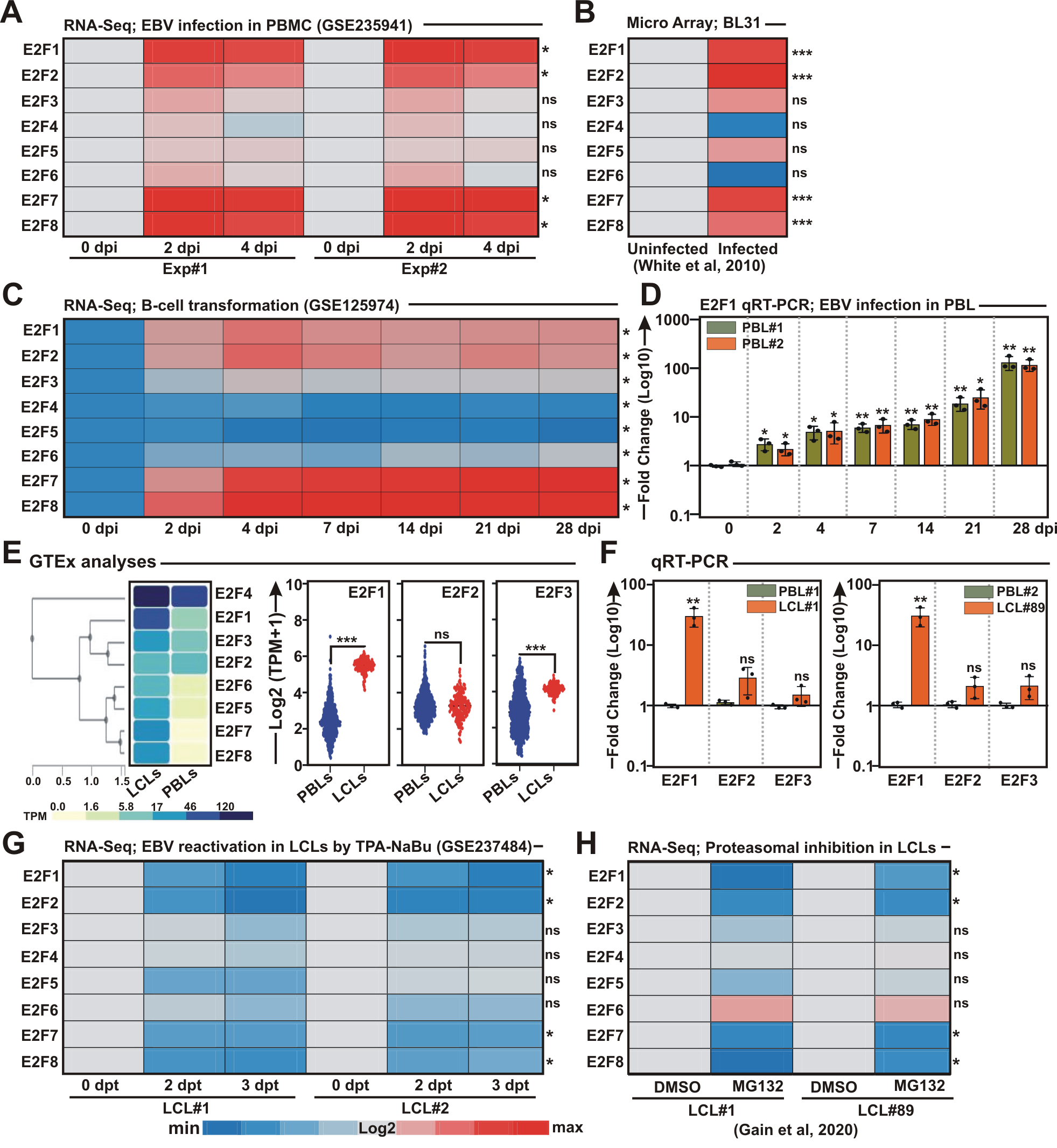

Supplement: S1 Fig — (A) Heat map analysis (log2 Fold Change) of all eight E2F genes (E2F1-8) of RNA-Seq data (GSE235941) of peripheral blood mononuclear cells (PBMCs) infected with GFP-EBV for 0–4 days post-infection (dpi). (B) Heat map representation of reanalysis of microarray data [29] of E2F transcripts (E2F1-8) in uninfected and EBV infected BL31 cells. (C) Heat map representation of differential gene expression of the E2F genes (E2F1-8) of RNA-Seq data (GSE125974) of B-cells infected with EBV for 0–28 dpi. (D) qRT-PCR analysis of cDNA generated from PBMCs from two individual donors infected with GFP-EBV for 0–28 dpi. (E) Heat map and dot plot analysis of the transcripts profile of the indicated E2F genes in whole blood cells (PBLs) and EBV transformed lymphoblastoid cell lines (LCLs) using ‘Genotype-Tissue Expression (GTEx)’ project. (F) qRT-PCR analysis of cDNA generated from PBMCs from two individual donors and two LCLs – LCL#1 and LCL#89. (G) Heat map representation of differential gene expression of the E2F genes (E2F1-8) of RNA-Seq data (GSE237484) of two LCLs (LCL#1 and LCL#89) reactivated to lytic replication by TPA-NaBu treatment for 0–3 days post treatment (dpt). (H) Heat map representation of differential gene expression of the E2F genes (E2F1-8) of RNA-Seq data [18] of two LCLs (LCL#1 and LCL#89) either left untreated or treated with 1 µM MG132 for 24 h. qRT-PCR results are presented as the mean ± SD, n = 3 biological replicates. Statistical significance was determined by a two-sided Student’s t-test, *P < 0.05; **P < 0.01; ***P < 0.001; ns, not significant. (TIF) [file ppat.1013410.s001.TIF]

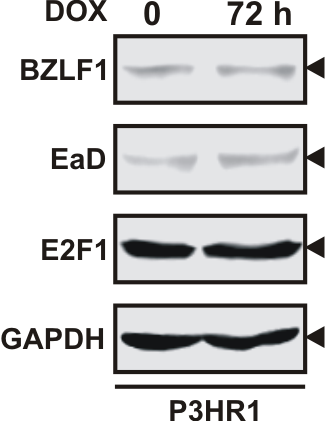

Supplement: S2 Fig — P3HR1 cells were subjected to immunoblot analysis without or with doxycycline (-/ + DOX) treatment for 72 h. (TIF) [file ppat.1013410.s002.TIF]

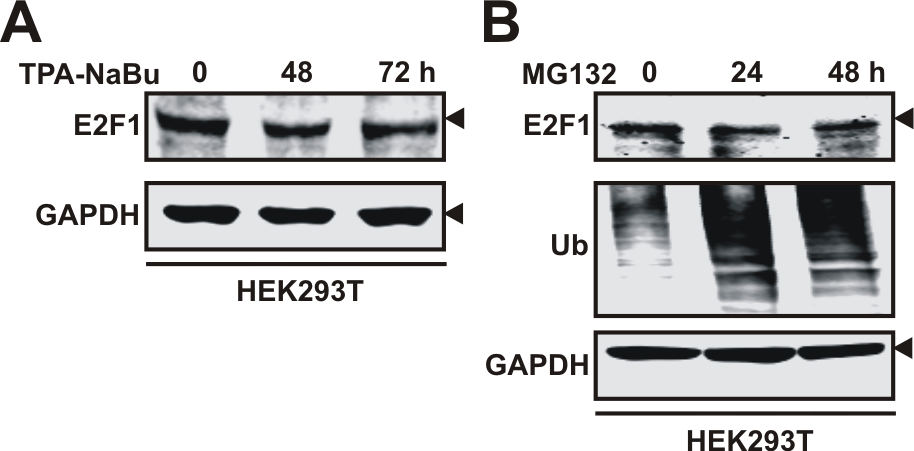

Supplement: S3 Fig — (A) HEK293 cells were treated with 20 ng/ml TPA and 3 mM sodium NaBu treatment for the indicated time points (0–72 h) and subjected to immunoblot analysis. (B) HEK293 cells were treated with 1 μM MG132 for the indicated time points (0–48 h) and subjected to immunoblot analysis. (TIF) [file ppat.1013410.s003.TIF]

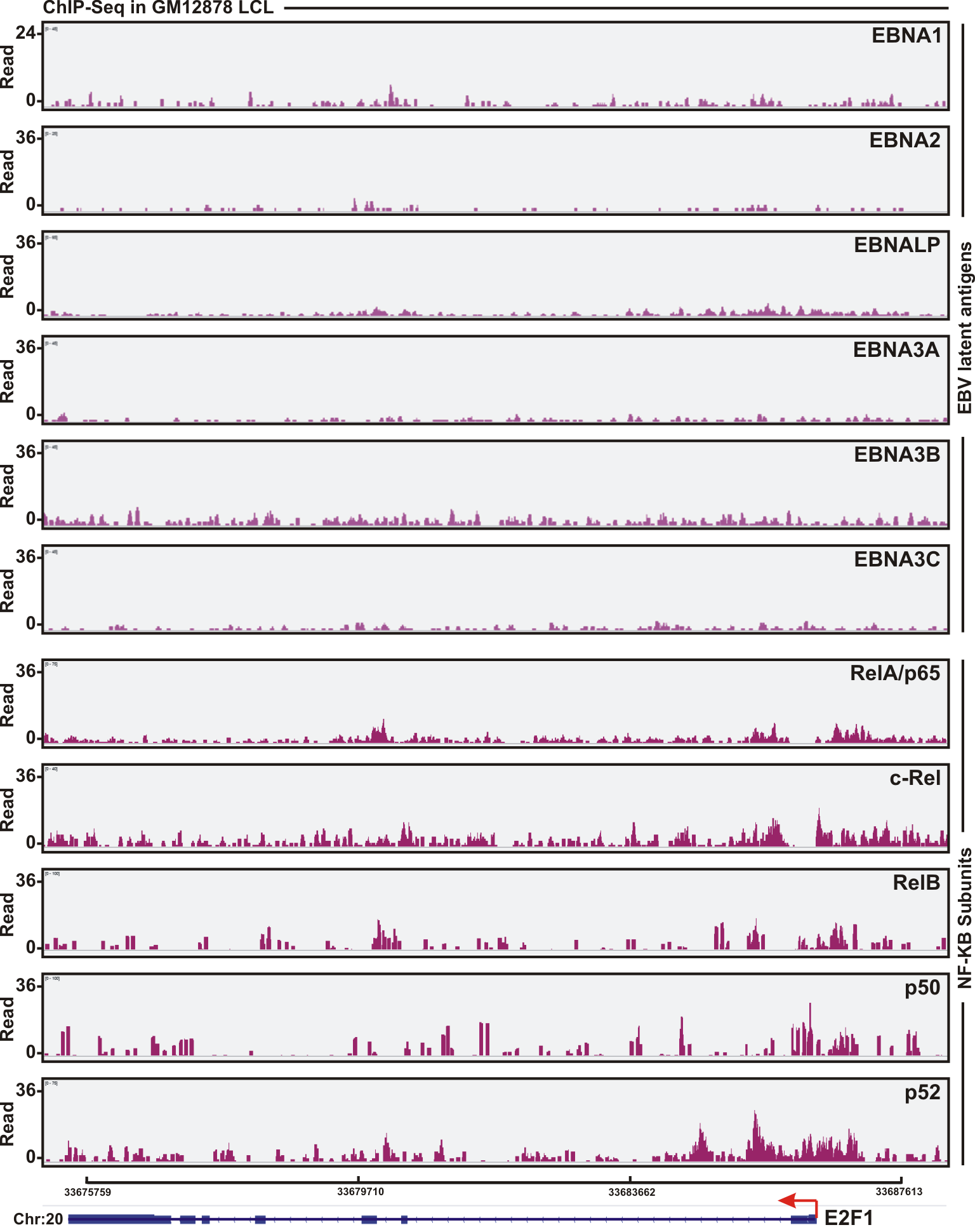

Supplement: S4 Fig — ChIP-Seq tracks for EBV oncoproteins - EBNA1 (GSE73887), EBNA2 (GSE29498), EBNALP (GSE49338), EBNA3A (GSE88729), EBNA3B (GSE88729), EBNA3C (GSE88729), and NF-ĸB subunits (GSE55105) - RelA/p65, c-Rel, RelB, p50, p52 at E2F1 promoter region. (TIF) [file ppat.1013410.s004.TIF]

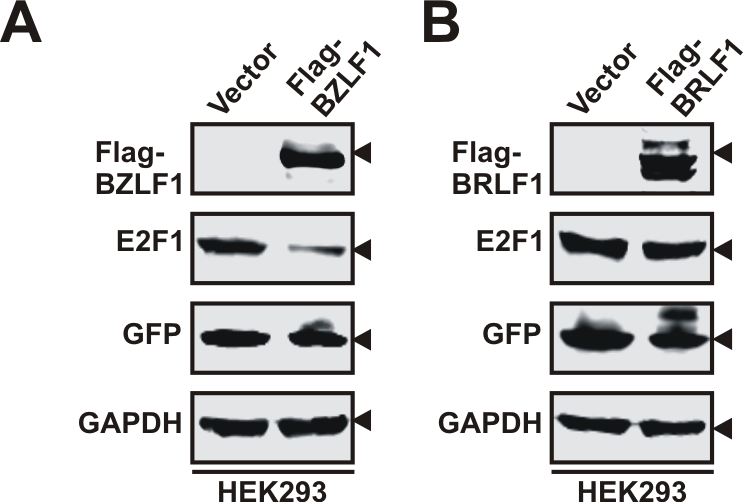

Supplement: S5 Fig — (A) Immunoblot analysis of HEK293 cells transiently transfected with flag-tagged BZLF1 expression plasmid. (B) Immunoblot analysis of HEK293 cells transiently transfected with flag-tagged BRLF1 expression plasmid. (TIF) [file ppat.1013410.s005.TIF]

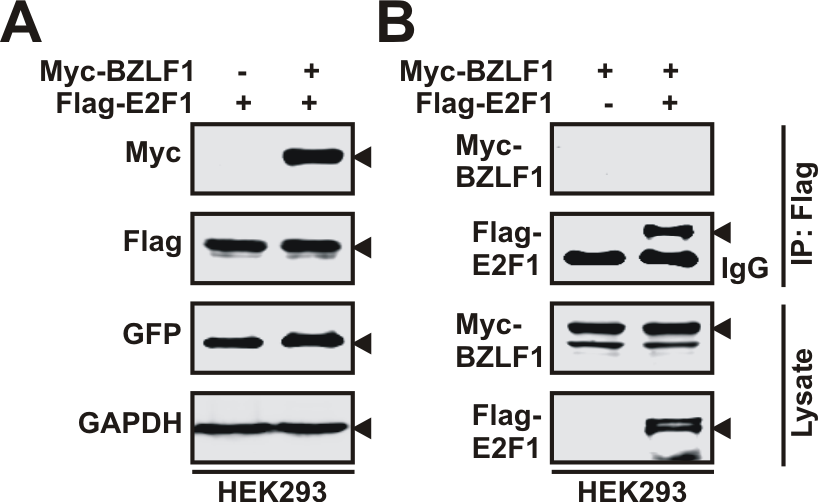

Supplement: S6 Fig — (A) Immunoblot analysis of HEK293 cells transiently transfected with flag-tagged E2F1 expression plasmid in the presence of either control vector or myc-tagged BZLF1 expression plasmid. (B) HEK293 cells transiently transfected myc-tagged BZLF1 expression plasmid with or without flag-tagged E2F1 expression plasmid were subjected to co-immunoprecipitation analysis using anti-flag antibody. (TIF) [file ppat.1013410.s006.TIF]

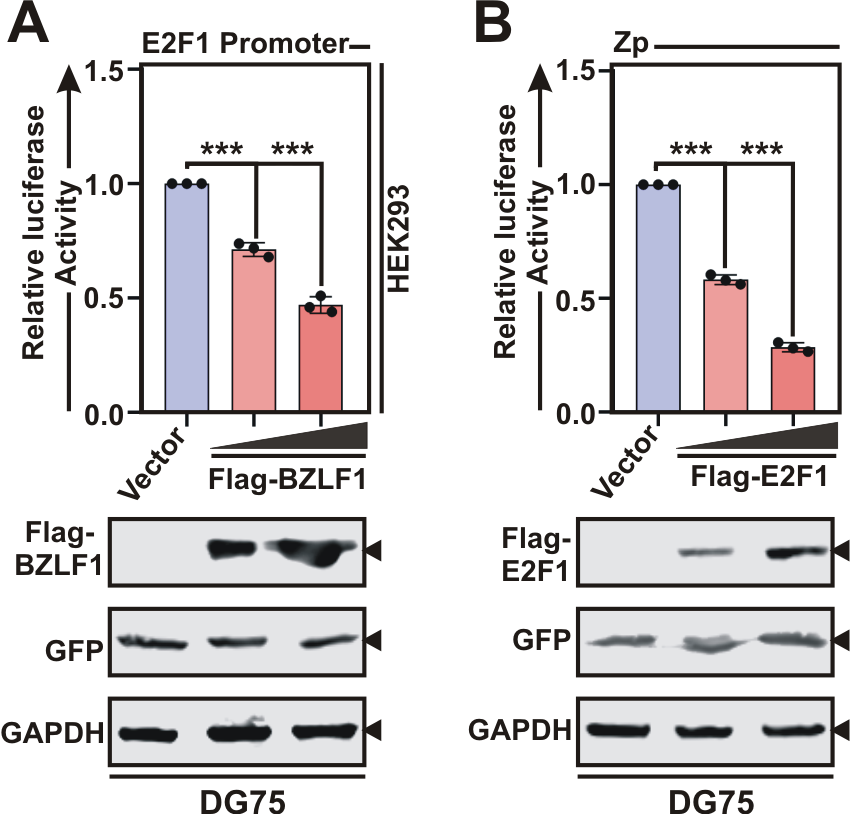

Supplement: S7 Fig — (A) Luciferase reporter activity and the corresponding immunoblot analysis of the wild-type E2F1 promoter in the presence of increasing concentrations of BZLF1 expression plasmid in transiently transfected EBV- DG75 cells. (B) Luciferase reporter activity and the corresponding immunoblot analysis of the wild-type BZLF1 promoter (Zp) in the presence of increasing concentrations of E2F1 expression plasmid in transiently transfected EBV- DG75 cells. (TIF) [file ppat.1013410.s007.TIF]

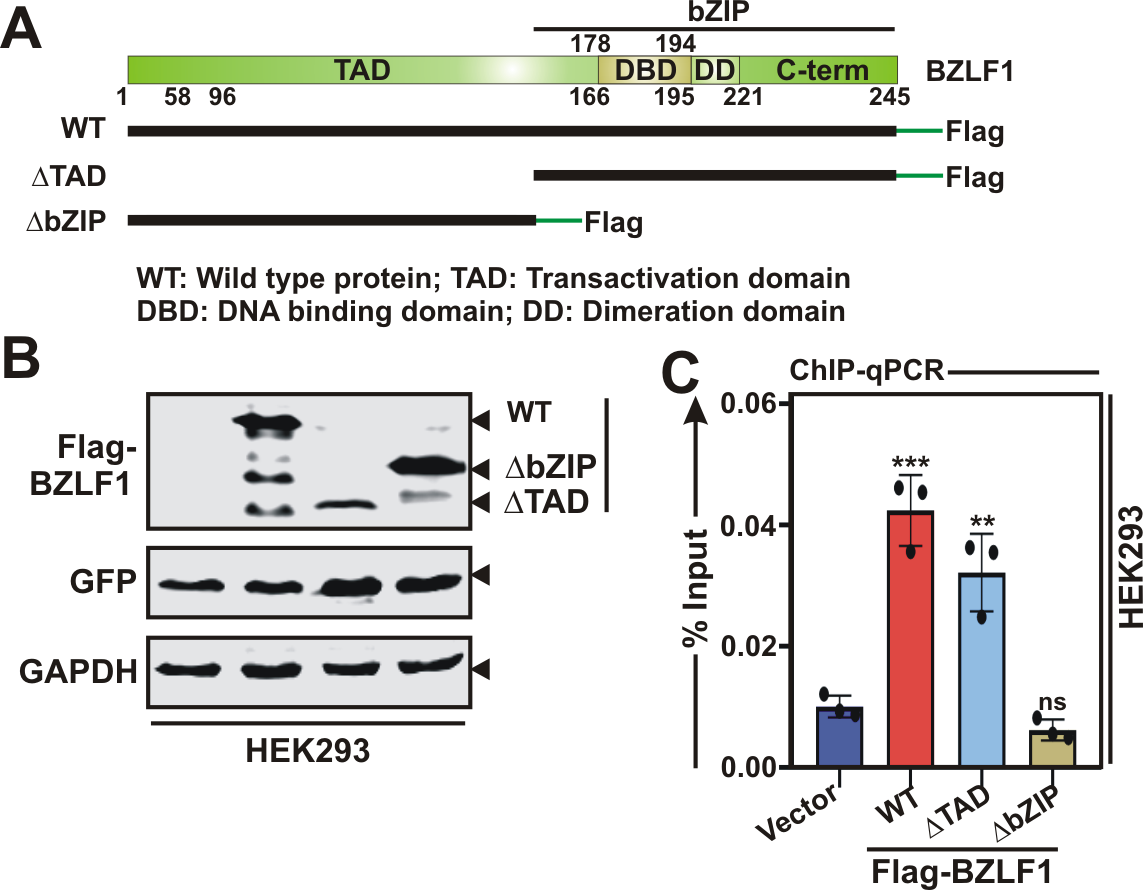

Supplement: S8 Fig — (A) Schematic showing different structural domains of BZLF1 for cloning in a flag-tagged expression vector. (B) Immunoblot analysis of HEK293 cells transiently transfected with control vector or flag-tagged expression plasmids for wild-type (WT), ΔTAD and ΔbZIP BZLF1 proteins. (C) ChIP-qPCR data showing recruitment of flag-tagged WT and ΔTAD BZLF1 proteins at E2F1 promoter region in transiently transfected HEK293 cells. The results are presented as the mean + SD, n = 3 biological replicates. Statistical significance was determined by a two-sided Student’s t-test, *P < 0.05; **P < 0.01; ***P < 0.001; ns, not significant. (TIF) [file ppat.1013410.s008.TIF]

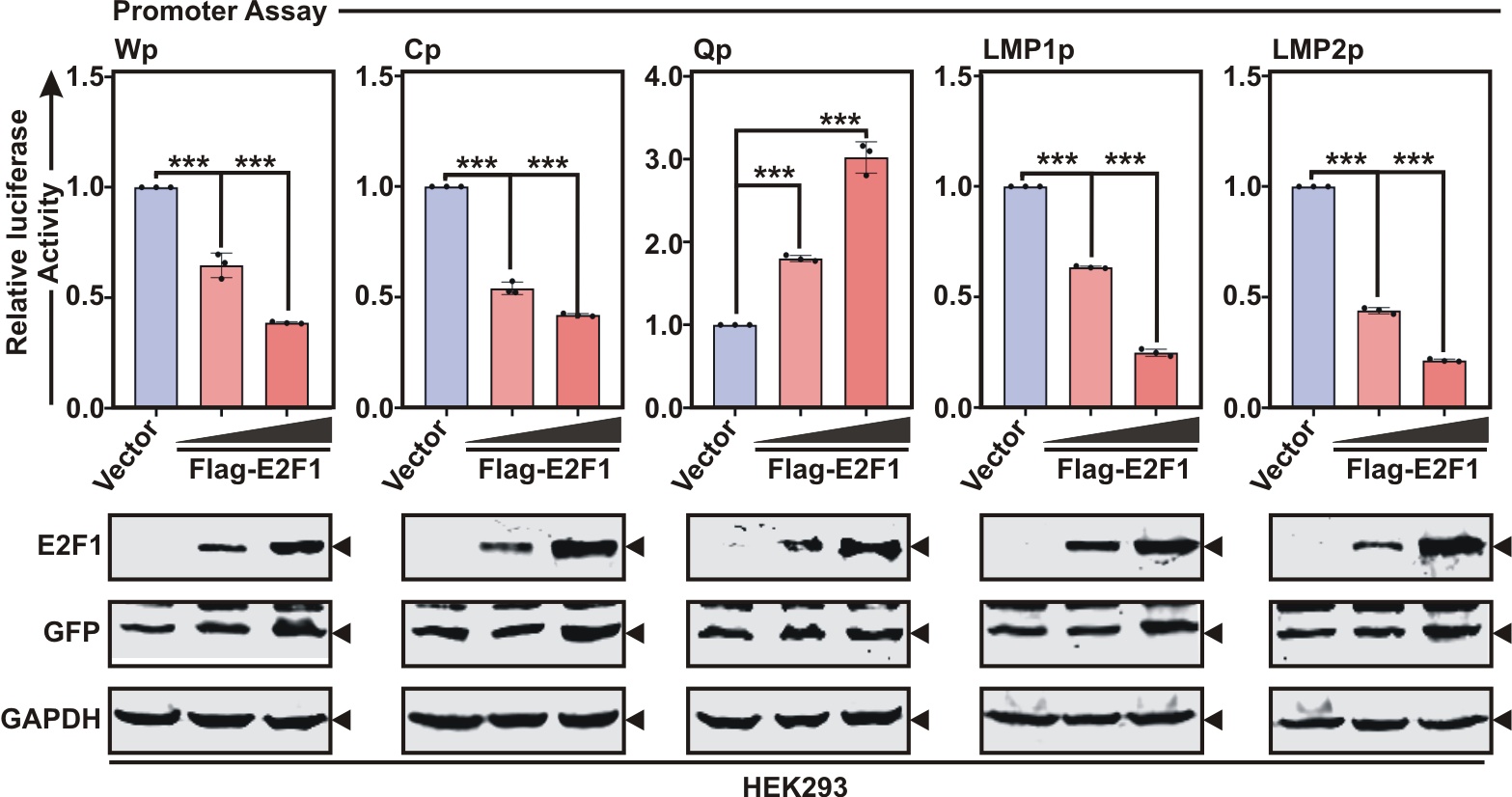

Supplement: S9 Fig — Luciferase reporter activity and the corresponding immunoblot analysis of different EBV latent promoters – Wp, Cp, Qp, LMP1p and LMP2p in the presence of increasing concentrations of E2F1. All the experiments were performed in HEK293 cells. The results are presented as the mean ± SD, n = 3 biological replicates. Statistical significance was determined by a two-sided Student’s t-test, *P < 0.05; **P < 0.01; ***P < 0.001; ns, not significant. (TIF) [file ppat.1013410.s009.TIF]

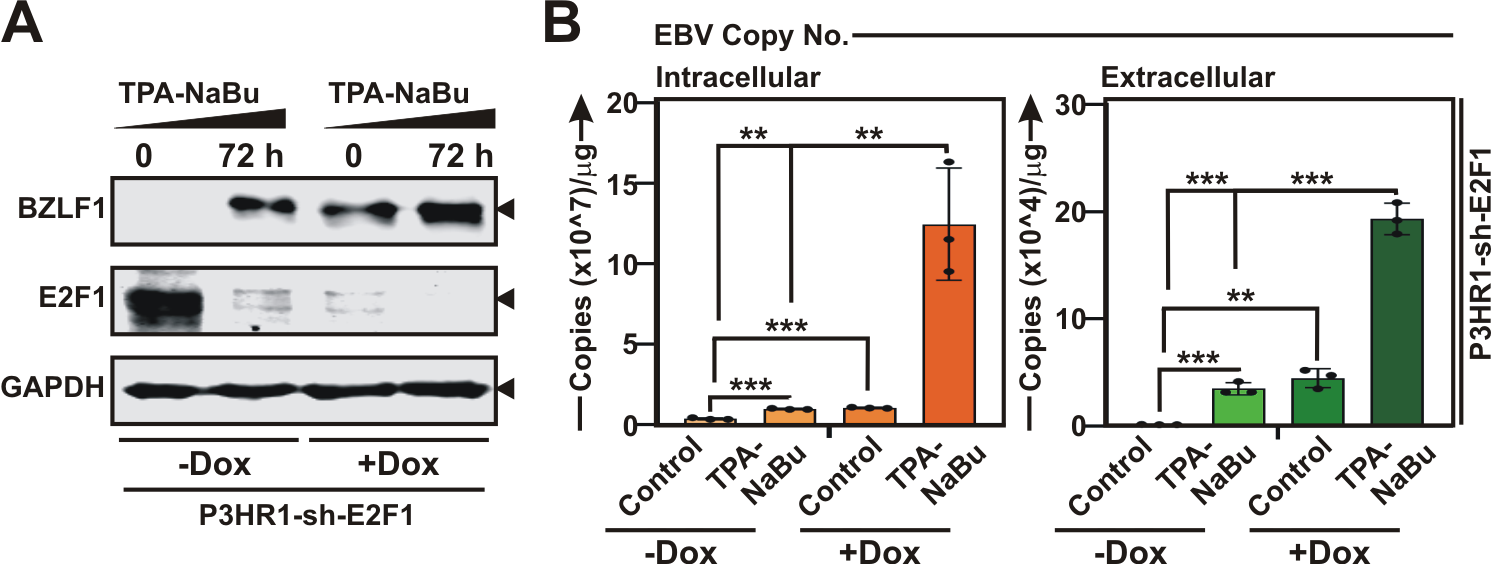

Supplement: S10 Fig — (A) Immunoblot analysis of P3HR1 cells stably expressing E2F1 sh-RNA (P3HR1-sh-E2F1) in the absence and presence of doxycycline (-/ + DOX) either left untreated or or treated with TPA-NaBu for 72 h. (B) EBV intracellular or extracellular genome copy number analysis was performed on cDNA isolated from P3HR1-sh-E2F1 cells in the absence and presence of doxycycline (-/ + DOX) either left untreated or treated with TPA-NaBu for 72h. The results are presented as the mean ± SD, n = 3 biological replicates. Statistical significance was determined by a two-sided Student’s t-test, *P < 0.05; **P < 0.01; ***P < 0.001; ns, not significant. (TIF) [file ppat.1013410.s010.TIF]

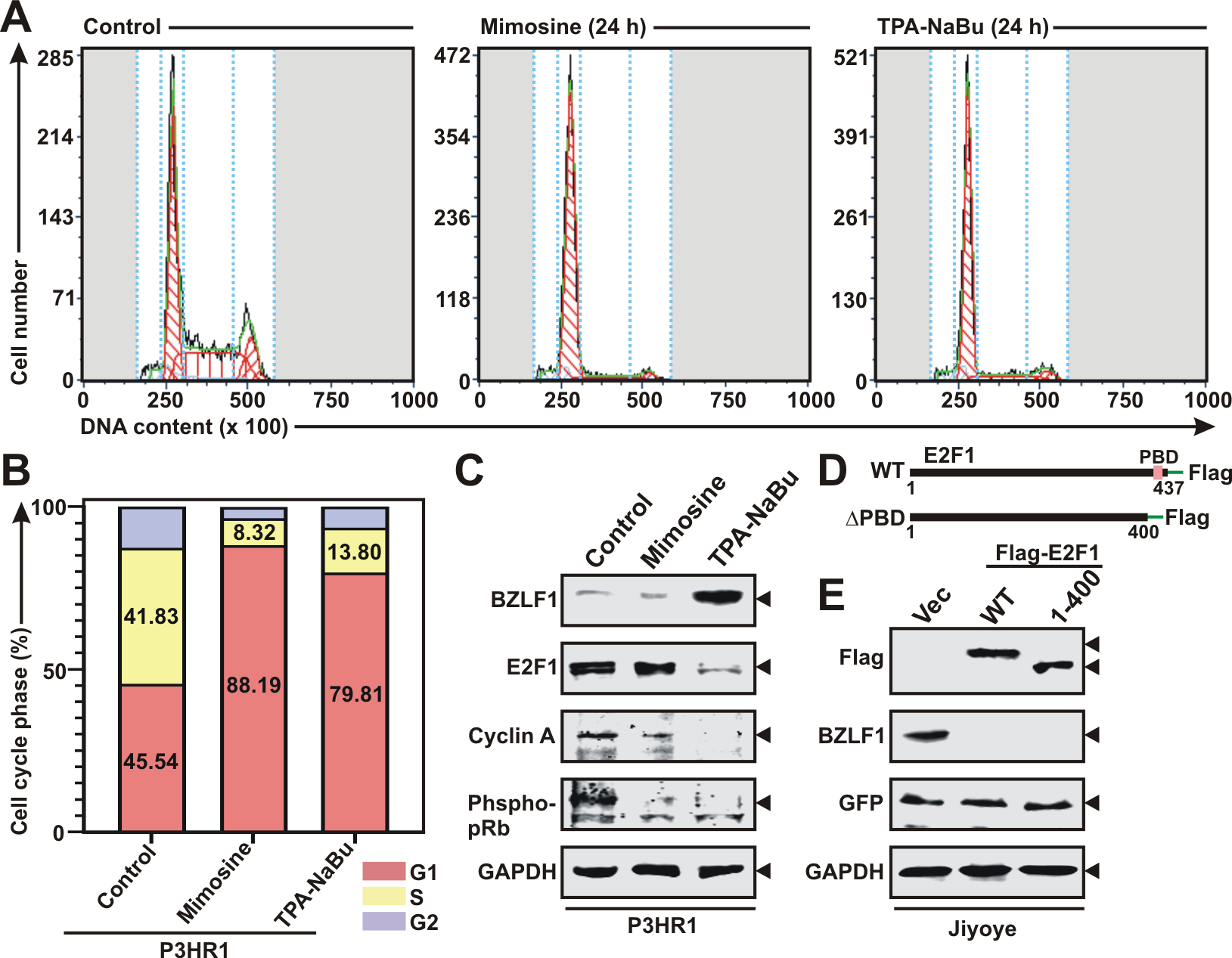

Supplement: S11 Fig — (A) Cell cycle analysis of P3HR1 cells either left untreated or treated with Mimosine or TPA-NaBu for 24 h. (B) Cell cycle phase quantification data of P3HR1 cells either left untreated or treated with Mimosine or TPA-NaBu for 24 h. (C) Immunoblot analysis of P3HR1 cells either left untreated or treated with Mimosine or TPA-NaBu for 24 h. (D) Schema showing deletion of pocket protein binding domain (PBD) of E2F1 for cloning into a flag-tagged expression vector. (E) Immunoblot analysis of Jiyoye cells transiently transfected with control vector, flag-tagged wild-type (residues 1–437) E2F1 or pocket protein binding domain deleted E2F1 (residues 1–400) expression plasmids. Cell cycle distribution graphs and blots are representative of n = 3 biological replicates. (TIF) [file ppat.1013410.s011.TIF]

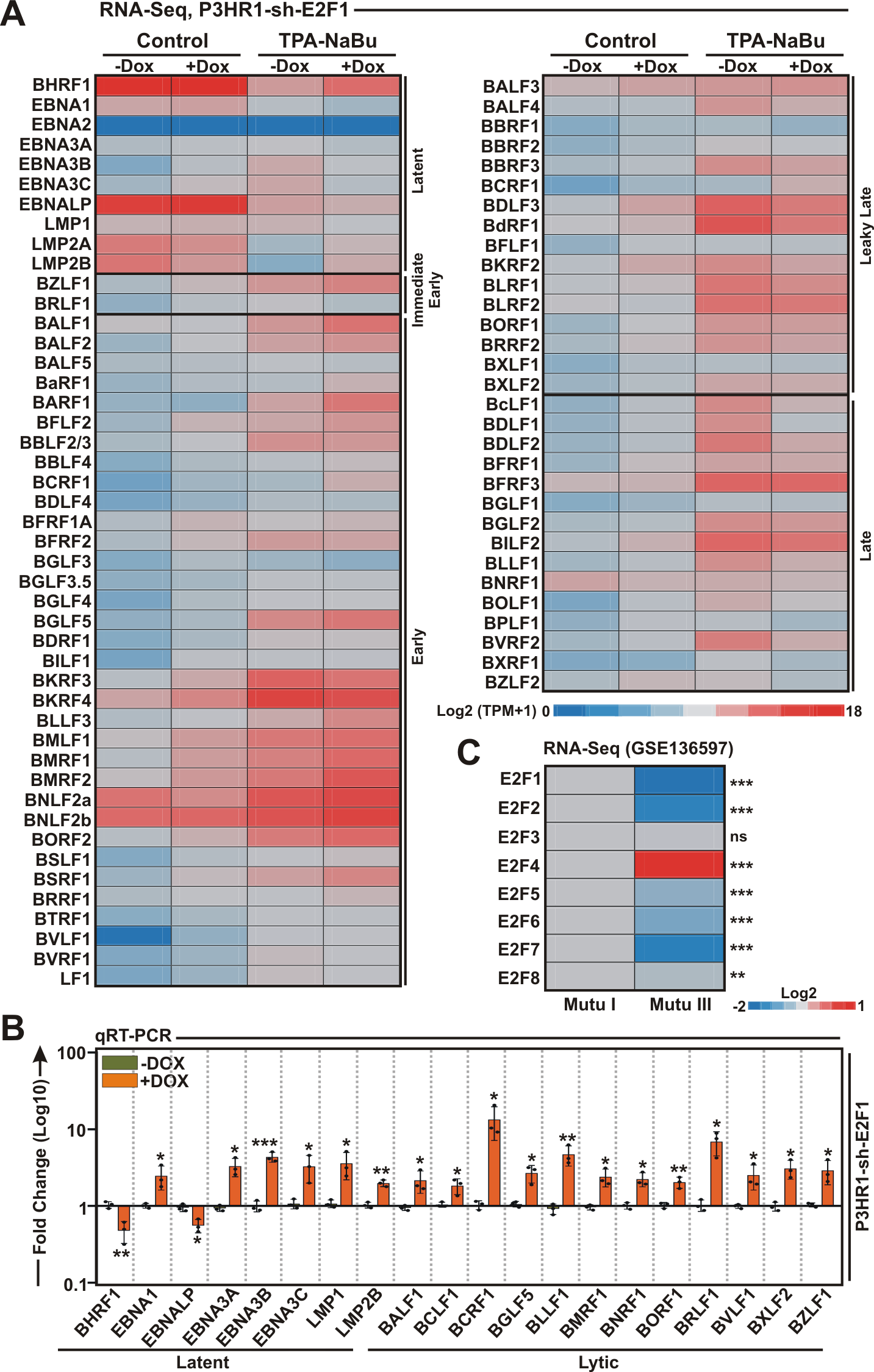

Supplement: S12 Fig — (A) Heatmap analysis of RNA-Seq data of EBV transcripts from P3HR1 cells stably expressing E2F1 sh-RNA (P3HR1-sh-E2F1) in the absence and presence of doxycycline (-/ + DOX) and with or without TPA-NaBu treatment for 72 h. Log2 (TPM + 1) in EBV mRNA abundance are shown. (B) qRT-PCR analysis of EBV latent and lytic gene mRNAs from P3HR1-sh-E2F1 cells in the absence and presence of doxycycline (-/ + DOX). qRT-PCR results are presented as the mean + SD, n = 3 biological replicates. Statistical significance was determined by a two-sided Student’s t-test, *P < 0.05; **P < 0.01; ***P < 0.001; ns, not significant. (C) Reanalysis of RNA-Seq data (GSE136597) of all eight E2F genes (E2F1-8) in EBV+ BL lines Mutu I and Mutu III. (TIF) [file ppat.1013410.s012.TIF]

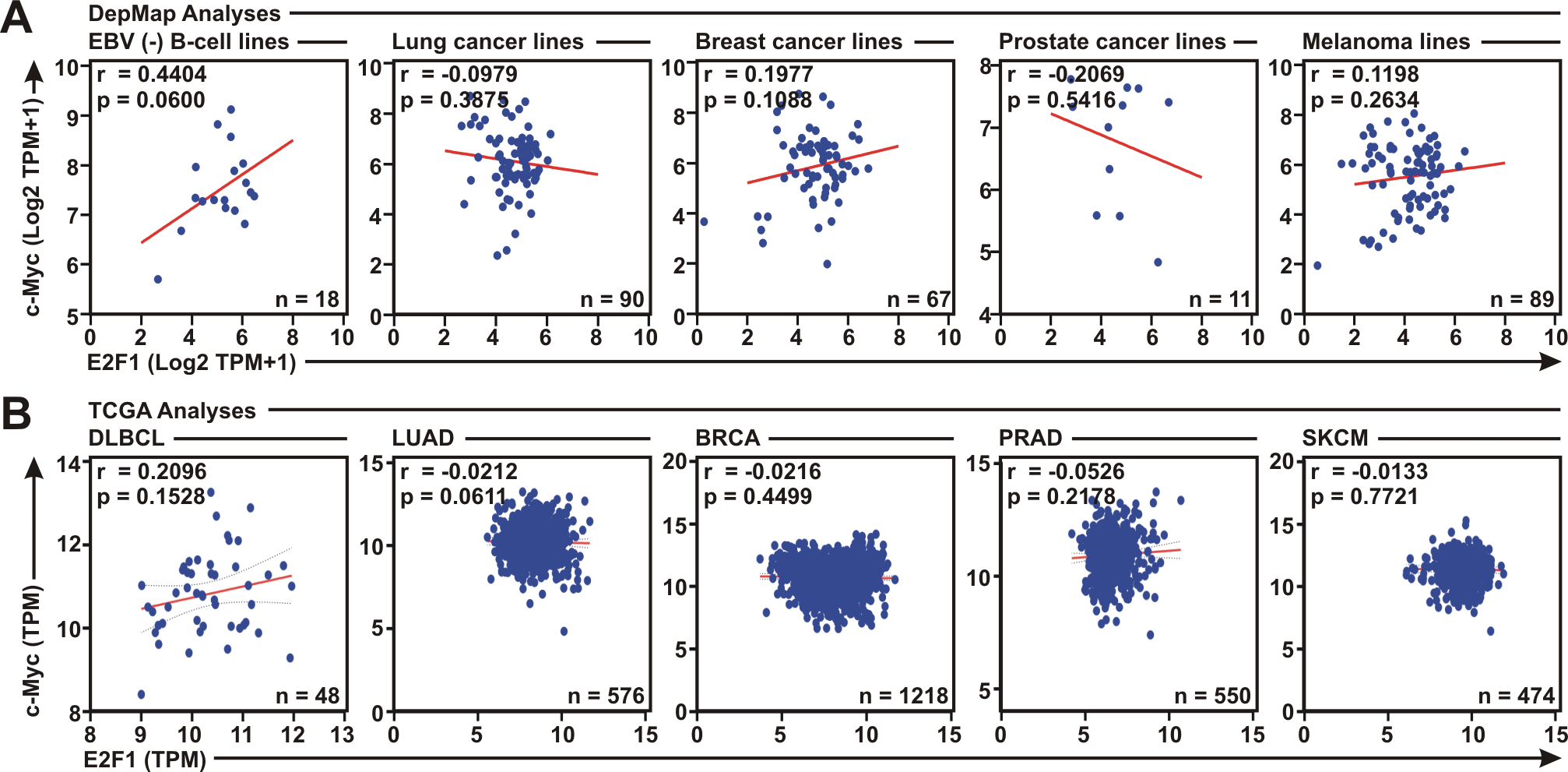

Supplement: S13 Fig — (A) Two-sided unpaired Student’s t-test and Pearson’s correlation were employed to analyse the association between E2F1 and c-Myc transcripts in EBV- B-cell, lung cancer, breast cancer, prostate cancer, and melanoma lines from DepMap portal (https://depmap.org/portal/). (B) Two-sided unpaired Student’s t-test and Pearson’s correlation were employed to analyse the association between E2F1 and c-Myc transcripts in DLBCL (diffuse large B-cell lymphoma), lung adenocarcinoma (LUAD), breast invasive carcinoma (BRCA), prostate adenocarcinoma (PRAD) and skin cutaneous melanoma (SKCM) patients’ tissue samples from TCGA datasets (https://ualcan.path.uab.edu/). (TIF) [file ppat.1013410.s013.TIF]

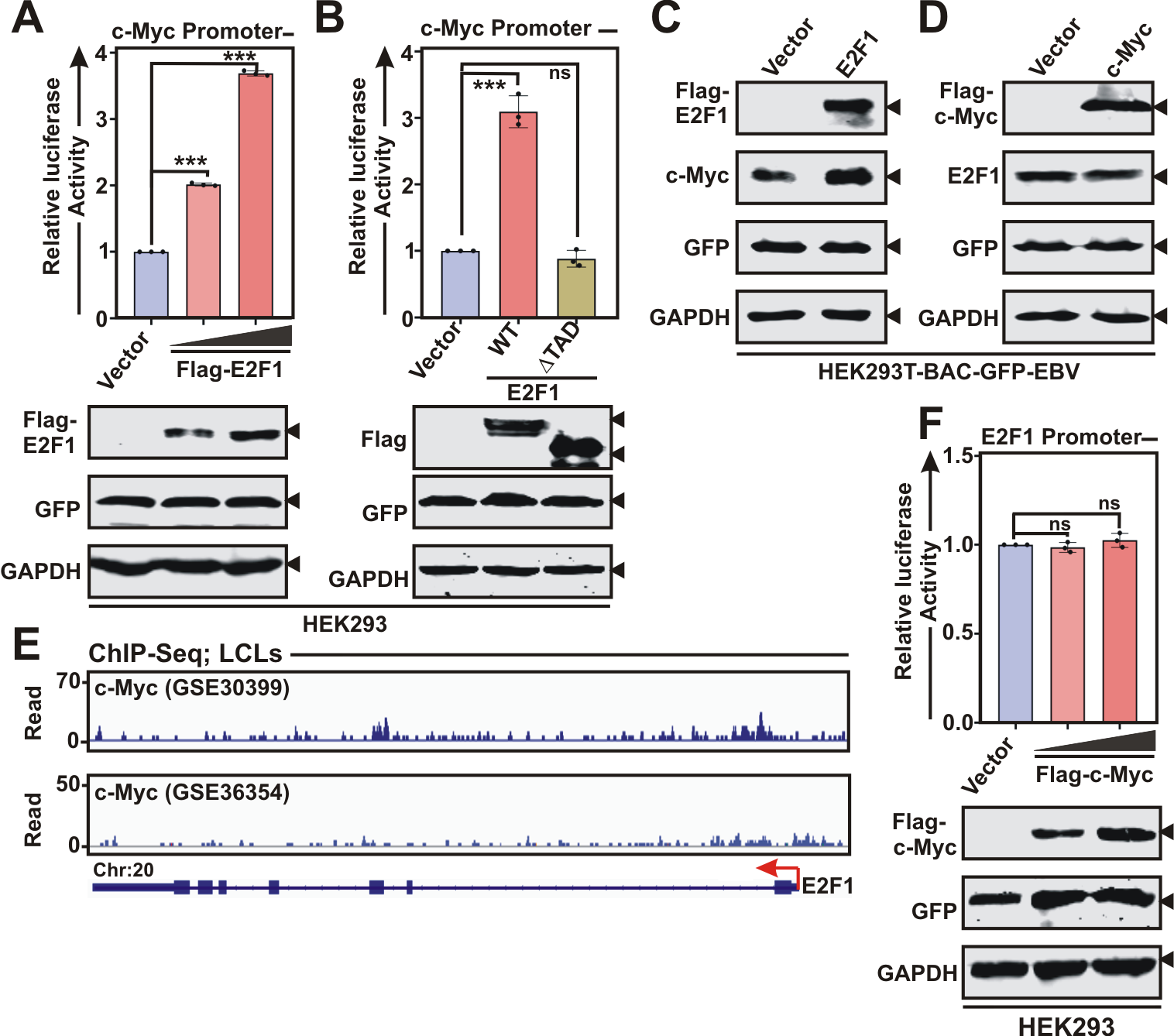

Supplement: S14 Fig — (A) Luciferase reporter activity and the corresponding immunoblot analysis of the wild-type c-Myc promoter in the presence of increasing concentrations of E2F1 expression plasmid in transiently transfected HEK293 cells. (B) Luciferase reporter activity and the corresponding immunoblot analysis of the c-Myc promoter in the presence of empty vector, wild-type (WT) or transactivation domain deleted (ΔTAD) E2F1 expression plasmids in HEK293 cells. (C) Immunoblot analysis of HEK293T-BAC-GFP-EBV cells transiently transfected control vector or flag-tagged E2F1 expression plasmid. (D) Immunoblot analysis of HEK293T-BAC-GFP-EBV cells transiently transfected control vector or flag-tagged c-Myc expression plasmid. (E) Reanalysis of LCLs ChIP-Seq tracks (GSE30399 and GSE36354) of c-Myc at E2F1 promoter region. (F) Luciferase reporter activity and the corresponding immunoblot analysis of the wild-type E2F1 promoter in the presence of increasing concentrations of c-Myc expression plasmid in HEK293 cells. The results are presented as the mean ± SD, n = 3 biological replicates. Statistical significance was determined by a two-sided Student’s t-test, *P < 0.05; **P < 0.01; ***P < 0.001; ns, not significant. (TIF) [file ppat.1013410.s014.TIF]

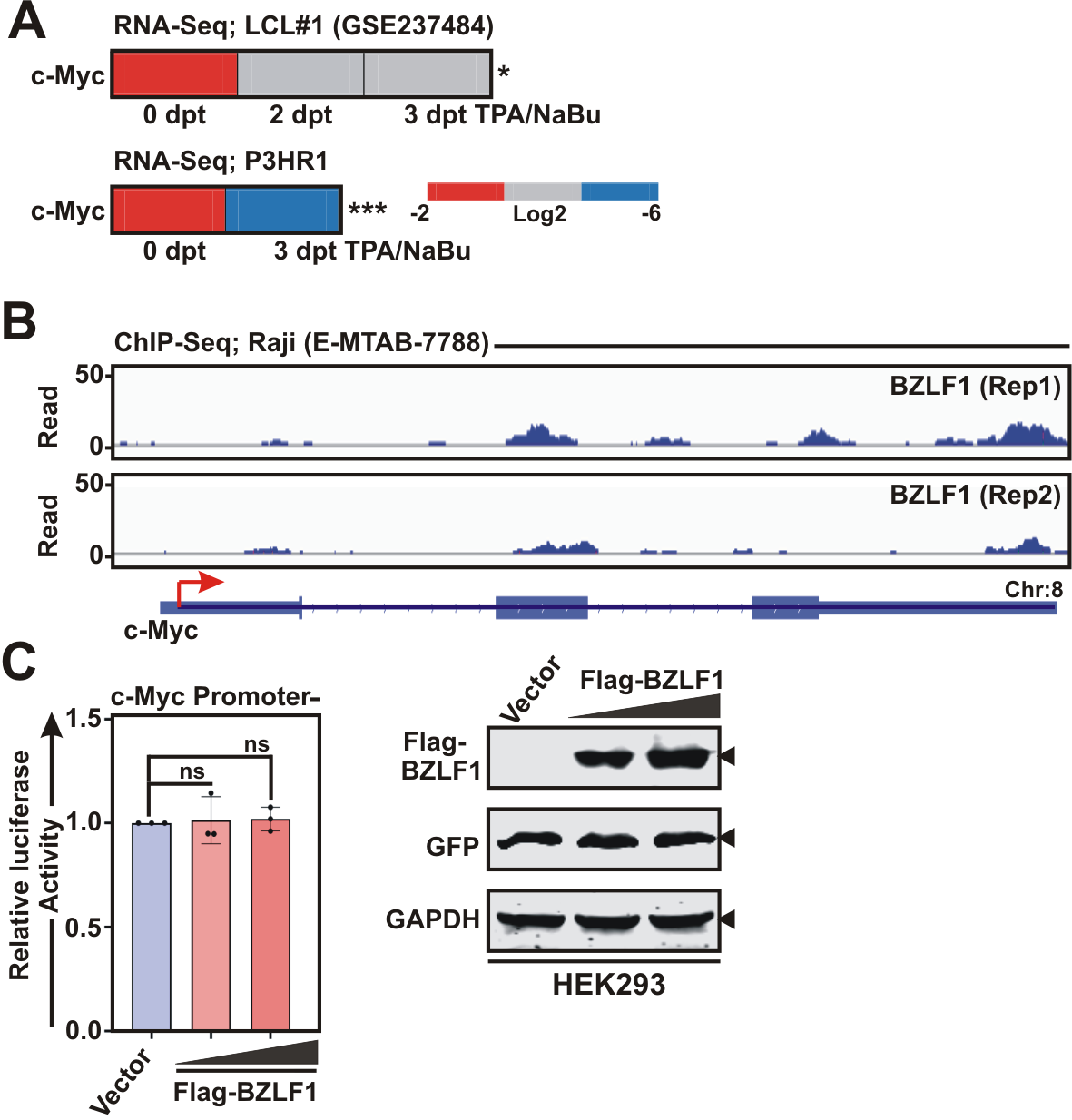

Supplement: S15 Fig — (A) Heat map analysis (log2 Fold Change) of c-Myc transcript of RNA-Seq data (GSE237484) of LCL#1 and P3HR1 cells reactivated to lytic replication by TPA-NaBu treatment for 0–3 days post treatment (dpt). (B) Reanalysis of Raji ChIP-Seq tracks (E-MTAB-7788) of BZLF1 at c-Myc gene locus. (C) Luciferase reporter activity and the corresponding immunoblot analysis of the wild-type c-Myc promoter in the presence of increasing concentrations of BZLF1 expression plasmid in transiently transfected HEK293 cells. The results are presented as the mean + SD, n = 3 biological replicates. Statistical significance was determined by a two-sided Student’s t-test, *P < 0.05; **P < 0.01; ***P < 0.001; ns, not significant. (TIF) [file ppat.1013410.s015.TIF]
