# Supplementary material for: E2F1 suppresses Epstein-Barr virus lytic reactivation through cellular and viral transcriptional networks
Source: PLoS Pathog. 2025 Aug 7;21(8):e1013410. doi: 10.1371/journal.ppat.1013410 (PMC12349880; doi:10.1371/journal.ppat.1013410)
Supplement: S1 Table — (DOCX) [file ppat.1013410.s016.docx]

| **Accession ID** | **Experiment** | **Target** | **Cell line** |
| --- | --- | --- | --- |
| GSE73887 | ChIP-Seq | EBNA1 | LCL |
| GSE29498 |  | EBNA2 | IB4 LCL |
| GSE49338 |  | EBNALP |  |
| GSE88729 |  | EBNA3A | LCL |
|  |  | EBNA3B |  |
|  |  | EBNA3C |  |
| E-MTAB-7788 |  | BZLF1 | Raji |
| GSE55105 |  | RelA | GM12878 LCL |
|  |  | RelB |  |
|  |  | cRel |  |
|  |  | p50 |  |
|  |  | p52 |  |
| GSE76191 |  | E2F1 | Raji |
| GSE30399 |  | c-Myc | GM12878 LCL |
| GSE36354 |  | c-Myc | P493-6 LCL |
| GSE237484 | RNA-Seq | | LCLs |
| GSE125974 |  |  | B cells |
| GSE235941 |  |  | PBMCs |
| GSE140653 |  |  | Akata |
| GSE136597 |  |  | Mutu I-III |
| GSE231687 |  |  | HK1-EBV |
| GSE155811 |  |  | AGSiZ |
| NA |  |  | LCLs |
| GSE272763 | scRNA-Seq | | P3HR1-ZHT |
| E-MEXP-2767 | Micro Array | | BL31 |

**S1 Table.** Previously published datasets used in this study.
