# Supplementary material for: E2F1 suppresses Epstein-Barr virus lytic reactivation through cellular and viral transcriptional networks
Source: PLoS Pathog. 2025 Aug 7;21(8):e1013410. doi: 10.1371/journal.ppat.1013410 (PMC12349880; doi:10.1371/journal.ppat.1013410)
Supplement: S2 Table — (DOCX) [file ppat.1013410.s017.docx]

**Table S3.** All E2F’s Structural and Sequence Similarity.

| **Protein** | **AlphaFold DB ID** | **RMSD** | **TM-score** | **Sequence similarity^#^** | **Aligned residue** | **Sequence Length** |
| --- | --- | --- | --- | --- | --- | --- |
| **E2F1*** | AF-Q01094-F1-v4 | - | - | - | - | 437 |
| **E2F2** | AF-Q14209-F1-v4 | 4.62 | 0.38 | 53% | 158 | 437 |
| **E2F3** | AF-O00716-F1-v4 | 5.61 | 0.48 | 39% | 187 | 465 |
| **E2F4** | AF-Q16254-F1-v4 | 5.41 | 0.30 | 22% | 115 | 413 |
| **E2F5** | AF-Q15329-F1-v4 | 4.17 | 0.29 | 22% | 116 | 346 |
| **E2F6** | AF-O75461-F1-v4 | 3.87 | 0.35 | 31% | 141 | 281 |
| **E2F7** | AF-Q96AV8-F1-v4 | 5.96 | 0.22 | 23% | 77 | 911 |
| **E2F8** | AF-A0AVK6-F1-v4 | 4.96 | 0.21 | 27% | 85 | 867 |

* E2F1 is used as a reference protein

^#^ UniProt Align tool (<https://www.uniprot.org/align>) was used for sequence similarity
