# Supplementary material for: E2F1 suppresses Epstein-Barr virus lytic reactivation through cellular and viral transcriptional networks
Source: PLoS Pathog. 2025 Aug 7;21(8):e1013410. doi: 10.1371/journal.ppat.1013410 (PMC12349880; doi:10.1371/journal.ppat.1013410)
Supplement: S6 Table — (DOCX) [file ppat.1013410.s021.docx]

**Table S4.** Oligo sequences used for qRT-PCR, cloning of Sh-RNAs, sgRNAs, cDNAs and promoter regions and ChIP-qPCR.

| **Reagent or Resource** | **Source** | **Identifier** |
| --- | --- | --- |
| **qRT-PCR oligonucleotides** | | |
| GAPDH qRT-PCR  Fw- 5’-AATGAAGGGGTCATTGATGG-3’  Rv- 5’-AAGGTGAAGGTCGGAGTCAA-3’ | PMID: 38530845 | N/A |
| B2M qRT-PCR  Fw- 5’-GAGGCTATCCAGCGTACTCCA-3’  Rv- 5’-CGGCAGGCATACTCATCTTTT-3’ | PMID: 38530845 | N/A |
| EBNA1 qRT-PCR  Fw- 5’-CATTGAGTCGTCTCCCCTTTGGAAT-3’  Rv- 5’-TCATAACAAGGTCCTTAATCGCATC-3’ | PMID: 38530845 | N/A |
| EBNA3A qRT-PCR  Fw- 5’-CCTAAAGATGGACGAGGGGC-3’  Rv- 5’-CAAACTGGTCGCTCAAGTGC -3’ | This study | N/A |
| EBNA3B qRT-PCR  Fw- 5’-CCCTTGCGGATGCAGCCAAT-3’  Rv- 5’-GGCTGATATGGAATGTGCCC-3’ | PMID: 38530845 | N/A |
| EBNA3C qRT-PCR  Fw- 5’-CTGCAGCCCAGAGAGTAGTC -3’  Rv- 5’-TCCATGGTGGGTCTTAAAGG-3’ | This study | N/A |
| EBNALP qRT-PCR  Fw- 5’-CGGACAGCTCCTAAGAAGGC -3’  Rv- 5’-CCACCTCCTCTTCTTGCTGG-3’ | This study | N/A |
| LMP1 qRT-PCR  Fw- 5’-CCCGCACCCTCAACAAGCTACCGAT-3’  Rv- 5’-TTGTCAGGACCACCTCCAGGTGCGC-3’ | PMID: 38530845 | N/A |
| LMP2A qRT-PCR  Fw- 5’-CTCTCACTTCTACTCTTGCGAG-3’  Rv- 5’-AGTCAAACGGCGCCATCTCCTT -3’ | This study | N/A |
| LMP2B qRT-PCR  Fw- 5’-TGGCGGCATCATGTTTTTGG -3’  Rv- 5’-TAGCAGCAGCGTCATGGAAA -3’ | This study | N/A |
| BHRF1 qRT-PCR  Fw- 5’-TTTCACCGTGGAGACCCAAG -3’  Rv- 5’-CACGAACTGACAGGTCCACA -3’ | This study | N/A |
| BZLF1 qRT-PCR  Fw- 5’- CCAGGTTGAGGTGCTTCTCCCCCGG-3’  Rv- 5’-AACCGCTCCGACTGGGTCGTGGTTT-3’ | PMID: 38530845 | N/A |
| BRLF1 qRT-PCR  Fw- 5’-AACAGATGACTTGCCTCGGG -3’  Rv- 5’-AGAGGATCAGGCCCTTCCAT -3’ | This study | N/A |
| BMRF1 qRT-PCR  Fw- 5’-CTGCCCCCTGTCTCTTTCTG -3’  Rv- 5’-CATGAAGACAGCCGACACCT -3’ | This study | N/A |
| BGLF5 qRT-PCR  Fw- 5’-AAGCCAAATTGACGGTTGGC-3’  Rv- 5’-GTTTGAGCCAGCTCCCATCT -3’ | This study | N/A |
| BcRF1 qRT-PCR  Fw- 5’-AAGGGATGCCTTCAGTCGTG -3’  Rv- 5’-TCAGGGTCCTGGTTTTCAGC -3’ | This study | N/A |
| BVLF1 qRT-PCR  Fw- 5’-AGAGCAGATTTCCGTCAGCC -3’  Rv- 5’-CGCTCACGGTCTCTTTGTCT-3’ | This study | N/A |
| BBRF1 qRT-PCR  Fw- 5’-CCCAAGAGCTCCACTCCAAG -3’  Rv- 5’-ACTCGATGGTCACCTCCGTA -3’ | This study | N/A |
| BXLF2 qRT-PCR  Fw- 5’-CAGCTTGTCTCGTGTGAGGT -3’  Rv- 5’- ATACCAACTGCTGTCCACCG-3’ | This study | N/A |
| BORF1 qRT-PCR  Fw- 5’-GCCTCTATGTCGCTCTGACC -3’  Rv- 5’-TATGTAGGGGCTGGACCACA -3’ | This study | N/A |
| BOLF1 qRT-PCR  Fw- 5’-GGTCTCAACCTTCTGGGCTC -3’  Rv- 5’-GGACTCCTCCCCATTCTCCT -3’ | This study | N/A |
| BLLF1 qRT-PCR  Fw- 5’-TTGTATGGGGTGTCAGCAGG -3’  Rv- 5’-TCTCCATTTTACCCAGCGTG -3’ | This study | N/A |
| BNRF1 qRT-PCR  Fw- 5’-GTCTTGCTCACCCTGCATCT -3’  Rv- 5’-AGGCCACCACAGTCATGAAG -3’ | This study | N/A |
| BALF1 qRT-PCR  Fw- 5’-GCTGGCCTTGAGGGCGCTGAGGACT -3’  Rv- 5’- CACCCACGGAAGCCCTCTGGACTTC-3’ | This study | N/A |
| BCLF1 qRT-PCR  Fw- 5’-CCTCTTGGAATGCAGCTGGGGCCAG -3’  Rv- 5’-CCAATTATGACCTGCTGCGGCTGGA -3’ | This study | N/A |
| E2F1 qRT-PCR  Fw- 5’-GCCATCCAGGAAAAGGTGTGA-3’  Rv- 5’-GTGATGTCATAGATGCGCCG-3’ | This study | N/A |
| c-Myc qRT-PCR  Fw- 5’-AGTGGAAAACCAGCAGCCTC -3’  Rv- 5’-TTCTCCTCCTCGTCGCAGTA -3’ | This study | N/A |
| **Cloning oligonucleotides** | | |
| sg-Myc#1  Fw- 5’-CACCGGGTAGGGGAAGACCACCGAG-3’  Rv- 5’-AAACCTCGGTGGTCTTCCCCTACCC-3’ | PMID: 32315601 | N/A |
| sg-Myc#2  Fw- 5’-CACCGTTGAGGGGCATCGTCGCGGG -3’  Rv- 5’-AAACCCCGCGACGATGCCCCTCAAC -3’ | This study | <https://www.synthego.com/products/bioinformatics/crispr-design-tool> |
| sg-E2F1#1  Fw- 5’-CACCGGGAGATGATGACGATCTGCG -3’  Rv- 5’-AAACCGCAGATCGTCATCATCTCCC -3’ | PMID: 28211871 | N/A |
| sg-E2F1#2  Fw- 5’-CACCGCCCATGCGCGCCGGCGCTGG -3’  Rv- 5’-AAACCCAGCGCCGGCGCGCATGGGC -3’ | This study | <https://www.synthego.com/products/bioinformatics/crispr-design-tool> |
| Sh-E2F1  Fw- 5’-TCGAGTGCTGTTGACAGTGAGCGACACTGAATCTGACCACCAATAGTGAAGCCACAGATGTATTGGTGGTCAGATTCAGTGGTGCCTACTGCCTCGGAA-3’  Rv- 5’-TTCCGAGGCAGTAGGCACCACTGAATCTGACCACCAATACATCTGTGGCTTCACTATTGGTGGTCAGATTCAGTGTCGCTCACTGTCAACAGCACTCGA-3’ | - PMID: 26510456 | N/A |
| pGL3-Lmp1p cloning primer  Fw- 5’-ATATGGTACCCAAGGAGGGCTCCTCCAG -3’  Rv- 5’-ATATAAGCTTCCGCGCCTCTTTGTGCAG -3’ | - This study | N/A |
| pGL3-Lmp2p cloning primer  Fw- 5’-AGACACGCGTTTCTTCTTGCCCGTTCTC -3’  Rv- 5’-ATGCAGATCTACAGAGGCACACTAACCG -3’ | - This study | N/A |
| pGL3-Cp cloning primer  Fw- 5’-ATATACGCGTCTAGAGACCGCCAAGATG -3’  Rv- 5’-AGATAGATCTCCTTAGTCCCCCCCTTA -3’ | - This study | N/A |
| pGL3-Wp cloning primer  Fw- 5’-ATCTACGCGTTTGGACCCGAAATCTGA -3’  Rv- 5’-ATGTAGATCTCGGGCCGGGTTGGTCCA -3’ | - This study | N/A |
| pGL3-Qp cloning primer  Fw- 5’-ATATACGCGTCTAGCCTGACTAAGGGTGAG -3’  Rv- 5’-ATATAGATCTGTGCCAGGCCCGTGCCAAAG -3’ | - This study | N/A |
| pGL3-BZLF1p cloning primer  Fw- 5’-ATATACGCGTTTACGAAACGCGTGTTT -3’  Rv- 5’-AGATAGATCTCTAAGCAGATCTACAGGA -3’ | - This study | N/A |
| pGL3-E2F1p cloning primer  Fw- 5’-ATATACGCGTTCCTGCACCCAGCCCTATCC -3’  Rv- 5’-AGACAGATCTCAGGAGCTGTGATTTGAGGC -3’ | - This study | N/A |
| pA3F-E2F1 del TAD cloning primer  Fw- 5’-ATATGAATTCATGGCCTTGGCCGGGGC -3’  Rv- 5’-ATATGCGGCCGCAGCCCGCAGGCTGCC -3’ | - This study | N/A |
| pGL3-E2F1p Mut1 cloning primer  Fw- 5’-CTGCTTGAACAAAAAAATTCCCTCACTCATTC -3’  Rv- 5’-ATGCTATACGTTGGC -3’ | - This study | N/A |
| pGL3-E2F1p Mut2 cloning primer  Fw- 5’-ATTCCCTCACAAAAAAAACAAACGTTTGGAG -3’  Rv- 5’-GAATGAGTTCAAGCAGATG -3’ | - This study | N/A |
| pGL3-E2F1p Mut3 cloning primer  Fw- 5’-TCCCAAGGATAAAAAAACTTCCTCTATTGCCC -3’  Rv- 5’-GGGCTCCCTAGAAGTG -3’ | - This study | N/A |
| pGL3-BZLF1p Mut1 cloning primer  Fw- 5’-AGCAAAGGTGAAAAAAAAGGTGCAATG -3’  Rv- 5’-ATCTTTGCTGAAGATGATG -3’ | - This study | N/A |
| pGL3-BZLF1p Mut2 cloning primer  Fw- 5’-TTAAAGCCAAAAAAAAAGCCTCCTCTGTGATG -3’  Rv- 5’-AGGGGAGATGTTAGAC -3’ | - This study | N/A |
| pGL3-BZLF1p Mut3 cloning primer  Fw- 5’-GCCACGAGGGAAAAAATGGCTCAGGTC -3’  Rv- 5’-CATTTGGACGAACTGAC -3’ | - This study | N/A |
| pGL3-c-Mycp cloning primer  Fw- 5’- ATATACGCGTCGCCCACCGGCCCTTTA -3’  Rv- 5’- ATATAGATCTGTGCAAAGTGCCCGCCC -3’ | - This study | N/A |
| pGL3-c-Mycp Mut-1 cloning primer  Fw- 5’-AAAAAAAGAACGGAGGGAGGGATC -3’  Rv- 5’- TTTTTTTCCTCTGAGAAGCCCTG -3’ | - This study | N/A |
| pGL3-c-Mycp Mut-2 cloning primer  Fw- 5’- AAAAAATTTGCACAGATCTGC -3’  Rv- 5’- TTTTTCTGCTATGGGCAAAG -3’ | - This study | N/A |
| pA3F-E2F1-TAD2 cloning primers  E2F1 DBD1 primers  Fw- 5’-ATATGAATTCATGGCCTTGGCCGGGGC -3’  Rv- 5’- GGTTGGCGCTGGTGCAGCCCGCAGGCTGCC -3’  Assembly PCR primers  E2F2 TAD2  Fw- 5’- GGCAGCCTGCGGGCTGCACCAGCGCCAACC -3’  Rv- 5’- AGTC GCGGCCGC ATTAATCAACAGGTCCCCAAGG -3’ | - This study | N/A |
| pA3F-E2F2-TAD1 cloning primers  E2F2 DBD2 primers  Fw- 5’- ATAT GAATTCATGCTGCAAGGGCCCCG -3’  Rv- 5’- GTCCTCGTCCACGGGTGGCACTGAGGATGC-3’  Assembly PCR primers  E2F1 TAD1  Fw- 5’- GCATCCTCAGTGCCACCCGTGGACGAGGAC -3’  Rv- 5’-ATATGCGGCCGCGAAATCCAGGGGG -3’ | - This study | N/A |
| pA3F-BZLF1- Δ TAD cloning primers  Fw- 5’-CAGTGTGGTGGAATTCTCGCTGGAGGAATGCGA -3’  Rv- 5’- TAGACTCGAGCGGCCGCGAAATTTAAGAGATCCTCGTGTAAA-3’ | - PMID: 38530845 | N/A |
| pA3F-BZLF1- Δ bZIP cloning primers  Fw- 5’- CAGTGTGGTGGAATTCATGATGGACCCAAACTCGAC -3’  Rv- 5’- TAGACTCGAGCGGCCGCTATTTCTAGTTCAGAATCGCATTCC -3’ | PMID: 38530845 | N/A |
| pLVX-TetOne-Puro-BZLF1 cloning primers  Fw- 5’- GCGCGAATTCATGATGGACCCAAACTCG -3’  Rv- 5’- GCGCGGATCCGAAATTTAAGAGATCCTCGTG -3’ | - This study | N/A |
| pA3F-BRLF1 cloning primers  Fw- 5’- GCGCGAATTCTCAAAAAGCAGCTGGGC -3’  Rv- 5’- CGATGCGGCCGCAAATAAGCTGGTGTCAAAAA -3’ | - This study | https://www.addgene.org/72635/ |
| **ChIP oligonucleotides** | | |
| BZLF1 binding E2F1p-1 ChIP-qPCR  Fw- 5’- CGGCGGTTCCTATTGGCTTT -3’  Rv- 5’-CCTGGTACCATCCGGACAAA -3’ | - This study | N/A |
| BZLF1 binding E2F1p-2 ChIP-qPCR  Fw- 5’-TGAAGCCAACCAGTGACCAA -3’  Rv- 5’-TTCTCTTGCTCTCTGCCAGC -3’ | - This study | N/A |
| E2F1 binding C-Mycp-1 ChIP-qPCR  Fw- 5’-GGACAAGGATGCGGTTTGTC -3’  Rv- 5’-TAACTACGCGCGCCTACCAT -3’ | - This study | N/A |
| E2F1 binding c-Mycp-2 ChIP-qPCR  Fw- 5’-GAATAGGGGGCTTCGCCTC -3’  Rv- 5’-GTCCTTGCTCGGGTGTTGTA -3’ | - This study | N/A |
| Cp ChIP-qPCR  Fw- 5’-GGCGGGAGAAGGAATAACG -3’  Rv- 5’-CTTGAGCTCTCTTATTGGCTATAATCC -3’ | - PMID: 32424339 | N/A |
| Wp ChIP-qPCR  Fw- 5’-AGTGGGCTTGTTTGTGACT -3’  Rv- 5’-TGACAATTGGCTGCTGTCT -3’ | - PMID: 32424339 | N/A |
| Qp ChIP-qPCR  Fw- 5’-AAATTGGGTGACCACTGAGGGAGT -3’  Rv- 5’-ATAGCATGTATTACCCGCCATCCG -3’ | - PMID: 32424339 | N/A |
| Lmp1p ChIP-qPCR  Fw- 5’-AGGCAGTTGAGG AAAGAAGG -3’  Rv- 5’-GGCCTACATCCCAAGAAACA -3’ | - PMID: 32424339 | N/A |
| Lmp2p ChIP-qPCR  Fw- 5’-AAGCGGGCAGAGGAAGTATG -3’  Rv- 5’-AAAGAGGTAGGGCGCAACAA -3’ | - PMID: 32424339 | N/A |
| OriLytL ChIP-qPCR  Fw- 5’-CCCTCAGTGTTCGCCAGCTT -3’  Rv- 5’-AGGGTGGGAGCAATTCCAACAC -3’ | - PMID: 33109754 | N/A |
| OriLytR ChIP-qPCR  Fw- 5’-CGCTGGTTAAGCTGACGACCT -3’  Rv- 5’-GCCCTGGCTAGGAAAGGGAGGAA -3’ | - PMID: 33109754 | N/A |
| BZLF1p/Zp ChIP-qPCR  Fw- 5’-GCAAGGTGCAATGTTTAGTGAGTT -3’  Rv- 5’-GCTGGTGCCTTGGCTTTAAAG -3’ | - PMID: 32424339 | N/A |
